# Pnictogen-bonding-crosslinked polymer networks: constructing self-healing materials

**DOI:** 10.1039/d6sc00802j

**Published:** 2026-02-27

**Authors:** Qingli Song, Yi Liu, Yao Wang, Wei Wang

**Affiliations:** a Department of Chemistry, Zhengzhou University Zhengzhou 450001 China wangwei_chem@zzu.edu.cn; b School of Chemistry and Chemical Engineering, Key Laboratory of the Colloid and Interface Chemistry, Ministry of Education, Shandong University Jinan 250100 China yaowang@sdu.edu.cn; c Shenzhen Research Institute of Shandong University A301 Virtual University Park in South District of Shenzhen China

## Abstract

Herein, we introduce pnictogen bonding interaction into polymer networks for the design and modulation of dynamic macromolecular materials. Several types of polymeric pnictogen-bonding networks with graded interaction strengths were constructed to explore the structure–property relationship. Comprehensive investigations revealed that strengthening the pnictogen bonding significantly enhances the topological stability of the resulting materials. In contrast, analogous hydrogen-bonded networks did not exhibit comparable mechanical reinforcement. Moreover, the pnictogen-bonding networks endow the materials with tunable self-healing capability, allowing not only spontaneous healing at room temperature and thermally triggered healing on demand, but also effective healing in aqueous environments. This represents the first exploration of self-healing behavior driven by pnictogen bonding in polymeric materials. Mechanistic insights into the role of pnictogen bonding in polymer networks were elucidated through NMR titration of donor–acceptor polymer pairs, comparative self-assembly behavior, and cocrystal structures of small-molecule analogues. The incorporation of pnictogen bonding interaction into polymer networks provides a robust and versatile platform for engineering high-performance dynamic polymeric materials.

## Abstract 2

The pnictogen-bonding interaction is presented for the first time as a novel crosslinking motif for constructing dynamic polymer networks with enhanced topological stability and tunable self-healing performance.

## Graphical abstract

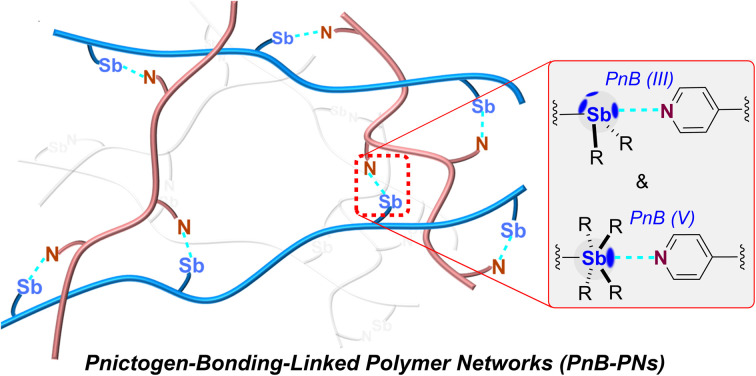

## Introduction

Crosslinking between polymer chains enables the construction of polymer networks (PNs) with distinct mechanical and functional properties. PNs formed *via* permanent covalent bonds exhibit excellent strength and durability; however, the irreversible nature of these crosslinks restricts molecular-level rearrangement, thereby precluding self-healing and reprocessability.^1^ In contrast, dynamically crosslinked PNs based on reversible bonds offer topological adaptability, allowing bond exchange under specific stimuli. This dynamic behavior imparts viscoelasticity that bridges solid-like and liquid-like characteristics, and translates macroscopically into intelligent functions such as self-healing, shape reconfiguration, and stimulus responsiveness.^2^ Dynamically crosslinked PNs are broadly classified into covalent and non-covalent types. Dynamic covalent PNs, the most extensively studied class,^3^ utilize reversible covalent reactions (*e.g.*, Diels–Alder cycloaddition,^4^ disulfide exchange,^5^ transesterification,^6^ and imine exchange^7^) to enable network rearrangement. Although dynamic covalent PNs exhibit superior mechanical robustness, the inherently slow dissociation kinetics of dynamic covalent bonds often lead to sluggish responses under mild, catalyst-free conditions. Conversely, non-covalent bonds typically possess lower bond energies and faster dissociation kinetics. These attributes facilitate rapid network reorganization under benign conditions without catalysts, endowing materials with exceptional intrinsic self-healing capabilities while typically resulting in compromised mechanical strength.^8^

A wide range of supramolecular interactions, including hydrogen bonding,^9^ host–guest interactions,^10^ ionic interactions,^11^ π–π stacking,^12^ van der Waals forces,^13^ and coordination interactions involving metal centers^14^ or boron-based compounds,^15^ have been exploited to construct dynamic PNs. Among these, hydrogen-bonded polymer networks (HB-PNs) based on donor–acceptor motifs are the most extensively studied.^16^ However, hydrogen bonding suffers from several intrinsic limitations, including the generally weak strength of monodentate interactions, poor tolerance toward polar solvents,^17^ and design constraints imposed by the hard–soft acid–base (HSAB) principle, which restrict the incorporation of soft acceptors into dynamic PNs.^18^

**Fig. 1:**
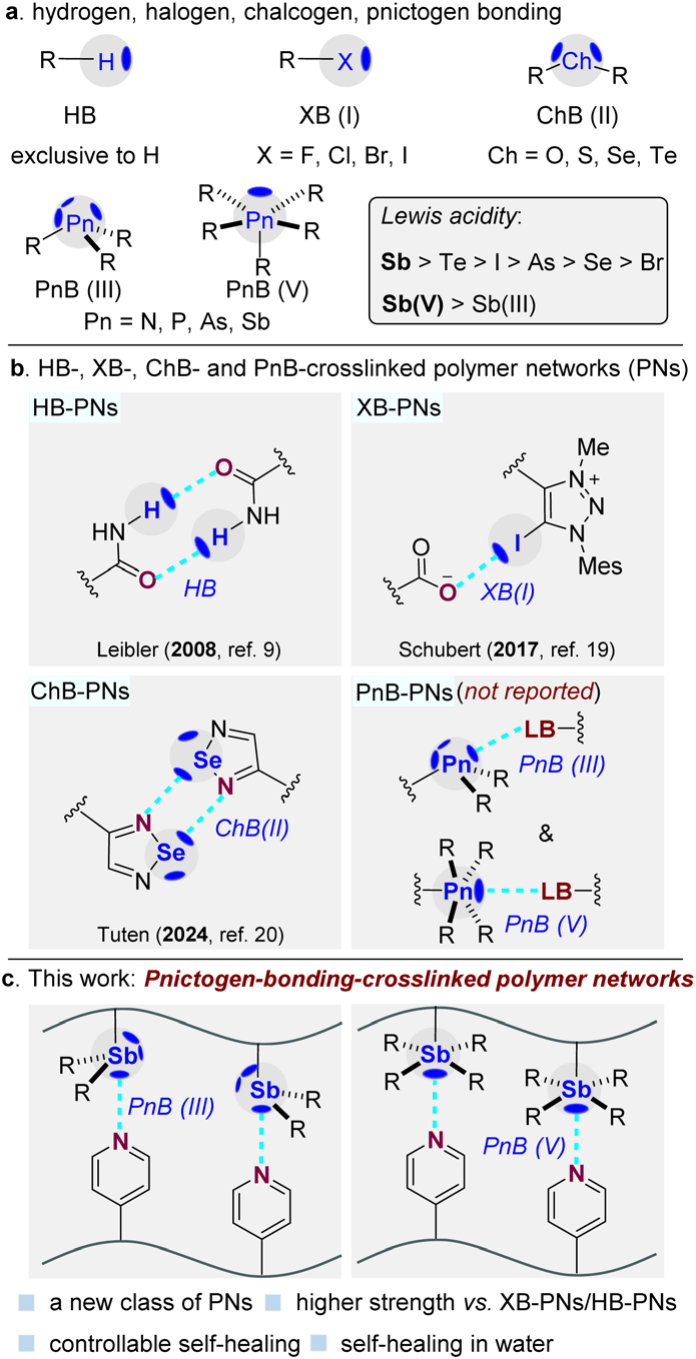
(a) Hydrogen, halogen, chalcogen, and pnictogen bonding (HB, XB, ChB and PnB). (b) HB-, XB-, ChB- and PnB-crosslinked polymer networks. (c) Pnictogen-bonding-crosslinked polymer networks (PnB-PNs).

To address these limitations, nonclassical weak interactions such as halogen bonding (XB) and chalcogen bonding (ChB) have emerged as alternative supramolecular motifs for dynamic polymeric materials (Fig. 1a–b). For example, Schubert and co-workers pioneered the construction of monovalent halogen-bonded polymer networks using polymeric iodo-triazole donors and carboxylate acceptors, which exhibited self-healing behavior at elevated temperatures.^19^ Tuten and Puttreddy independently employed selenadiazole motifs as donors and acceptors to construct divalent chalcogen-bonded polymer networks, endowing the resulting materials with self-healing and shape-memory properties.^20,21^ Despite these important advances, it remains challenging to identify supramolecular interactions that simultaneously offer strong and tunable binding while maintaining rapid reversibility, a prerequisite for bridging the gap between weak noncovalent interactions and irreversible covalent crosslinks in dynamic PNs.

Pnictogen bonding (PnB) has recently emerged as a distinctive noncovalent interaction in supramolecular chemistry.^22^ Compared with HB, XB, and ChB, PnB donors typically possess multiple accessible *σ*-holes, enabling enhanced binding directionality and increased structural complexity (Fig. 1a). In addition, systematic variations in donor polarizability and electronegativity lead to a characteristic trend in Lewis acidity (Sb > Te > I > As > Se > Br),^22*a*^ suggesting that antimony-based donors can form particularly strong yet directional supramolecular interactions. These features render PnB a promising, yet unexplored, motif for constructing robust dynamic polymer networks, especially for regulating macroscopic properties such as self-healing behavior.

Herein, we report the first pnictogen-bonding-crosslinked polymer networks (PnB-PNs) (Fig. 1c). By exploiting directional interactions between electrophilic Sb(III/V) centers and pyridine-functionalized polymer chains, efficient and reversible interchain crosslinking is achieved. Systematic modulation of pnictogen-bonding strength reveals clear correlations between supramolecular interaction strength, network topology, and dynamic material properties, establishing PnB as a versatile platform for the rational design of high-performance dynamic polymeric materials.

## Results and discussion

**Fig. 2:**
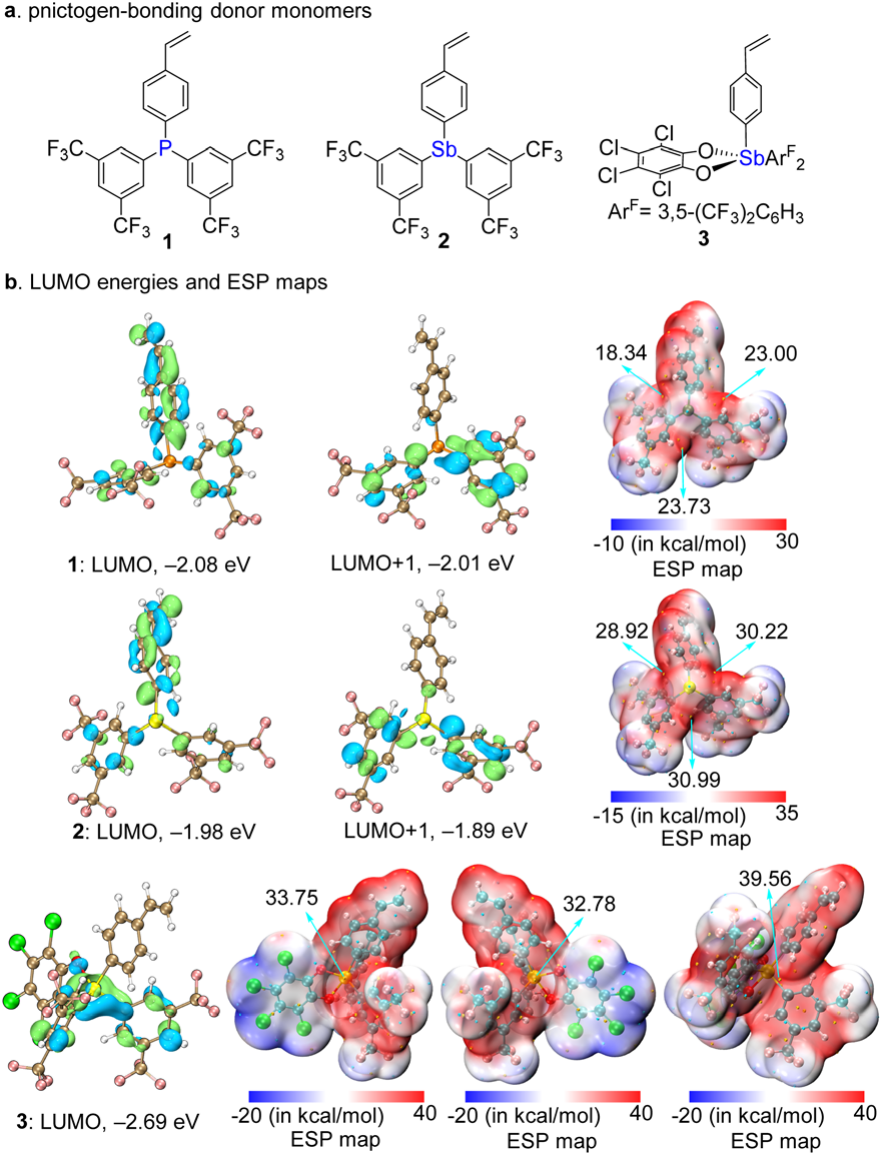
(a) PnB donor monomers. (b) LUMO energies and ESP maps of 1, 2 and 3.

To unequivocally demonstrate the feasibility of constructing PnB-PNs while eliminating potential interferences (*e.g.*, ionic electrostatic interactions and hydrogen bonding involving labile protons), we designed a series of derivatives based on a neutral styrenic backbone lacking both heteroatoms and active hydrogen atoms (Fig. 2a). To minimize structural variations among PnB donors, monomers 1 (P-centered) and 2 (Sb-centered), featuring identical molecular frameworks, were synthesized. In addition, monomer 3 incorporating Sb(v) was prepared to examine the influence of antimony oxidation state on network properties. Computational analyses were conducted to preliminarily assess the PnB donor strength. Electrostatic potential (ESP) mapping revealed three well-defined *σ*-holes in the tetrahedral geometries of monomers 1 and 2, with monomer 2 exhibiting markedly higher maximum positive ESP values than monomer 1, indicative of its enhanced PnB donor strength (Fig. 2b). This increase can be attributed to the higher polarizability and lower electronegativity of Sb relative to P. In contrast, monomer 3, with a high-valent Sb center, shows a further enhancement of the *σ*-hole, reflecting its intrinsically stronger Lewis acidity. In addition to the electrostatic analysis, LUMO energy calculations show that monomer 3 possesses a substantially lower LUMO energy than both 1 and 2, suggesting a significantly enhanced ability to accept electron density from Lewis bases. Taken together, the combined ESP and LUMO analyses establish a clear trend in the PnB donor strength of 3 > 2 > 1 when interacting with the same acceptor, consistent with the expected Lewis acidity order of Sb(v) > Sb(iii) > P(iii).^22*b*^

**Fig. 3:**
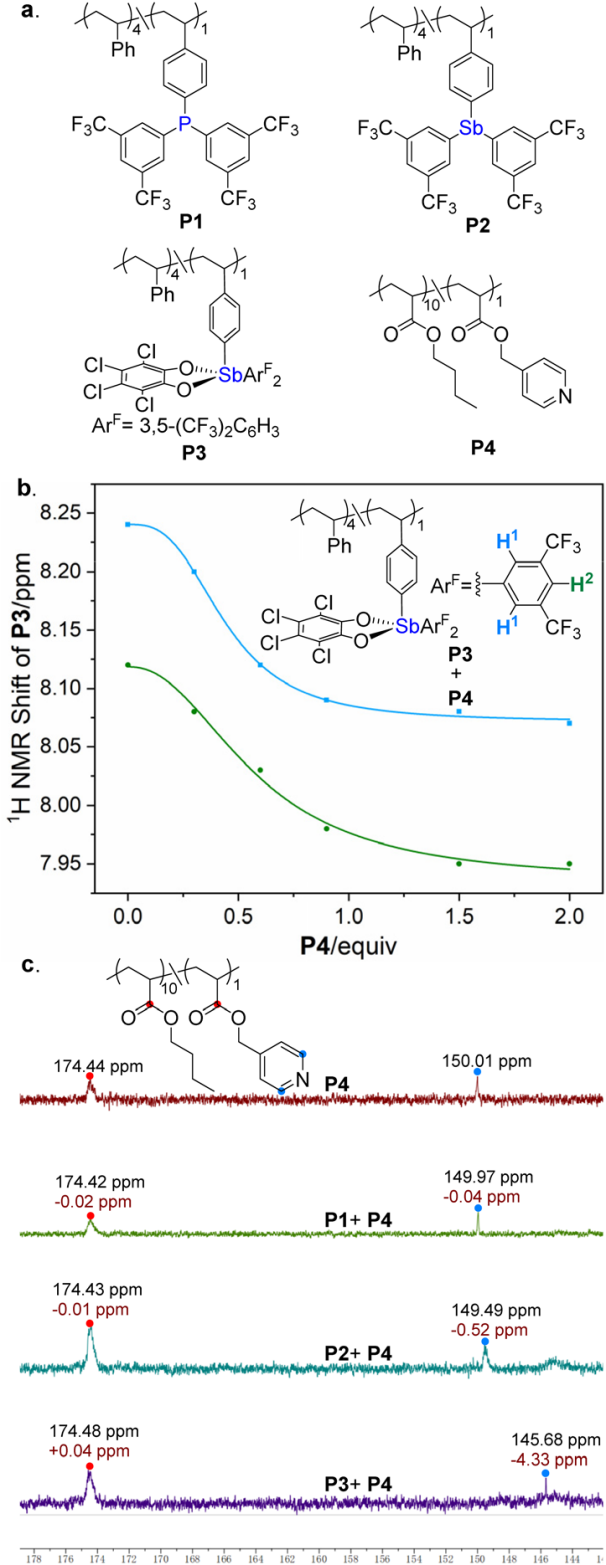
(a) Polymeric PnB donors and acceptors. (b) ^1^H NMR titration curves of PnB donor polymer P3 (0.01 M) and PnB acceptor polymer P4. (c) ^13^C NMR analysis of PnB interaction strengths (PnB donor and acceptor, both 0.01 M).

Guided by computational predictions of PnB donor strength, we initially pursued the synthesis of polymeric PnB donors *via* copolymerization of monomers 1–3 separately with styrene. This approach successfully afforded copolymers P1 and P2 from monomers 1 and 2, respectively (Fig. 3a). However, attempts to polymerize monomer 3 led to complex product mixtures, likely due to decomposition of the high-valent Sb(v) centers under radical polymerization conditions. To overcome this limitation, a post-polymerization modification strategy was employed. Precursor polymer P2 was treated with 3,4,5,6-tetrachlorocyclohexa-3,5-diene-1,2-dione to efficiently generate the Sb(v)-based polymeric donor P3. In parallel, the PnB acceptor polymer was synthesized by RAFT copolymerization of pyridyl methacrylate with butyl acrylate. All copolymers were thoroughly characterized by NMR spectroscopy and elemental microanalysis, confirming their chemical structures. Size exclusion chromatography (SEC) revealed number-average molecular weights (*M*_n_) of approximately 13 000 g mol^−1^ for the target polymers.

To date, no macromolecular pnictogen-bonding polymers have been reported, and thus direct observation of PnB interactions between polymeric donors and acceptors has remained elusive. To investigate such interactions, we performed solution-phase ^1^H NMR titration experiments. In CD_2_Cl_2_, no appreciable chemical shift changes were detected for donor protons in the P1/P4 and P2/P4 systems, even at donor-to-acceptor molar ratios of up to 1 : 10 (Fig. S1 and S3 in the SI), indicating either intrinsically weak binding or the limited detection sensitivity of ^1^H NMR. In stark contrast, the Sb(v)-based polymeric donor P3 exhibited pronounced chemical shift perturbations upon titration with acceptor polymer P4 (Fig. S5 in the SI). Incremental addition of P4 induced progressive upfield shifts in the diagnostic P3 proton signals (H^1^ and H^2^), consistent with specific intermolecular interactions (Fig. 3b). In addition, ^1^H NMR titration experiments were conducted for the PnB monomer 3 with the Lewis base monomer M2, revealing a similar trend in proton chemical shift changes (Fig. S9 in the SI). Nonlinear regression analysis based on a 1 : 1 binding model afforded binding constants of *K*_(__P3__/__P4__)_ = 1.57 × 10^4^ M^−1^ and *K*_(__3__/__M2__)_ = 0.69 × 10^4^ M^−1^. The substantially larger binding constant observed for the P3/P4 system suggests that the presence of polymer chains promotes PnB donor–acceptor interactions, a phenomenon that has also been observed in XB polymer systems.^23^

To further assess the relative strengths of PnB interactions in the P1/P4, P2/P4, and P3/P4 systems, the more sensitive ^13^C NMR technique was employed to monitor intermolecular interactions between the polymers. Equimolar mixtures of the polymeric acceptor P4 with the polymeric donors P1–P3 were subjected to comparative ^13^C NMR analyses. As a result, pronounced upfield shifts of the pyridine C_2_ carbon resonance were observed for the P2/P4 and P3/P4 systems, amounting to 0.52 and 4.33 ppm, respectively, whereas only negligible changes were detected for the P1/P4 system (Fig. 3c). These results clearly establish an interaction strength hierarchy of P3/P4 > P2/P4 >> P1/P4. Notably, no discernible chemical shift changes were detected for the ester carbonyl signals in any of the three systems, indicating that the pyridine moiety of P4 preferentially participates in PnB formation rather than the ester carbonyl group. This observation is fully consistent with the original molecular design.

**Fig. 4:**
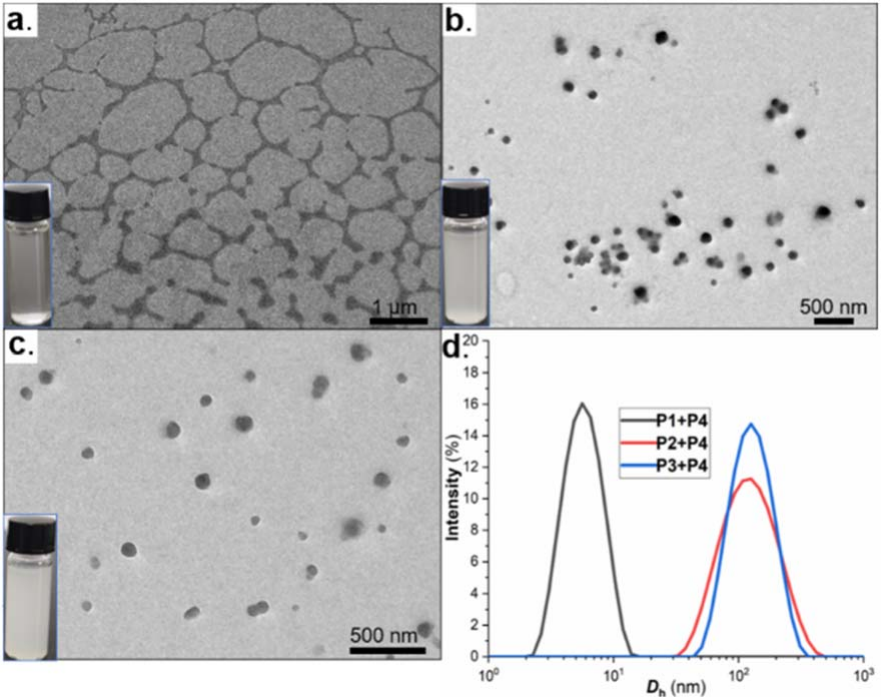
(a–c) TEM images of the P1/P4, P2/P4, and P3/P4 assemblies. (d) Size distribution histogram of the three systems.

Having established a PnB strength hierarchy of P3/P4 > P2/P4 >> P1/P4, we next examined the self-assembly behavior of these systems in solution. Owing to the hydrophobic nature of the polymers, assembly was induced by the gradual addition of water to THF solutions containing equimolar amounts of the PnB donor and acceptor components. Notably, distinct assembly behaviors were observed. Whereas the P2/P4 and P3/P4 systems formed translucent, gel-like solutions, the P1/P4 mixture remained a clear solution (Fig. 4a–c). Transmission electron microscopy (TEM) further revealed well-defined spherical nanostructures in the P2/P4 and P3/P4 systems, while no discernible assemblies were detected for P1/P4 (Fig. 4a–c). Consistent with these observations, dynamic light scattering (DLS) measurements revealed that the P2/P4 and P3/P4 systems formed nanoscale aggregates with average apparent hydrodynamic diameters of 111 nm (*σ* = 52 nm) and 118 nm (*σ* = 44 nm), respectively, whereas the P1/P4 system displayed a much smaller hydrodynamic diameter of only 5.3 nm (*σ* = 1.7 nm), indicative of the absence of significant aggregation (Fig. 4d). Collectively, these results indicate that pronounced PnB interactions in the P2/P4 and P3/P4 systems effectively promote supramolecular organization, whereas the absence of significant PnB interactions in the P1/P4 system is insufficient to drive self-assembly.

Having clarified the solution-state binding behavior of the polymeric donor–acceptor systems, we turned our attention to elucidating the relationship between PnB-PNs and the properties of dynamic materials. Polymer networks P^III^-PNs (P1/P4), Sb^III^-PNs (P2/P4), and Sb^V^-PNs (P3/P4) were fabricated *via* solvent evaporation from CHCl_3_ solutions containing equimolar amounts of donor and acceptor units (Table 1). To examine chain-length effects, an additional Sb(iii)-based network (Sb^III^-PNs′) was fabricated using P2 and a longer acceptor polymer P4′ with an *M*_n_ approximately 2.3 times that of P4. Furthermore, a hybrid network (Sb^V/III^-PNs) incorporating Sb(v) and Sb(iii) donors in a 1 : 1 ratio was constructed to specifically assess the influence of high-valent Sb centers on network dynamics. For comparison, halogen-bonded (XB-PNs) and hydrogen-bonded (HB-PNs) polymer networks were prepared under analogous conditions.

**Table 1:** Composition of the PNs and its corresponding glass transition temperature (*T*_g_)

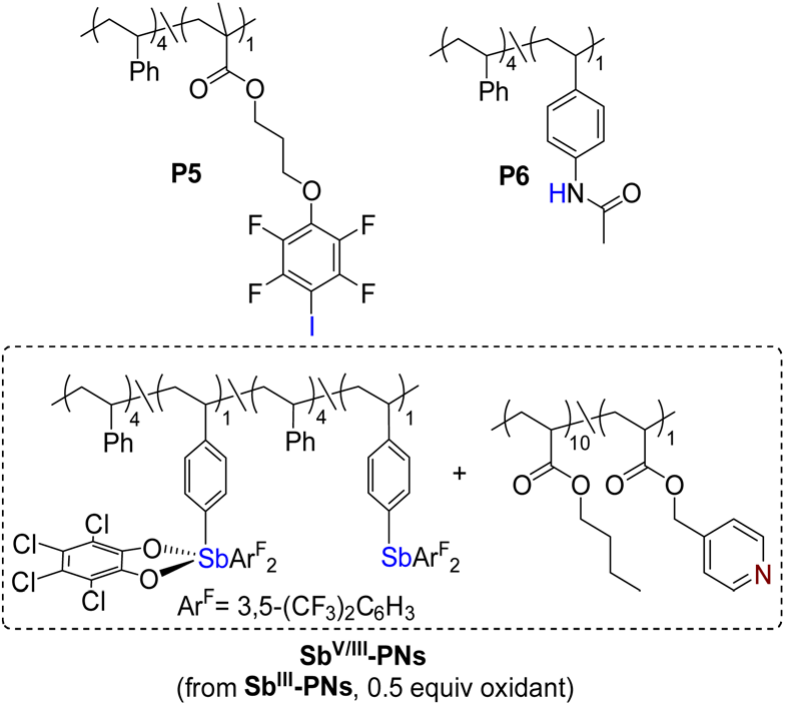				
Entry^a^	PNs	Donors	Acceptors	*T*_g_ [^o^C]
1	P^III^-PNs	P1	P4	−33
2	Sb^III^-PNs	P2	P4	−19
3	Sb^III^-PNs′	P2	P4′	−12
4	Sb^V/III^-PNs	—	—	−14
5	Sb^V^-PNs	P3	P4	9
6	XB-PNs	P5	P4	−35
7	HB-PNs	P6	P4	−37

a See the SI for details.

Differential scanning calorimetry (DSC) revealed a clear correlation between PnB strength and thermal properties. The Sb(iii)-based network Sb^III^-PNs exhibited a higher glass transition temperature (*T*_g_ = −19 °C) than the P(iii)-based P^III^-PNs (*T*_g_ = −33 °C) (Fig. S19 and S20), consistent with stronger Sb⋯N interactions imposing greater constraints on polymer chain mobility. Increasing the acceptor chain length further elevated the *T*_g_ of Sb^III^-PNs′ to −12 °C, likely due to enhanced chain entanglement. Notably, incorporation of Sb(v)-based PnB interactions led to a further increase in *T*_g_, with values of −14 °C for Sb^V/III^-PNs and 9 °C for Sb^V^-PNs, reflecting the superior bonding capability of Sb(v) and the resulting enhancement in network rigidity. In contrast, XB-PNs and HB-PNs displayed substantially lower *T*_g_ values (−35 °C and −37 °C, respectively), indicating their comparatively limited topological stability.

**Fig. 5:**
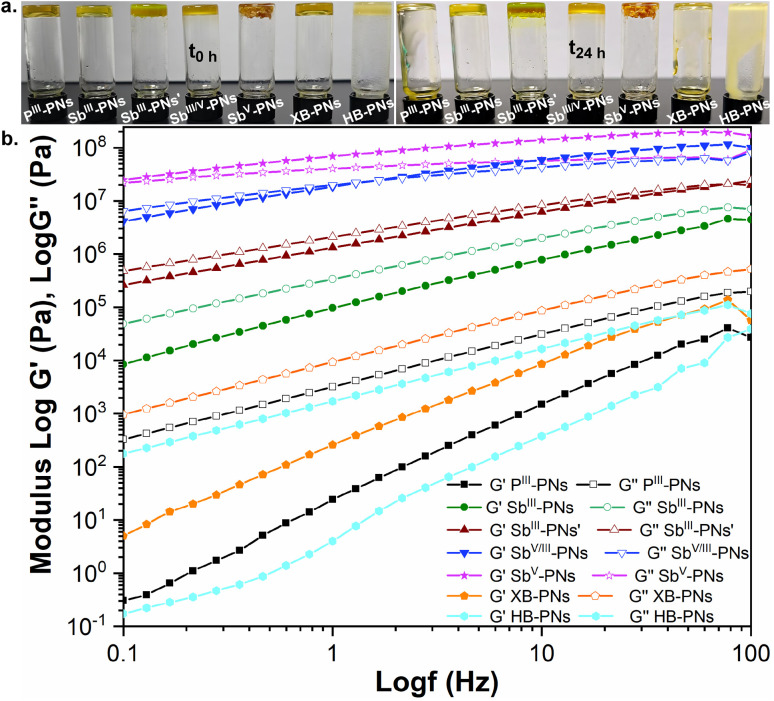
(a) Vial creep experiments. (b) Rheological experiments.

To further explore the mechanical implications of PnB, we conducted qualitative vial creep experiments to assess the dimensional stability of these PNs (Fig. 5a). Notably, Sb^III^-PNs′, Sb^V^-PNs and Sb^V/III^-PNs exhibited excellent shape retention, with no visible deformation under the applied conditions. In comparison, Sb^III^-PNs exhibited only minor distortion, whereas P^III^-PNs, XB-PNs, and HB-PNs all underwent pronounced creep deformation.

Complementary oscillatory shear rheological measurements were conducted to elucidate the influence of PnB on the viscoelastic properties of crosslinked polymer networks (Fig. 5b). At room temperature, both P^III^-PNs and Sb^III^-PNs exhibited loss moduli (*G*″) exceeding their storage moduli (*G*′), characteristic of viscoelastic liquid-like behavior. Compared to P^III^-PNs, Sb^III^-PNs displayed substantially higher moduli and a smaller *G*″–G′ gap, indicating enhanced structural rigidity arising from stronger crosslinking interactions. Incorporation of longer acceptor polymer chains in Sb^III^-PNs′ further increased the moduli and narrowed the *G*″–*G*′ gap. The introduction of Sb(v)-based PnB led to a further enhancement in mechanical strength: Sb^V/III^-PNs and Sb^V^-PNs exhibited progressively higher moduli, with Sb^V^-PNs displaying *G*′ > *G*″, indicative of dominant elastic behavior. In contrast, XB-PNs and HB-PNs showed rheological responses comparable to those of P^III^-PNs.

To quantitatively correlate network mechanics with the bond strength of PnB, creep-recovery experiments were performed under constant stress (1000 Pa, 1000 s) (Table 2, Fig. S31–S39 in the SI). P^III^-PNs, XB-PNs, and HB-PNs exhibited large irreversible strains of 202 700%, 67 020%, and 375 500%, respectively, indicative of weak network cohesion and poor mechanical robustness. By comparison, Sb^III^-PNs displayed a substantially reduced deformation of 1856%, reflecting enhanced structural integrity. Notably, extension of the acceptor polymer chains further suppressed creep deformation, as evidenced by a markedly lower strain of 84.16% for Sb^III^-PNs′. In sharp contrast, networks incorporating stronger Sb(v)–PnB interactions exhibited dramatically improved resistance to creep, with strains of only 1.72% for the Sb(iii)/Sb(v) hybrid Sb^V/III^-PNs and an exceptionally low 0.11% for the pure Sb(v)-based Sb^V^-PNs. Moreover, Sb^V^-PNs maintained outstanding thermomechanical robustness at elevated temperature (60 °C), showing only 8.56% strain under identical testing conditions. Upon the addition of one equivalent of quinoline to Sb^V^-PNs, the room-temperature creep strain increased from 0.11% to 18.74%, suggesting that quinoline acts as a competitive reagent that partially disrupts Sb(v)–PnB interactions and thereby weakens the polymer network.

**Table 2:** Strains of PNs under constant stress (1000 Pa, 1000 s)

Entry^a^	PNs	Temperature [^o^C]	Strain [%]
1	P^III^-PNs	25	202 700
2	Sb^III^-PNs	25	1856
3	Sb^III^-PNs′	25	84.16
4	Sb^V/III^-PNs	25	1.72
5	Sb^V^-PNs	25	0.11
6	Sb^V^-PNs	60	8.56
7	Sb^V^-PNs + quinoline	25	18.74
8	XB-PNs	25	67 020
9	HB-PNs	25	375 500

a See the SI for details.

**Fig. 6:**
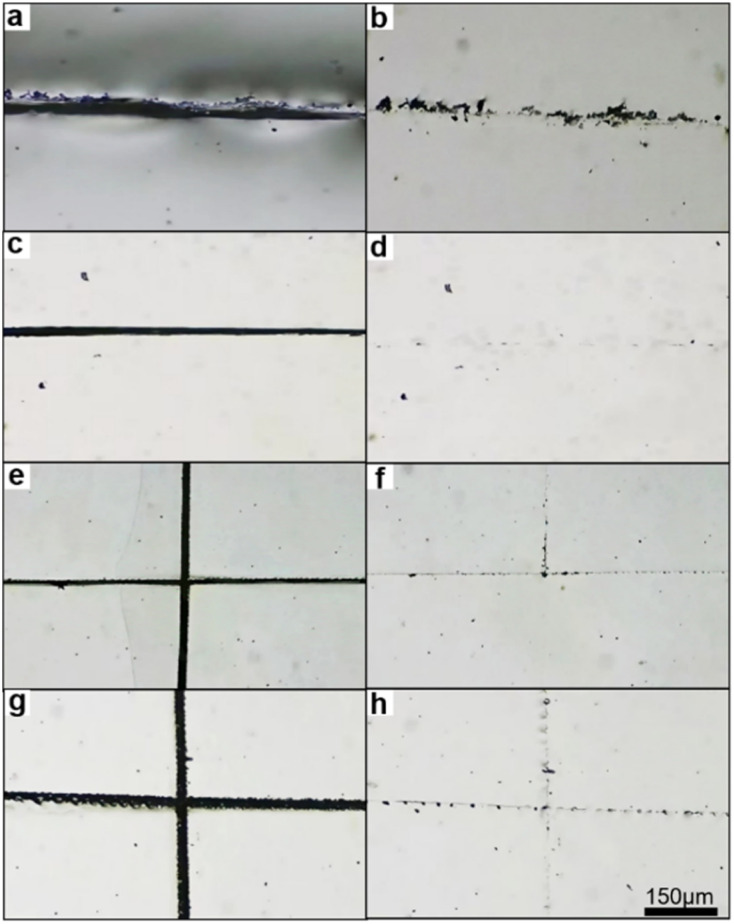
(a) Optical micrographs of the damaged film Sb^III^-PNs. (b) Optical micrographs of the film Sb^III^-PNs after healing for 5 h at room temperature. (c) Optical micrographs of the damaged film Sb^III^-PNs′. (d) Optical micrographs of the film Sb^III^-PNs′ after healing for 12 h at room temperature. (e) Optical micrographs of the damaged film Sb^V/III^-PNs. (f) Optical micrographs of the film Sb^V/III^-PNs after healing for 20 h at room temperature. (g) Optical micrographs of the damaged film Sb^V^-PNs. (h) Optical micrographs of the film Sb^V^-PNs after healing for 24 h at 60 °C.

Motivated by the pronounced mechanical reinforcement conferred by Sb-based PnB in dynamic materials, films of Sb^III^-PNs, Sb^III^-PNs′, Sb^V/III^-PNs and Sb^V^-PNs were prepared to systematically investigate the influence of PnB on self-healing behavior in dynamic polymer networks. Notably, the Sb(iii)-based networks Sb^III^-PNs and Sb^III^-PNs′ exhibited excellent room-temperature self-healing, achieving complete notch closure within 5 h and 12 h, respectively (Fig. 6a–d). In contrast, the stronger Sb(v)–PnB interactions reduced healing efficiency, since the Sb(v)/Sb(iii) hybrid Sb^V/III^-PNs required 20 h at room temperature to reach a similar degree of healing (Fig. 6e–f). Remarkably, Sb^V^-PNs exhibited effective healing only at elevated temperature (60 °C) (Fig. 6g–h). This inverse relationship between healing kinetics and pnictogen-bonding strength is attributed to the restricted chain mobility imposed by stronger PnB interactions at damaged interfaces, which hinders effective contact and reorganization of donor and acceptor moieties. As a result, additional thermal energy is necessary to overcome activation barriers associated with chain diffusion and dynamic bond exchange.

Given that Sb(v)–PnB in polymer networks exhibit markedly stronger constraints on chain mobility than their Sb(iii) counterparts, a quantitative analysis of the exchange kinetics of these two dynamic bonds is essential. Such polymer networks display frequency-dependent mechanical behavior characterized by a single crossover frequency separating the elastic-dominated and viscous-dominated regimes. This crossover frequency corresponds to the inverse of the terminal relaxation time (*τ*), which represents the average timescale for network reconfiguration and can be regarded as the effective bond lifetime.^24^ The shear modulus spectra of Sb^III^-PNs and Sb^V^-PNs were measured at 20 °C, affording terminal relaxation times (*τ*) of 3.72 ms and 45.15 s, respectively (Fig. S40–S41 in the SI). This more than four-orders-of-magnitude increase in *τ* directly evidences a dramatically slower network reconfiguration and severely restricted chain mobility in the Sb(v)-based networks. To further elucidate the kinetic origin of this disparity, temperature-dependent shear modulus measurements were performed to track the evolution of *τ* and to extract the corresponding relaxation activation energies (*E*_a_).^25^Sb^V^-PNs exhibit a substantially higher *E*_a_ than Sb^III^-PNs (240 kJ mol^−1^*versus* 166 kJ mol^−1^), indicating a much larger energetic barrier for chain diffusion and dynamic bond exchange. The unusually large activation energy of Sb^V^-PNs implies a pronounced temperature sensitivity of chain dynamics, thereby rationalizing the necessity of elevated temperatures to enable efficient self-healing.

**Fig. 7:**
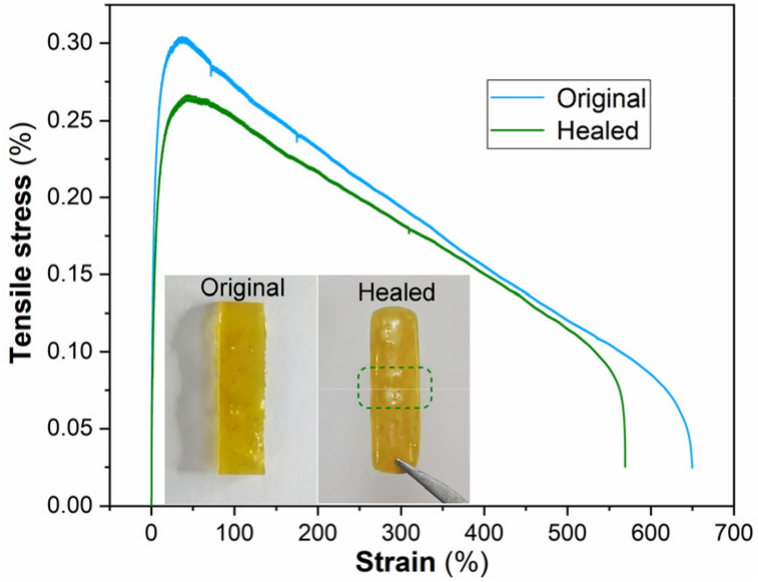
The stress–strain curves of Sb^V/III^-PNs before damage and after healing for 24 hours at room temperature.

Building on the established relationship between pnictogen-bonding strength and healing efficiency, tensile testing was conducted on Sb^V/III^-PNs, selected for its optimal balance of room temperature self-healing and mechanical performance (Fig. 7). The pristine specimen exhibited an ultimate tensile strength of 0.30 MPa and an elongation at break of 651%. Following complete sectioning to mimic material damage and subsequent intimate contact of the fracture surfaces, the sample was allowed to heal at room temperature for 24 hours. Post-healing tensile measurements demonstrated 90% recovery of the original strength (0.27 MPa) and 87% restoration of elongation at break (569%), confirming substantial mechanical recovery. This recovery correlates directly with the observed macroscopic healing of Sb^V/III^-PNs films, confirming effective damage remediation under mild conditions.

Given that PnB constitutes a weak interaction with low sensitivity to polar solvents, this feature was expected to endow PnB-PNs with self-healing ability in aqueous environments. To verify this hypothesis, samples of Sb^V/III^-PNs were completely cut, the freshly exposed surfaces were brought into intimate contact, and the specimens were immersed in water for 24 h at room temperature. Subsequent tensile tests under identical conditions to the pristine samples revealed that the mechanical parameters of specimens self-healed in water were comparable to those healed under ambient conditions (Fig. S47 in the SI). These findings confirm that the self-healing behavior of Sb^V/III^-PNs-based dynamic materials is insensitive to aqueous environments.

**Fig. 8:**
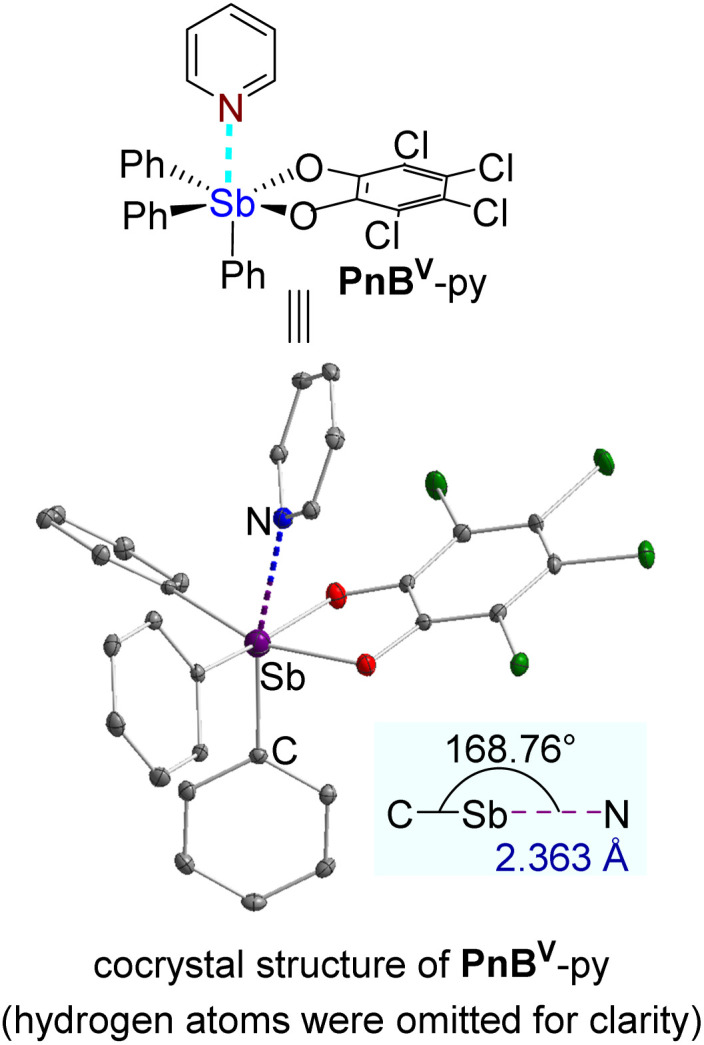
The cocrystal structure of PnB^V^-py.

Motivated by the robust polymer networks afforded by Sb(v)-based dynamic materials through strong PnB, we investigated the bonding modes between PnB donors and acceptors. The cocrystal of Sb(v)-based derivative PnB^V^ with pyridine was obtained *via* solvent diffusion using dichloromethane and *n*-hexane (Fig. 8). The crystal structure unambiguously reveals PnB interactions between the electrophilic antimony center in PnB^V^ and the nitrogen atom of pyridine. Key geometric parameters provide conclusive evidence: the Sb⋯N distance of 2.363 Å is markedly shorter than the sum of their van der Waals radii (3.65 Å), while the nearly linear C–Sb⋯N angle of 168.76° is characteristic of directional PnB. Furthermore, the Sb⋯N separation significantly exceeds typical covalent Sb–N bond lengths (∼2.11 Å), thereby excluding the formation of coordinate covalent bonds. These structural data definitively validate the designed PnB interaction between Sb(v)-donor polymers and pyridine-functionalized acceptors.

## Conclusions

In summary, polymer networks based on PnB have been established as a new platform for tuning the properties of dynamic materials. Several distinct crosslinked polymers featuring pnictogen-bonding motifs were synthesized, with ^1^H NMR titration providing initial evidence of supramolecular interactions between polymeric PnB donors and acceptors. Notably, strengthening PnB within these polymer networks markedly enhances the topological stability, whereas comparable hydrogen-bonded systems exhibit negligible improvements. Moreover, Sb-based PnB-PNs exhibit tunable self-healing behavior, enabling on-demand switching between autonomous repair at ambient temperature and thermally triggered healing. Remarkably, the healing process can even be accomplished in aqueous environments. The complexation mode between polymeric donors and acceptors was further corroborated by cocrystal structures of model compounds. These findings highlight the significant potential of PnB-PNs for the design and development of next-generation dynamic materials.

## Author contributions

W. W. and Y. W. conceived and designed the experiments. Q. S. conducted the experiments and prepared the supplementary materials. Y. L. performed DFT calculations. W. W. wrote the manuscript. All authors analysed the data, discussed the results and approved the manuscript.

## Conflicts of interest

There are no conflicts to declare.

## Supplementary Material

SC-017-D6SC00802J-s001

SC-017-D6SC00802J-s002

## Data Availability

CCDC 2480747 contains the supplementary crystallographic data for this paper.^26^ The data supporting this article have been included as part of the supplementary information (SI). Supplementary information: experimental details, spectroscopic data for the compounds, X-ray crystallographic details, and theoretical calculation details. See DOI: https://doi.org/10.1039/d6sc00802j.
