# Supplementary material for: Pnictogen-bonding-crosslinked polymer networks: constructing self-healing materials
Source: Chem Sci. 2026 Feb 27;17(16):7958–66. doi: 10.1039/d6sc00802j (PMC12947258; doi:10.1039/d6sc00802j)
Supplement: SC-017-D6SC00802J-s001 [file SC-017-D6SC00802J-s001.pdf]

Supplementary Information (SI) for Chemical Science.
This journal is © The Royal Society of Chemistry 2026

Supporting Information for

Pnictogen-bonding-crosslinked polymer networks: constructing self-healing materials

Qingli Song,[a] Yi Liu,[b] Yao Wang*[b] and Wei Wang*[a,c]

[a] Department of Chemistry, Zhengzhou University, Zhengzhou 450001, China

[b] School of Chemistry and Chemical Engineering, Shandong University, Jinan 250100, China

[c] Shenzhen Research Institute of Shandong University, A301 Virtual University Park in South District of Shenzhen

*Corresponding author. Email: wangwei_chem@zzu.edu.cn; yaowang@sdu.edu.cn

Table of contents

1. Materials and instrumentations 2
2. Preparation of polymers 3
3. The interaction between donor polymers and acceptor polymers 14
4. Self assembly studies 21
5. Thermogravimetric analysis (TGA) 22
6. Differential scanning calorimetry (DSC) 26
7. Rheology 32
8. Self-healing 39
9. Tensile analysis 41
10. The cultivation of cocrystal (PnB^{V}-py) 42
11. X-ray crystallographic data 43
12. Computational details 44
13. Copies of NMR spectra and GPC spectra 49
14. Reference 77

1. Materials and instrumentations

All the chemicals were either purchased from commercial suppliers or purified by standard procedures as specified in *Purification of Laboratory Chemicals*, 7th Ed (Armarego, W. L. F.; Chai, C. L. L. Butterworth Heinemann: 2013). All reactions were carried out under nitrogen atmosphere. Analytical thin-layer chromatography (TLC) was performed on silica gel plates and analyzed by UV light or by potassium permanganate stains followed by heating. Flash chromatography was carried out utilizing silica gel (200-300 mesh). ^{1}H NMR, ^{13}C NMR, ^{19}F NMR, ^{31}P NMR spectra were recorded in $CDCl_3$ or CD_2Cl_2 at room temperature on a Bruker AM-400 spectrometer (400 MHz ^{1}H, 100 MHz ^{13}C, 376 MHz ^{19}F, 162 MHz ^{31}P). The chemical shifts are reported in ppm relative to either the residual solvent peak (^{13}C) (δ = 77.00 ppm for $CDCl_3$, δ = 53.84 ppm for CD_2Cl_2), (^{1}H) (δ = 7.26 ppm for $CDCl_3$; δ = 5.32 ppm for CD_2Cl_2). Data for ^{1}H NMR are reported as follows: chemical shift (δ ppm), multiplicity (s = singlet, d = doublet, t = triplet, q = quartet, m = multiplet, dd = doublet doublet), coupling constant (Hz), integration. Data for ^{13}C NMR, ^{19}F NMR and ^{31}P NMR are reported as chemical shift. Elemental analyses were conducted on a Perkin-Elmer 2400CHN elemental analyzer. Molecular weights of polymers were determined by GPC using an Agilent 1260 Infinity II system equipped with a refractive index detector (DMF with 0.1% LiBr, 45 °C, 1.0 mL/min). TEM imaging was performed on a JEOL JEM-2100Plus microscope at 200 kV. Thermogravimetric analysis (TGA) employed a PerkinElmer STA 8000 under N_2 atmosphere. DSC measurements utilized a TA Q2000 calorimeter. Rheological properties were characterized on a Thermo Scientific HAAKE MARS 60 rheometer. Tensile testing was performed on an Instron 3343 Universal Testing System according to ASTM D1708 standard.

2. Preparation of polymers

Procedure for preparation of **1**:

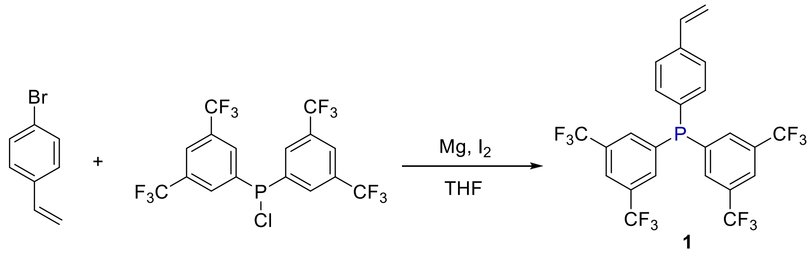

Magnesium turnings (3.8 mmol) were placed in a flame-dried two-necked flask equipped with a reflux condenser under a nitrogen atmosphere. Anhydrous THF (2.0 mL) and a catalytic crystal of I_2 were added. A solution of *p*-bromostyrene (3.1 mmol) in anhydrous THF (1.0 mL) was added dropwise to the flask. The reaction mixture was subsequently heated under reflux at 65 °C for 2 h. After cooling to room temperature, the resulting Grignard solution was further cooled to 0 °C. A solution of bis(3,5-di(trifluoromethyl)phenyl)chlorophosphane (2.0 mmol) in anhydrous THF (3.0 mL) was added dropwise under vigorous stirring. The reaction mixture was stirred overnight at room temperature. The reaction was quenched by slow addition of saturated aqueous NH_4Cl (5 drops), diluted with THF (20.0 mL), and extracted with EtOAc. The combined organic extracts were dried over Na_2SO_4, filtered, and concentrated in vacuo. The residue was purified by flash chromatography on silica gel (eluent: petroleum ether) to afford the product as a colorless oil (840.48 mg, 75%).

1: ^{1}H NMR (400 MHz, $CDCl_3$): δ 7.90 (s, 2H), 7.71 (dd, *J* = 6.6, 1.6 Hz, 4H), 7.50–7.48 (m, 2H), 7.32–7.28 (m, 2H), 6.74 (dd, *J* = 17.6, 10.9 Hz, 1H), 5.85 (dd, *J* = 17.6, 0.7 Hz, 1H), 5.38 (d, *J* = 11.0 Hz, 1H); ^{13}C NMR (100 MHz, $CDCl_3$): δ 139.93 (d, *J* = 12.1 Hz), 139.69, 135.77, 134.29 (d, *J* = 21.5 Hz), 133.16–132.92 (m), 132.28 (dq, *J* = 6.2, 33.3 Hz), 131.70 (d, *J* = 9.9 Hz), 127.30 (d, *J* = 8.1 Hz), 123.42–123.28 (m), 122.97 (q, *J* = 271.6 Hz), 116.27; ^{19}F NMR (376 MHz, $CDCl_3$): δ -63.00; ^{31}P NMR (162 MHz, $CDCl_3$): δ -4.53; EI-MS, m/z, (M^+) calcd for $C_{24}H_{13}F_{12}P$: 560.1. Found: 560.3.

Procedure for preparation of **2**:

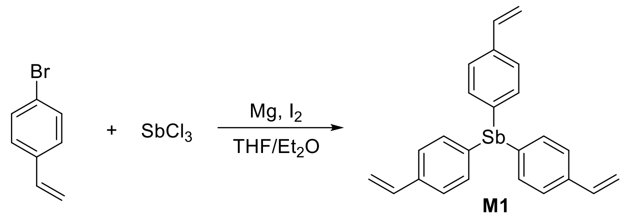

M1

Magnesium turnings (148.8 mmol) were placed in a flame-dried two-necked flask equipped with a reflux condenser under a nitrogen atmosphere. Anhydrous THF (72.0 mL) and a crystal of I_2 were added. A solution of *p*-bromostyrene (120.0 mmol) in anhydrous THF (36.0 mL) was added dropwise to the flask. The mixture was heated under reflux at 65 °C for 2 h. After cooling to room temperature, the resulting Grignard solution was cooled to 0 °C. A solution of $SbCl_3$ (40.5 mmol) in anhydrous Et_2O (40.0 mL) was added dropwise over approximately 30 min with stirring. The reaction mixture was then stirred overnight at room temperature. The mixture was carefully treated with a saturated aqueous NH_4Cl (0.5 mL), diluted with THF (150.0 mL), and extracted with EtOAc. The combined organic extracts were dried over Na_2SO_4, treated with activated carbon, and filtered through a pad of Celite. The volatiles were removed under reduced pressure. The residue was redissolved in DCM (50.0 mL), filtered through a short silica gel column (3 cm), and eluted with *n*-hexane (800.0 mL). Concentration of the eluate under reduced pressure afforded a white solid (10.52 g, 61%).

M1: ^{1}H NMR (400 MHz, $CDCl_3$): δ 7.41–7.35 (m, 4H), 6.70 (dd, *J* = 17.6, 10.9 Hz, 1H), 5.76 (dd, *J* = 17.6, 0.9 Hz, 1H), 5.25 (dd, *J* = 10.9, 0.9 Hz, 1H); ^{13}C NMR (100 MHz, $CDCl_3$): δ 138.02, 137.86, 136.63, 136.40, 126.62, 114.41; Anal. Calcd for $C_{24}H_{21}Sb$: C (66.86%), H (4.91%). Found: C (66.60%), H (4.77%).

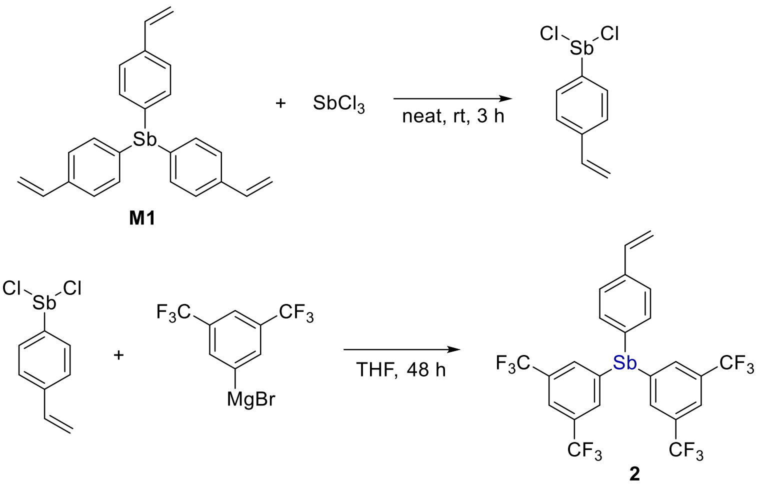

$SbCl_3$ (4.8 mmol) and **M1** (2.4 mmol) were stirred together solvent-free at room temperature for 3 h. The resulting solid was dissolved in anhydrous THF (8.0 mL) and was added dropwise to the Grignard solution (15.0 mmol) at 0 °C. The rection mixture was stirred at room temperature for 48 h, then quenched with saturated aqueous NH_4Cl (5 drops). The mixture was dried over anhydrous Na_2SO_4 and concentrated. The residue was purified by flash chromatography on silica gel (eluent: petroleum ether) to afford the product as a colorless oil (2.02 g, 43%).

2: ^{1}H NMR (400 MHz, $CDCl_3$): δ 7.89–7.87 (m, 6H), 7.47 (d, *J* = 8.1 Hz, 2H), 7.37 (d, *J* = 8.1 Hz, 2H), 6.72 (dd, *J* = 17.6, 10.9 Hz, 1H), 5.83 (dd, *J* = 17.6, 0.8 Hz, 1H), 5.34 (dd, *J* = 10.9, 0.8 Hz, 1H); ^{13}C NMR (100 MHz, $CDCl_3$): δ 140.52, 139.39, 136.15, 136.07, 135.74 (br s), 135.22, 132.05 (q, *J* = 33.0), 127.51, 123.14 (q, *J* = 271.6), 123.36–123.21 (m), 115.70; ^{19}F NMR (376 MHz, $CDCl_3$): δ -62.99; Anal. Calcd for $C_{24}H_{13}F_{12}Sb$: C (44.27%), H (2.01%). Found: C (44.14%), H (2.06%).

Procedure for preparation of **3**:

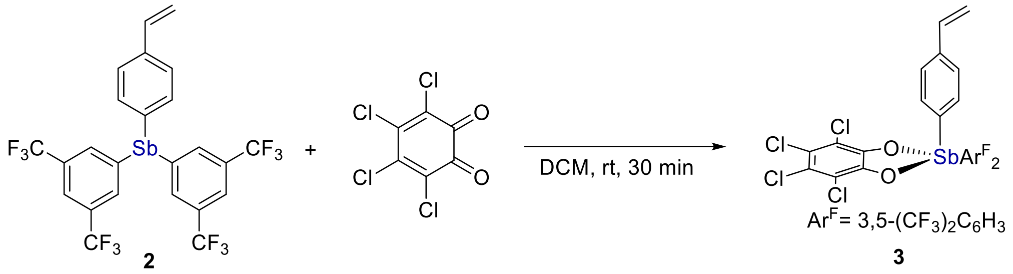

Reactants **2** (1.2 mmol) and 3,4,5,6-tetrachlorocyclohexa-3,5-diene-1,2-dione (1.2 mmol) were added to DCM (3.0 mL) under nitrogen atmosphere. The above reaction mixture was stirred at room temperature for 30 min, concentrated under reduced pressure to approximately 1 mL and added *n*-hexane (5.0 mL) slowly. The two-phase solution was then placed for 2 h at room temperature to afford yellow solid. The precipitate was filtered off and washed by *n*-pentane to afford product (699.64 mg, 65%).

3: ^{1}H NMR (400 MHz, $CDCl_3$): δ 8.29 (s, 4H), 8.13 (s, 2H), 7.70 (d, J = 8.4 Hz, 2H), 7.63 (d, J = 8.2 Hz, 2H), 6.74 (dd, J = 17.6, 10.9 Hz, 1H), 5.91 (d, J = 17.7 Hz, 1H), 5.47 (d, J = 10.9 Hz, 1H); ^{13}C NMR (100 MHz, $CDCl_3$): δ 143.20, 143.02, 138.68, 135.27 (br s), 135.18, 134.89, 133.09 (q, J = 33.8), 130.19, 128.25, 126.28–126.13 (m), 122.68 (q, J = 271.6), 122.04, 118.29, 117.33; ^{19}F NMR (376 MHz, $CDCl_3$): δ -63.06; Anal. Calcd for $C_{30}H_{13}Cl_4F_{12}O_2Sb$: C (40.17%), H (1.46%). Found: C (40.02%), H (1.39%).

Procedure for preparation of **M2**:

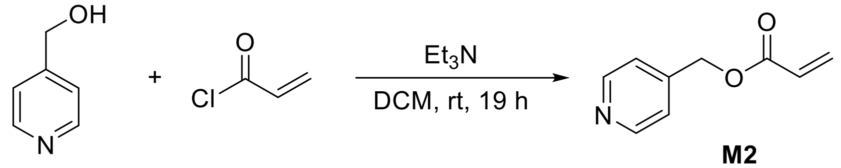

M2

A solution of 4-pyridinemethanol (0.08 mol) in 400.0 mL of DCM was cooled to 0 °C, and triethylamine (0.25 mol) was added slowly. Acryloyl chloride (0.17 mol) in 50.0 mL of DCM was then added dropwise over the course 60 min, while maintaining the bath temperature below 0 °C. The solution was warmed to room temperature, stirred for 19 h, and DCM was removed under reduced pressure. The residue was triturated with EtOAc (250.0 mL), filtered and washed with saturated aqueous $NaHCO_3$ (100.0 mL). The organic layer was dried over Na_2SO_4 and concentrated in vacuo. The residue was purified by flash chromatography on silica gel (eluent: petroleum ether/EtOAc = 2:3) to afford the product as fluorescent greenish colour oil (1.96 g, 15%).

M2: ^{1}H NMR (400 MHz, $CDCl_3$): δ 8.58 (d, J = 4.6 Hz, 2H), 7.24 (d, J = 5.1 Hz, 2H), 6.48 (d, J = 17.3 Hz, 1H), 6.19 (dd, J = 17.3, 10.4 Hz, 1H), 5.90 (d, J = 10.4 Hz, 1H), 5.19 (s, 2H); ^{13}C NMR (100 MHz, $CDCl_3$): δ 165.49, 149.94, 144.73, 131.76, 127.65, 121.75, 64.16. The above data of product **M2** is consistent with the reported references [1].

Procedure for preparation of **M4**:

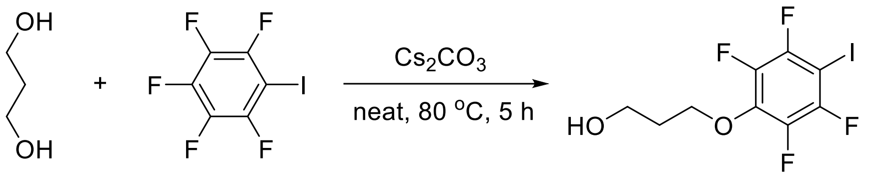

M3

Perfluoroiodobenzene (20.0 mmol) was combined with propanediol (174.0 mmol) and Cs_2CO_3 (20.1 mmol) in a reaction tube. The solution was heated to 80 °C and stirred for 5 h, then poured into 120.0 mL of water. The mixture was extracted with DCM (4 × 150.0 mL). The combined organic extracts were dried over Na_2SO_4 and concentrated. The residue was purified by flash chromatography on silica gel (eluent: petroleum ether/EtOAc = 5:1) to afford the product as a white solid (4.62 g, 66%)

M3: ^{1}H NMR (400 MHz, $CDCl_3$): δ 4.40 (t, J = 6.0 Hz, 2H), 3.89 (q, J = 5.8 Hz, 2H), 2.07–2.01 (m, 2H), 1.53–1.50 (m, 1H); ^{13}C NMR (100 MHz, $CDCl_3$): δ 148.61, 146.21, 142.05, 139.41, 138.30, 72.67, 63.96, 59.35, 32.58; ^{19}F NMR (376 MHz, $CDCl_3$): δ -121.19 to -121.23, -154.48 to -154.52. The above data of product **M3** is consistent with the reported references [2].

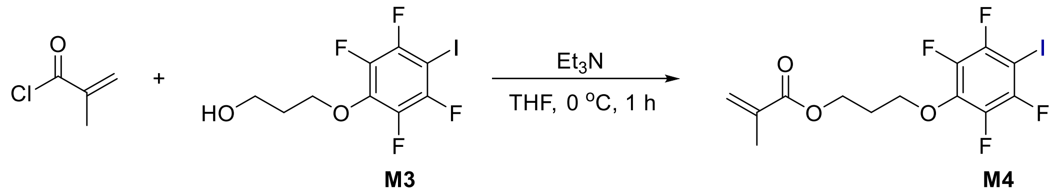

M3 **M4**

In a round-bottomed flask, **M3** (14.8 mmol) was dissolved in anhydrous THF (12.0 mL) and triethylamine (32.4 mmol). The solution was cooled to 0 °C and methacryloyl chloride (21.6 mmol) was added dropwise over 10 min. After stirring at 0 °C for 1 h, the reaction mixture was filtered using DCM (150.0 mL) as eluent. The filtrate was

concentrated and the residue was purified by flash column chromatography on silica gel (eluent: petroleum ether/acetone = 200:1) to afford the product as a colorless oil (3.28 g, 53%).

M4: ^{1}H NMR (400 MHz, $CDCl_3$): δ 6.10 (s, 1H), 5.58–5.56 (m, 1H), 4.38–4.32 (m, 4H), 2.20–2.14 (m, 2H), 1.94 (s, 3H); ^{13}C NMR (100 MHz, $CDCl_3$): δ 167.96, 148.96, 145.13, 141.92, 139.46,138.10, 136.17, 125.03, 71.68, 63.34, 60.32, 31.65, 18.94; ^{19}F NMR (376 MHz, $CDCl_3$): δ -121.12 to -121.19, -154.34 to -154.48. The above data of product **M4** is consistent with the reported references [2].

Procedure for preparation of **M5**:

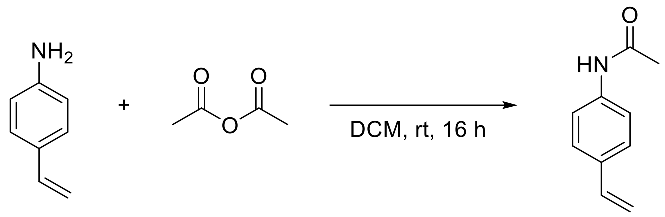

4-Vinylaniline (3.0 mmol) was added to an oven-dried round-bottom flask under an argon atmosphere via syringe. The flask was sealed with a rubber septum and purged with argon. Dry dichloromethane (7.5 mL, 0.4 M) was added, followed by acetic anhydride (3.6 mmol, 1.2 equiv). The reaction mixture was stirred at room temperature and monitored by TLC. After completion, the mixture was washed with saturated aqueous sodium carbonate solution. The organic layer was dried over Na_2SO_4, and the solvent was removed under reduced pressure. The crude product was purified by column chromatography on silica gel (eluent: hexane/ethyl acetate = 1:2) to afford the desired product as a white solid (440.1 mg, 91% yield).

M5: ^{1}H NMR (400 MHz, $CDCl_3$): δ 7.46 (d, J = 8.5 Hz, 2H), 7.36 (d, J = 8.5 Hz, 2H), 6.67 (dd, J = 17.6, 10.9 Hz, 1H), 5.72–5.62 (m, 1H), 5.19 (d, J = 10.9 Hz, 1H), 2.18 (s, 3H); ^{13}C NMR (100 MHz, $CDCl_3$): δ 168.16, 137.39, 136.09, 133.74, 126.83, 119.73, 113.03, 24.64. The above data of product **M5** is consistent with the reported references [3].

Procedure for preparation of polymer **P1** or **P2**:

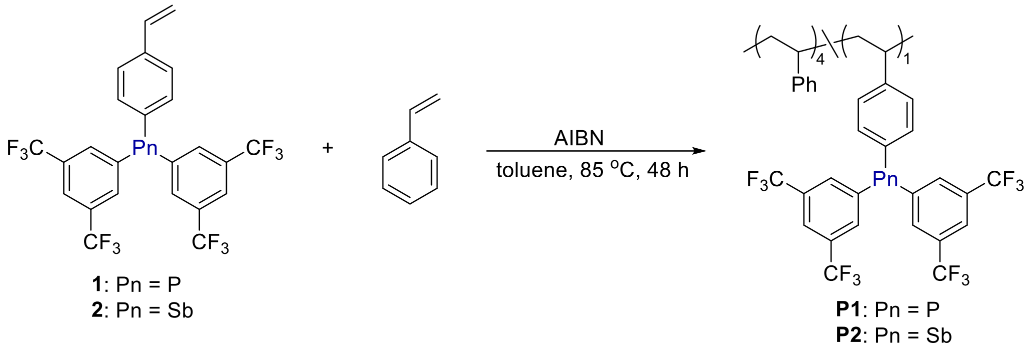

To a reaction mixture of **1** or **2** (4.1 mmol), styrene (16.4 mmol) and AIBN (0.25 mmol) in a 50 mL Schlenk tube was added anhydrous toluene (20.0 mL). The mixture was deoxygenated by nitrogen purging for 15 min. Then, the reaction mixture was stirred at 85 °C for 24 h under a nitrogen atmosphere. An additional portion of AIBN (0.25 mmol) was added, and the polymerization was allowed to proceed with stirring at 85 °C for a further 24 h. The solvent was removed using rotatory evaporation. The residue was washed with cold *n*-pentane and methanol, and dried in a vacuum oven at 50 °C to afford the target polymer. Note: **P1** requires nitrogen storage due to air sensitivity.

P1: ^{1}H NMR (400 MHz, CD_2Cl_2): δ 8.16–8.07 (m, 6H), 7.05–6.47 (m, 24H), 1.81–1.28 (m, 16H); ^{19}F NMR (376 MHz, $CDCl_3$): δ -62.96; ^{31}P NMR (162 MHz, $CDCl_3$): δ -4.06; Anal. (found): C (68.59), H (4.71); GPC (DMF, PS-standard): M_n = 13,900 g/mol; M_w = 26,100 g/mol; $Đ$ = 1.88.

P2: ^{1}H NMR (400 MHz, CD_2Cl_2): δ 7.87 (s, 6H), 7.03–6.57 (m, 24H), 1.99–1.27 (m, 16H); ^{19}F NMR (376 MHz, $CDCl_3$): δ -62.93; Anal. (found): C (62.21), H (4.38); GPC (DMF, PS-standard): M_n = 12,300 g/mol; M_w = 24,400 g/mol; $Đ$ = 1.98.

Procedure for preparation of polymer **P3**:

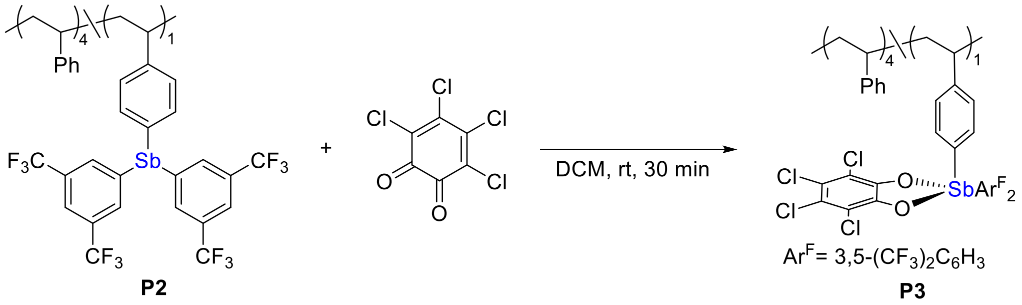

Reactants **P2** (1.2 mmol) and 3,4,5,6-tetrachlorocyclohexa-3,5-diene-1,2-dione (1.2 mmol) were added to DCM (10.0 mL) under nitrogen atmosphere. The above reaction mixture was stirred at room temperature for 30 min and concentrated under reduced pressure. The resulting solid was dissolved in a cold methanol/*n*-pentane (v/v = 1:1) mixed solvent system (20.0 mL), the solution was concentrated to approximately 10 mL. The solid was collected by filtration, washed with cold *n*-pentane, and dried in a vacuum oven at 50 °C to afford the target polymer.

P3: ^{1}H NMR (400 MHz, CD_2Cl_2): δ 8.27–8.16 (m, 6H), 7.30–6.54 (m, 24H), 1.57–1.27 (m, 16H); ^{19}F NMR (376 MHz, $CDCl_3$): δ -63.05; Anal. (found): C (56.38), H (3.67); GPC (DMF, PS-standard): M_n = 14,400 g/mol; M_w = 27,700 g/mol; $Đ$ = 1.92.

Table S1. The molar ratio of copolymers be specified by ^{1}H NMR.

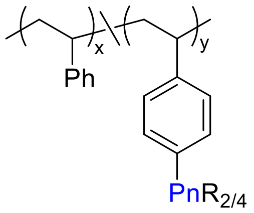

copolymer	x/y
**P1**	3.8/1.0
**P2**	4.1/1.0
**P3**	4.1/1.0

Procedure for preparation of polymer **P4**:

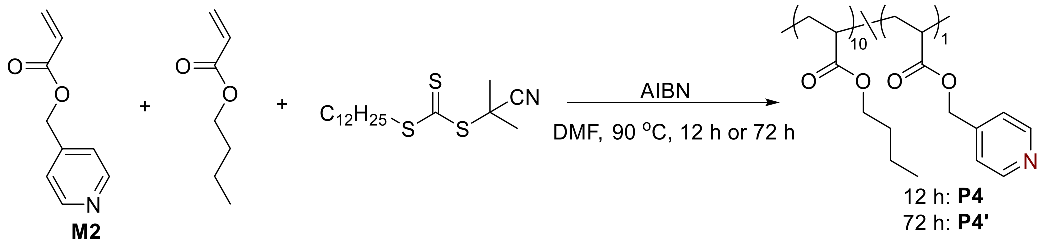

To a reaction mixture of **M2** (3.1 mmol), butyl acrylate (31.0 mmol), AIBN and 2-cyano-2-propyl dodecyl trithiocarbonate (CTA) in a 50 mL Schlenk tube was added anhydrous DMF (20.0 mL). The ratio of [CTA] to [AIBN] was always 5/1 and the ratio of [**M2** + butyl acrylate] to [CTA] was 500/1. Above mixture was deoxygenated by

nitrogen purging for 15 min and stirred at 90 °C for 12 h or 72 h. The polymers were purified by precipitation in cold water and methanol, respectively.

P4: ^{1}H NMR (400 MHz, $CDCl_3$): δ 8.60–8.59 (m, 2H), 7.28 (s, 2H), 5.09–5.05 (m, 2H), 4.04–3.99 (m, 20H), 2.28–0.85 (m, 119H); Anal. (found): C (66.32), H (9.09), N (0.91); GPC (DMF, PS-standard): M_n = 11,500 g/mol; M_w = 34,200 g/mol; $Đ$ = 2.97.

P4': ^{1}H NMR (400 MHz, $CDCl_3$): δ 8.60–8.58 (m, 2H), 7.26 (s, 2H), 5.21–5.02 (m, 2H), 4.05–3.98 (m, 20H), 2.31–0.90 (m, 113H); Anal. (found): C (66.30), H (9.15), N (0.87); GPC (DMF, PS-standard): M_n = 27,000 g/mol; M_w = 43,100 g/mol; $Đ$ = 1.60.

Procedure for preparation of polymer **P5**:

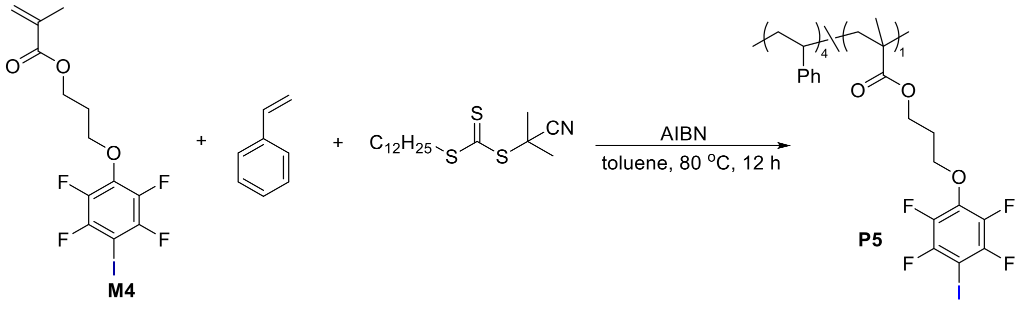

To a 50 mL Schlenk tube containing a reaction mixture of **M4** (7.0 mmol), styrene (27.8 mmol), AIBN, and 2-cyano-2-propyl dodecyl trithiocarbonate (CTA) was added anhydrous toluene (35.0 mL). The ratio of [CTA] to [AIBN] was always 5/1 and the ratio of [**M4** + styrene] to [CTA] was 500/1. Above mixture was deoxygenated by nitrogen purging for 15 min and stirred at 80 °C for 24 h. The solvent was removed using rotatory evaporation. The residue was washed with cold *n*-pentane and methanol.
P5: ^{1}H NMR (400 MHz, CD_2Cl_2): δ 7.12–6.67 (m, 20H), 4.09–3.82 (m, 4H), 1.66–1.15 (m, 19H); ^{19}F NMR (376 MHz, $CDCl_3$): δ -121.01, -154.29. Anal. (found): C (64.42), H (5.35); GPC (DMF, PS-standard): M_n = 17,100 g/mol; M_w = 25,400 g/mol; $Đ$ = 1.49.

Procedure for preparation of polymer **P6**:

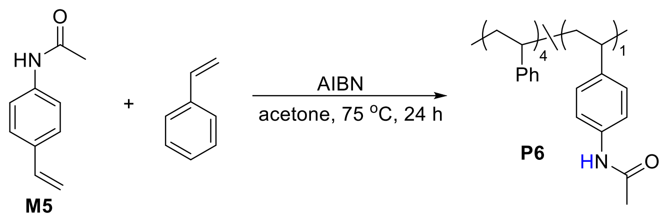

P6 was prepared according to the literature procedures [5]. ^{1}H NMR (400 MHz, $CDCl_3$): δ 7.07–6.58 (m, 24H), 2.17 (s, 3H), 1.86–1.68 (m, 5H), 1.55–1.26 (m, 10H); Anal. (found): C (87.23), H (7.76), N (2.40); GPC (DMF, PS-standard): M_n = 11,900 g/mol; M_w = 28,300 g/mol; $Đ$ = 2.38.

Procedure for preparation of **P^{III}-PNs**, **Sb^{III}-PNs**, **Sb^{III}-PNs', Sb^{V}-PNs**, **XB-PNs** and **HB-PNs**:

The desired amounts of the two polymers (**educt 1** and **educt 2**) were dissolved in 30.0 mL chloroform. Therefore, the amount of functional groups per copolymer chain was utilized for the calculation of the stoichiometric ratio. Subsequently, the two solutions were mixed and stirred at room temperature for 5 min. The solvent was evaporated slowly and the final product was dried under reduced pressure. The exact amounts of all reagents are summarized in **Table S1**.

Table S2. Overview of the used amounts for the crosslinking of the polymers.

polymer networks	**educt 1**	m (**educt 1**) [mg]	**educt 2**	m (**educt 2**) [mg]
**P^{III}-PNs**	**P1**	500.00 mg	**P4**	755.63 mg
**Sb^{III}-PNs**	**P2**	500.00 mg	**P4**	670.10 mg
**Sb^{III}-PNs'**	**P2**	500.00 mg	**P4'**	670.10 mg
**Sb^{V}-PNs**	**P3**	500.00 mg	**P4**	545.66 mg
**XB-PNs**	**P5**	500.00 mg	**P4**	865.50 mg
**HB-PNs**	**P6**	500.00 mg	**P4**	1252.35 mg

P^{III}-PNs: ^{1}H NMR (400 MHz, $CDCl_3$): δ 8.60 (s, 2H), 8.11 (s, 6H), 7.00–6.53 (m, 26H), 5.09 (s, 2H), 4.03 (s, 20H), 2.29–0.83 (m, 158H); ^{19}F NMR (376 MHz, $CDCl_3$): δ -62.97.

Sb^{III}-PNs: ^{1}H NMR (400 MHz, $CDCl_3$): δ 8.61–8.59 (m, 2H), 7.88–7.80 (m, 6H), 7.00–6.54 (m, 26H), 5.09 (s, 2H), 4.03 (s, 20H), 2.29–0.88 (m, 149H); ^{19}F NMR (376 MHz,

$CDCl_3$): δ -62.94.

Sb^{III}-PNs': ^{1}H NMR (400 MHz, $CDCl_3$): δ 8.61–8.59 (m, 2H), 7.88–7.80 (m, 6H), 7.02–6.55 (m, 26H), 5.10–5.06 (m, 2H), 4.05–4.00 (m, 20H), 2.28–0.91 (m, 131H); ^{19}F NMR (376 MHz, $CDCl_3$): δ -62.91.

Sb^{V}-PNs: ^{1}H NMR (400 MHz, CD_2Cl_2): δ 8.20–8.02 (m, 8H), 7.32–6.57 (m, 26H), 5.10 (s, 2H), 4.03 (s, 20H), 2.27–0.84 (m, 163H); ^{19}F NMR (376 MHz, $CDCl_3$): δ -62.92.

XB-PNs: ^{1}H NMR (400 MHz, $CDCl_3$): δ 8.60 (d, *J* = 4.9 Hz, 2H), 7.09–6.66(m, 22H), 5.10–5.02(m, 2H), 4.05–3.99 (m, 24H), 2.29–0.86 (m, 143H); ^{19}F NMR (376 MHz, $CDCl_3$): δ -121.09, -154.27.

HB-PNs: ^{1}H NMR (400 MHz, $CDCl_3$): δ 8.60 (d, *J* = 5.0 Hz, 2H), 7.08–6.55 (m, 26H), 5.10–5.06 (m, 2H), 4.09–4.01 (m, 20H), 2.28–0.85 (m, 150H).

Procedure for preparation of polymer **Sb$^{V/III}$-PNs**:

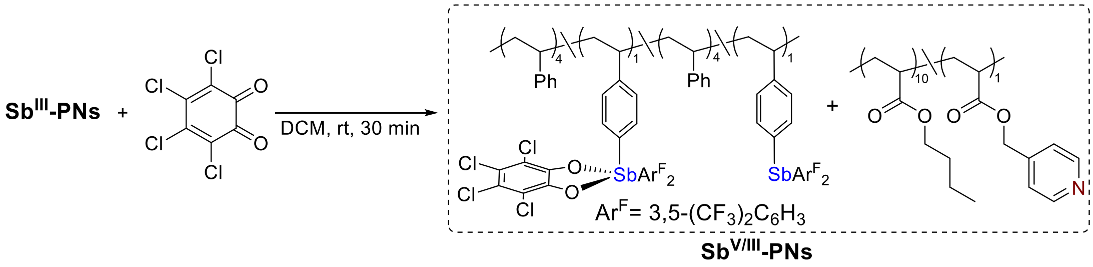

Reactants **Sb^{III}-PNs** (1.1 g) and 3,4,5,6-tetrachlorocyclohexa-3,5-diene-1,2-dione (49.2 mg) were added to anhydrous DCM (30.0 mL) under nitrogen atmosphere. The above reaction mixture was stirred at room temperature for 30 min and concentrated under reduced pressure. The solid was dried in a vacuum oven at 50 °C to afford the target polymer.

Sb$^{V/III}$-PNs: ^{1}H NMR (400 MHz, $CDCl_3$): δ 8.58 (s, 2H), 8.07–7.98 (m, 3H), 7.88–7.80 (m, 3H), 7.00–6.56 (m, 26H), 5.09 (s, 2H), 4.03 (s, 20H), 2.29–0.86 (m, 170H); ^{19}F NMR (376 MHz, $CDCl_3$): δ -62.94.

3. The interaction between donor polymers and acceptor polymers

The interaction between **P1** and **P4**:

At ambient temperature (298.5 K), **P1** (0.005 mmol) and **P4** were dissolved in 0.5 mL CD_2Cl_2. Then the mixtures were analyzed by ^{1}H NMR experiments.

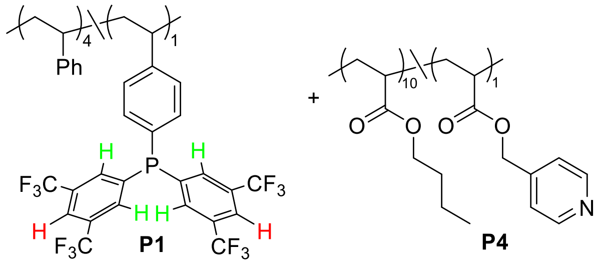

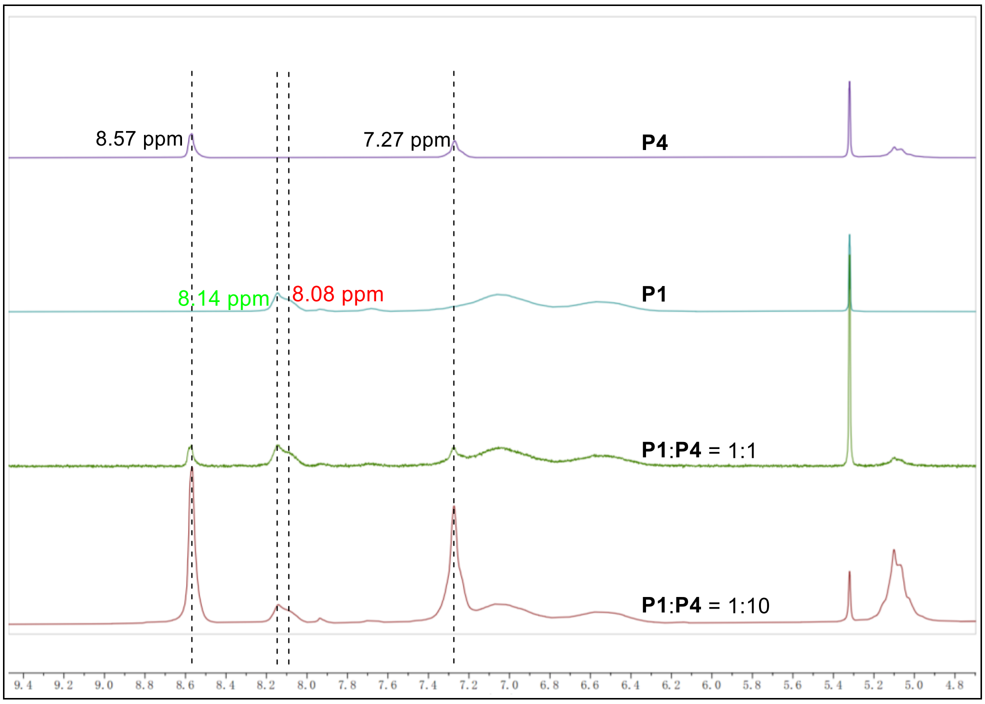

Figure S1. ^{1}H NMR spectroscopy for investigating the interaction between **P1** and **P4**.

At ambient temperature (298.5 K), **P1** (0.005 mmol) and **P4** (1.0 equiv, 0.05 mmol) were dissolved in 0.5 mL $CDCl_3$. Then the mixtures were analyzed by ^{13}C NMR experiments.

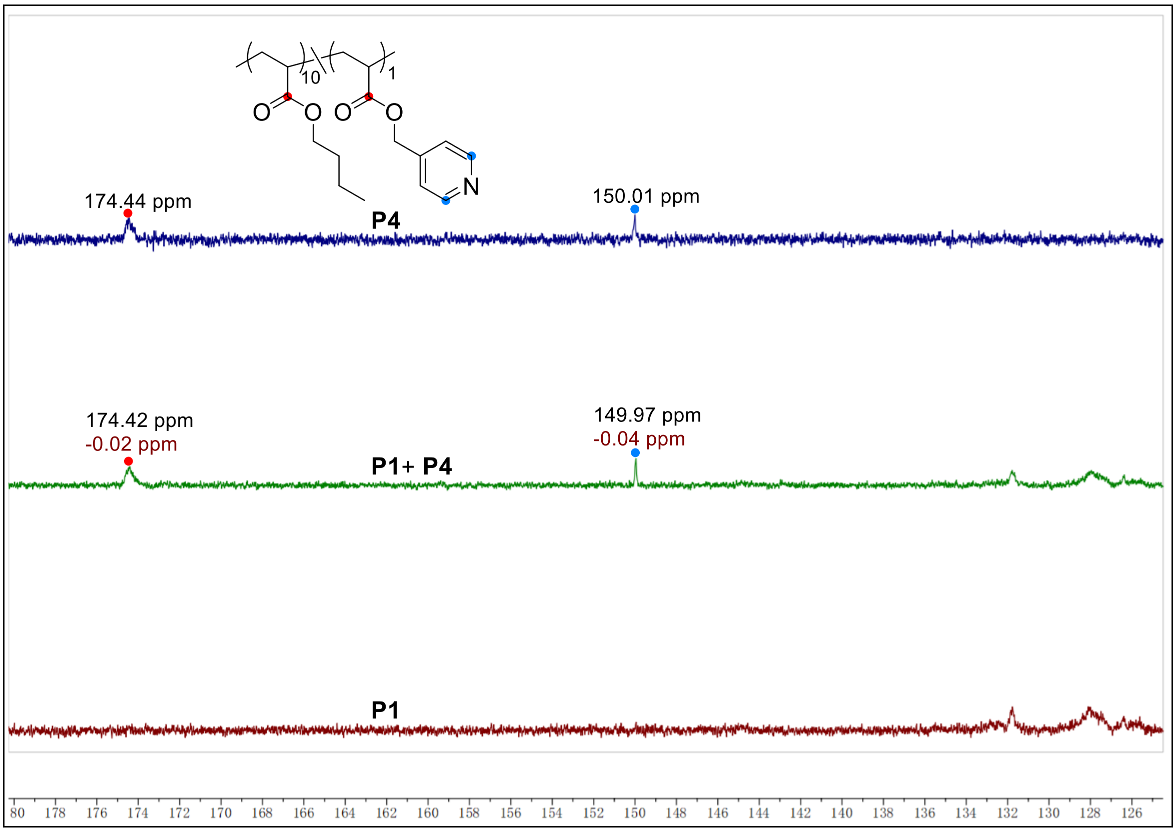

Figure S2. ^{13}C NMR spectroscopy for investigating the interaction between **P1** and **P4**.

The interaction between **P2** and **P4**:

At ambient temperature (298.5 K), **P2** (0.005 mmol) and **P4** were dissolved in 0.5 mL CD_2Cl_2. Then the mixtures were analyzed by ^{1}H NMR experiments.

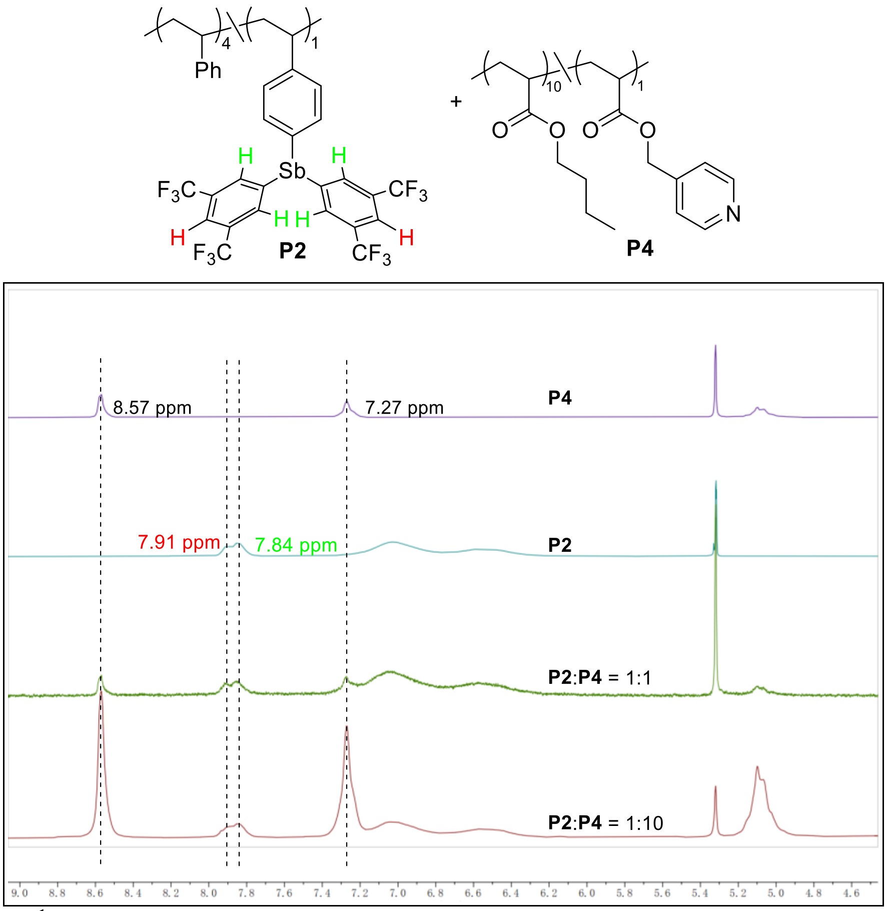

Figure S3. ^{1}H NMR spectroscopy for investigating the interaction between **P2** and **P4**.

At ambient temperature (298.5 K), **P2** (0.005 mmol) and **P4** (1.0 equiv, 0.05 mmol) were dissolved in 0.5 mL $CDCl_3$. Then the mixtures were analyzed by ^{13}C NMR experiments.

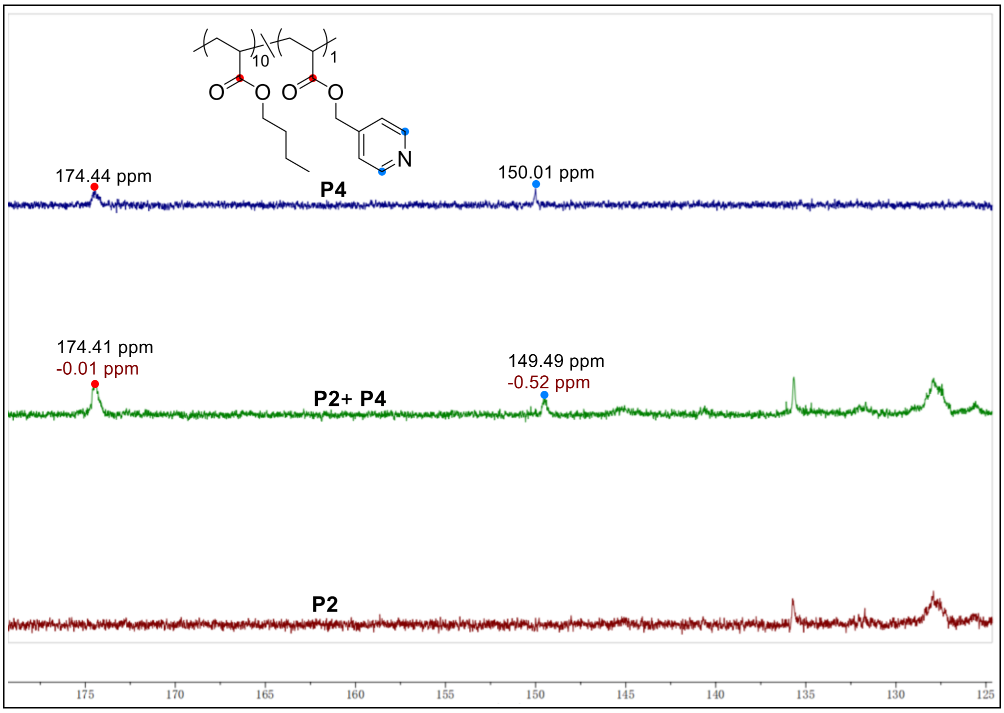

Figure S4. ^{13}C NMR spectroscopy for investigating the interaction between **P2** and **P4**.

^{1}H NMR titration experiments between **P3** and **P4**:

All experiments were conducted by mixing different ratios of the **P3** and **P4** at ambient temperature (298.5 K) in NMR tubes. **P3** (0.005 mmol) and the different equivalent of **P4** (0 equiv, 0.3 equiv, 0.6 equiv, 0.9 equiv, 1.5 equiv, 2 equiv) were dissolved in 0.5 mL $CDCl_3$. Then the mixtures were analyzed by ^{1}H NMR experiments.

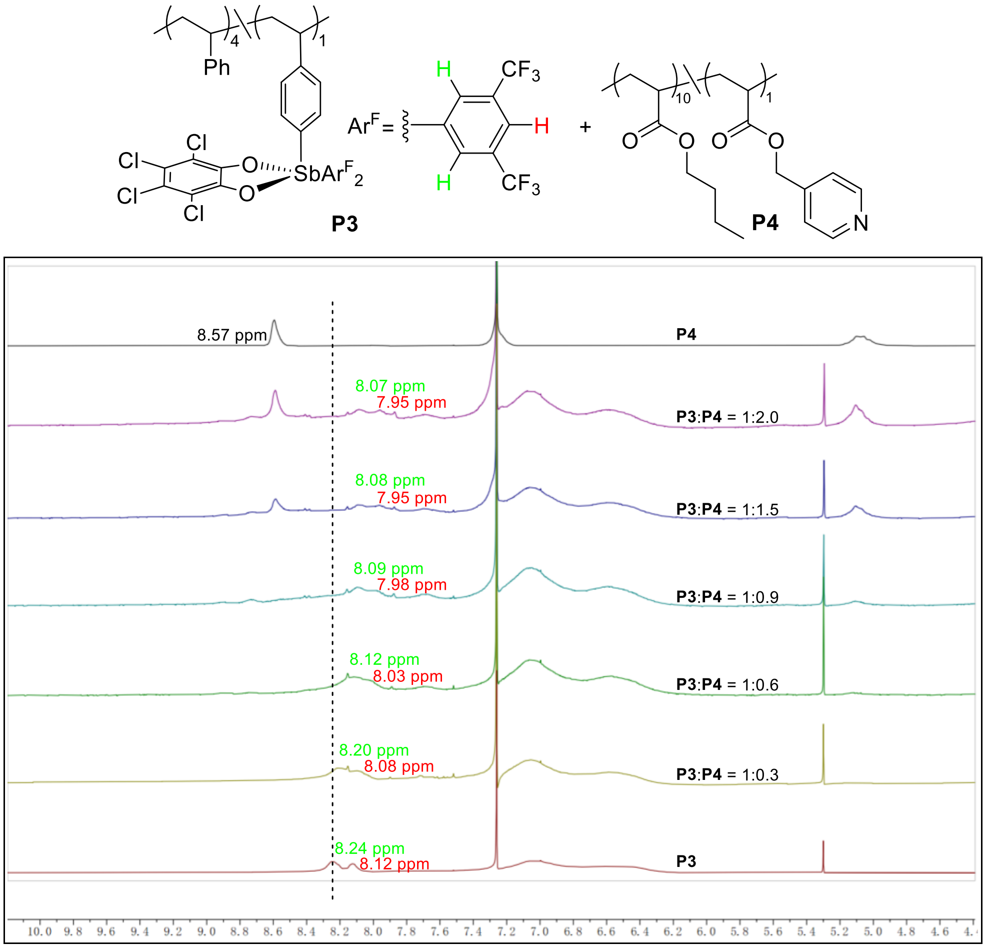

Figure S5. ^{1}H NMR titration experiments between **P3** with **P4**. (The weak resonance signals observed at 8.15 ppm and 7.87 ppm are likely attributable to aggregation of **P3** and **P4**.)

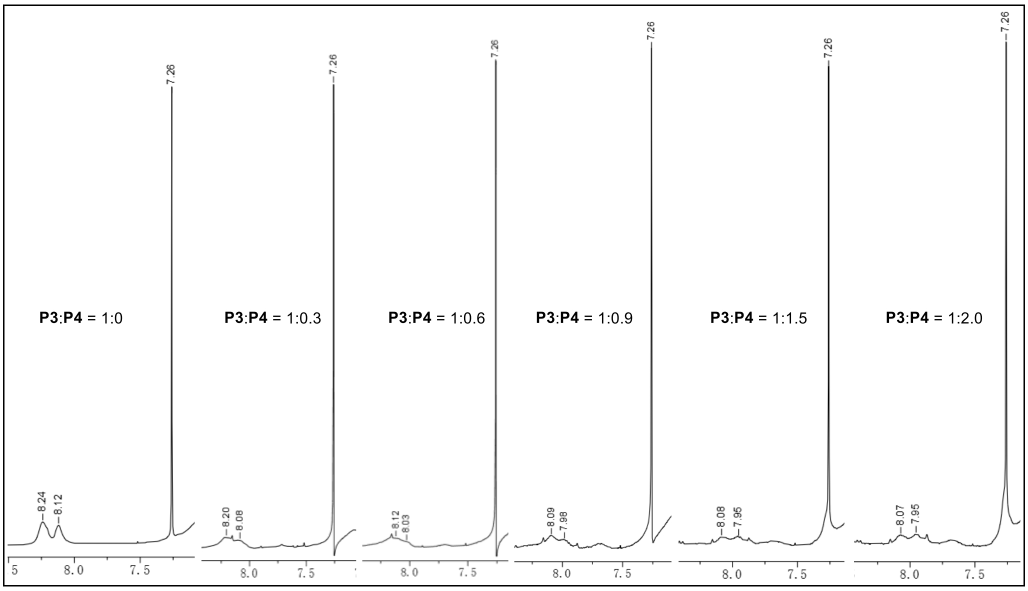

Figure S6. ^{1}H NMR titration experiments between **P4** with **P3**.

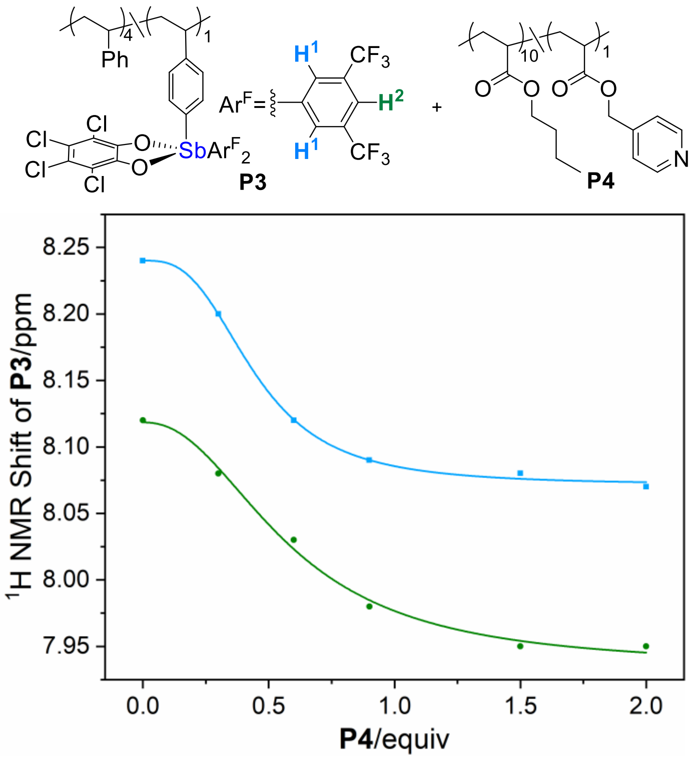

Figure S7. ^{1}H NMR titration curves of **P4** and **P3**.

At ambient temperature (298.5 K), **P3** (0.005 mmol) and **P4** (1.0 equiv, 0.05 mmol) were dissolved in 0.5 mL $CDCl_3$. Then the mixtures were analyzed by ^{13}C NMR experiments.

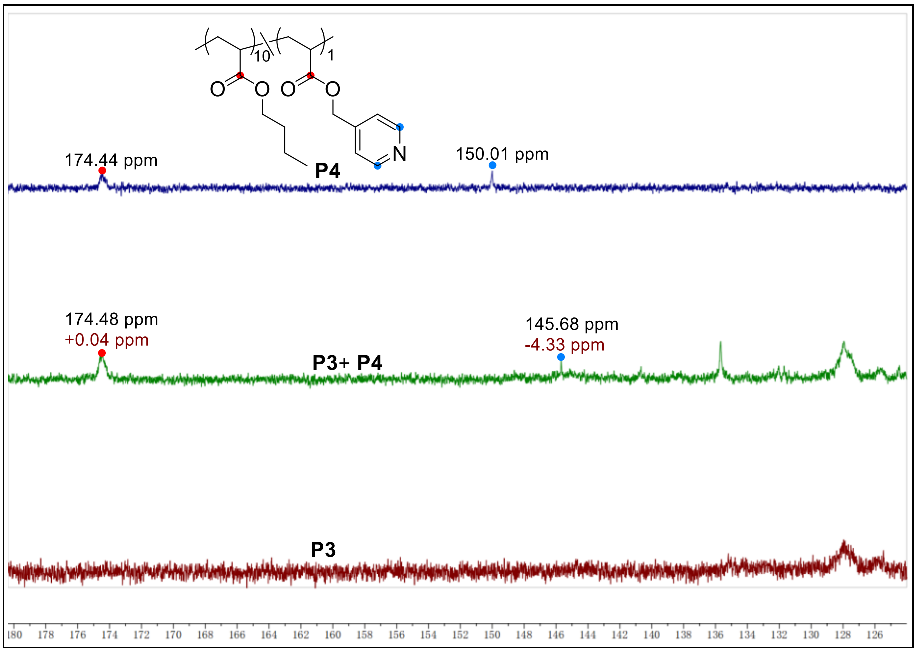

Figure S8. ^{13}C NMR spectroscopy for investigating the interaction between **P3** and **P4**.

^{1}H NMR titration experiments between monomer **3** and **M2**:

All experiments were conducted by mixing different ratios of the **3** and **M2** at ambient temperature (298.5 K) in NMR tubes. **3** (0.005 mmol) and the different equivalent of **M2** (0 equiv, 0.3 equiv, 0.6 equiv, 0.9 equiv, 1.5 equiv, 2 equiv) were dissolved in 0.5 mL $CDCl_3$. Then the mixtures were analyzed by ^{1}H NMR experiments.

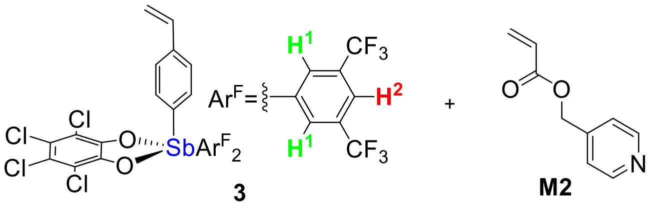

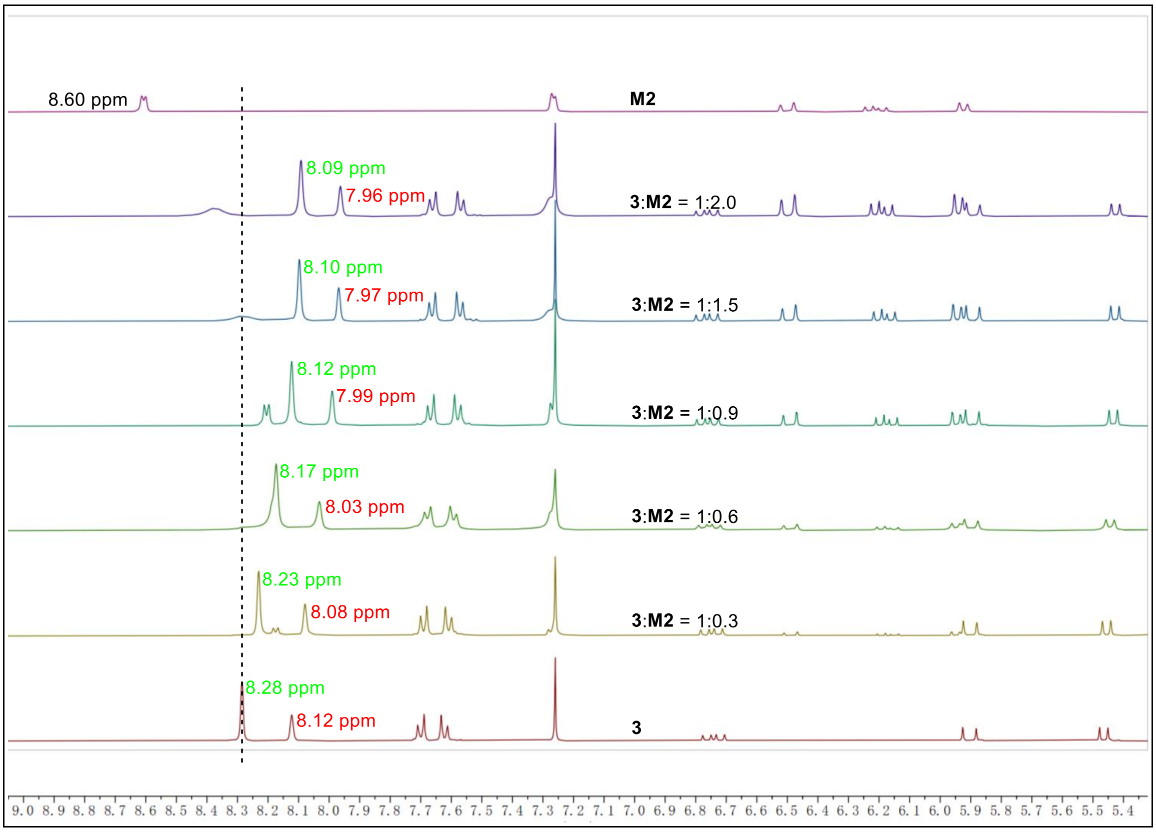

Figure S9. ^{1}H NMR titration experiments between **M2** with **3**.

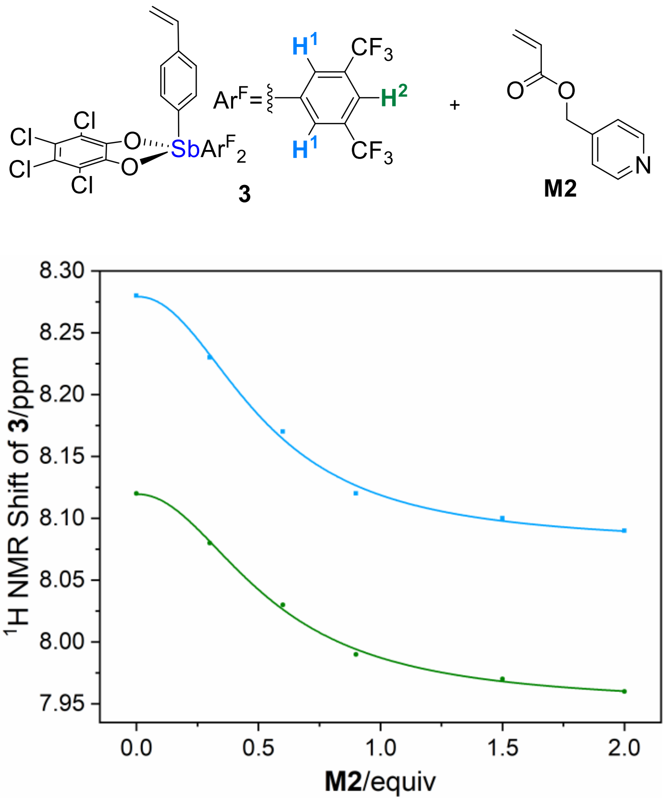

Figure S10. ^{1}H NMR titration curves of **M2** and **3**.

For the determination of the binding constants (K) of donor and acceptor complexes, the shift of the H^1 and H^2 of **P3** (or **3**) were observed relative to the signal of the solvent. The measured shifts were plotted against the guest equivalents and the resulting curve was fitted via http://supramolecular.org[4,5]. For the calculations of the binding constants (K), a 1:1 binding was assumed.

Table S3. The binding constants (K) of donor and acceptor.

Donor	Acceptor	Binding constants (K)
**P3**	**P4**	1.57×10^4 M^{-1}
**3**	**M2**	0.69×10^4 M^{-1}

4. Self assembly studies

In a representative experiment, a solution of pnictogen-bonding acceptor polymer (0.0048 mmol in 2.0 mL THF) was introduced via syringe pump to a solution of pnictogen-bonding donor polymer (0.0048 mmol in 2.0 mL THF) under continuous stirring (60 rpm). Simultaneously, deionized water (16.0 mL) was delivered through a

separate syringe pump channel at a constant infusion rate (1.07 mL/h) over 15 h. The mixture progressively developed a translucent colloidal dispersion exhibiting characteristic opalescence, serving as a visual indicator of successful polymer complexation. The resulting dispersion was aged at room temperature for 10 h prior to characterization by transmission electron microscopy (TEM) and dynamic light scattering (DLS).

5. Thermogravimetric analysis (TGA)

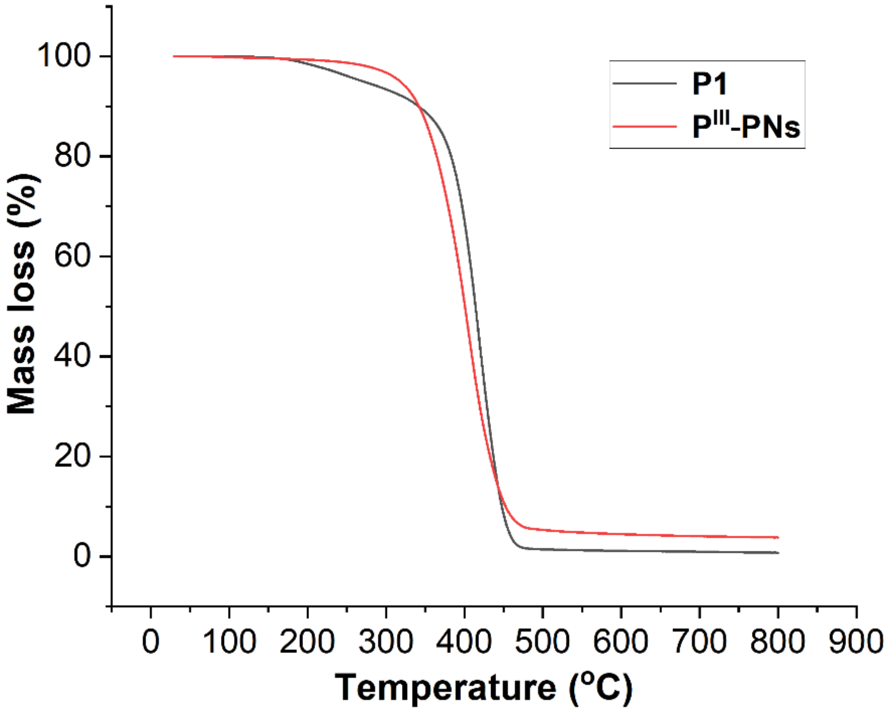

Figure S11. TGA curves of the polymer **P1** and **P^{III}-PNs**.

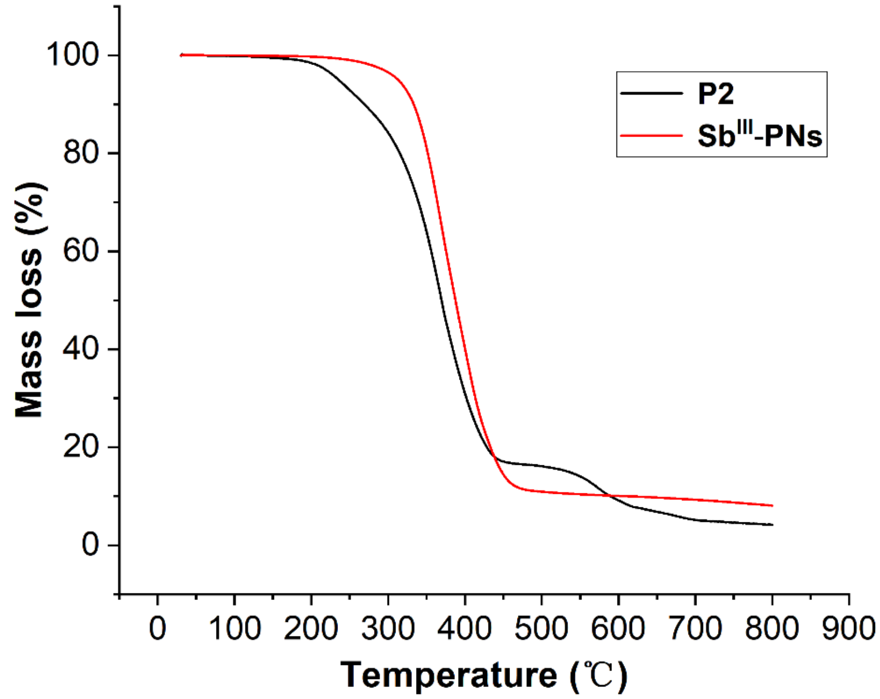

Figure S12. TGA curves of the polymer **P2** and **Sb^{III}-PNs**.

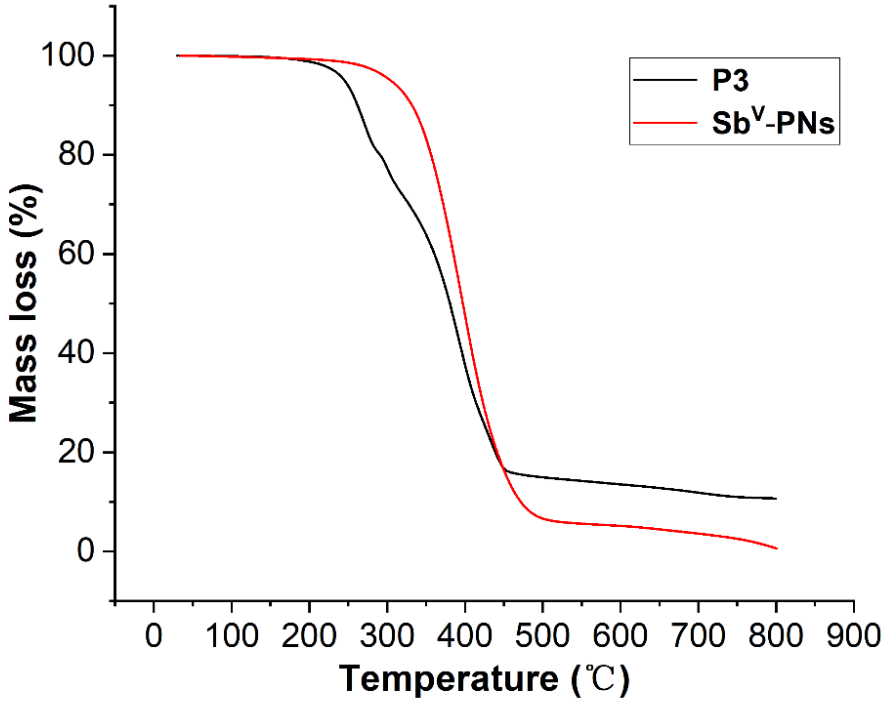

Figure S13. TGA curves of the polymer **P3** and **Sb^{V}-PNs**.

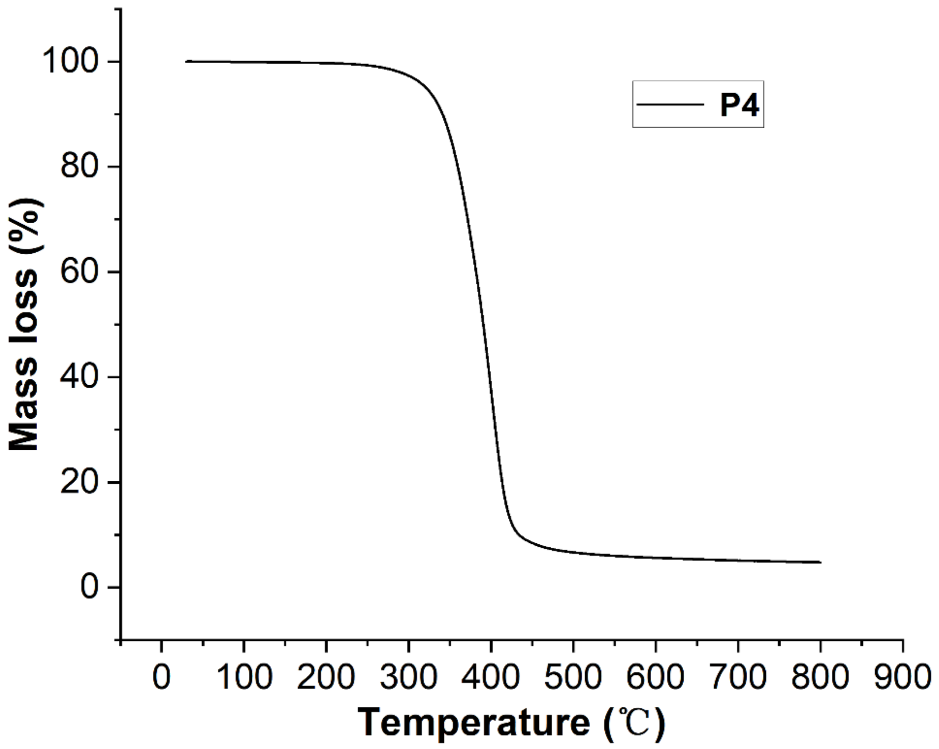

Figure S14. TGA curve of the polymer **P4**.

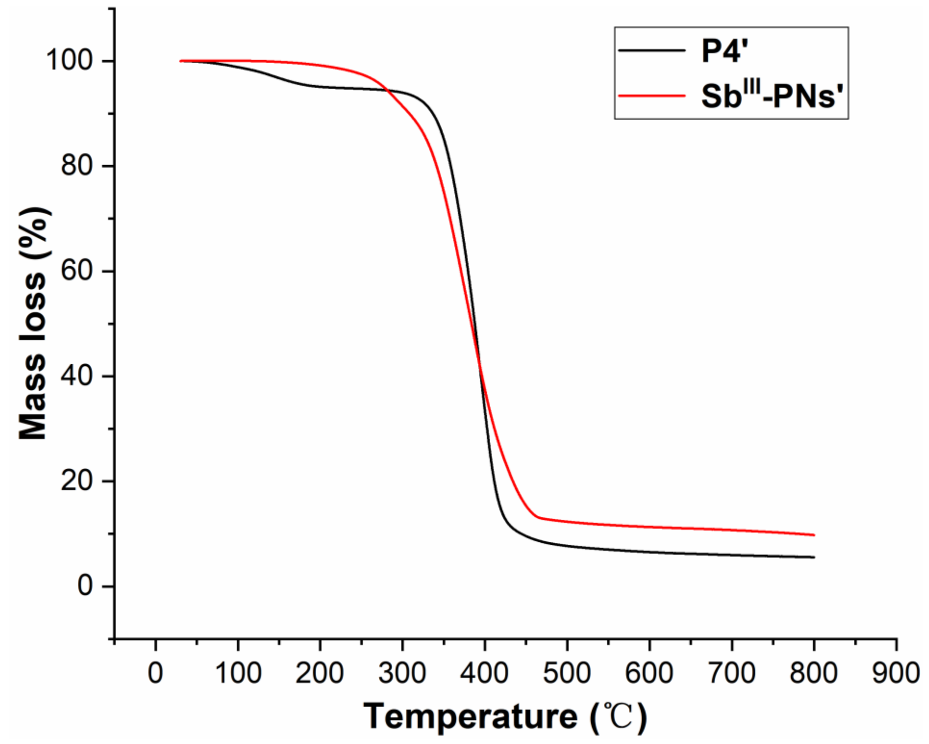

Figure S15. TGA curve of the polymer **P4'** and **Sb^{III}-PNs'**.

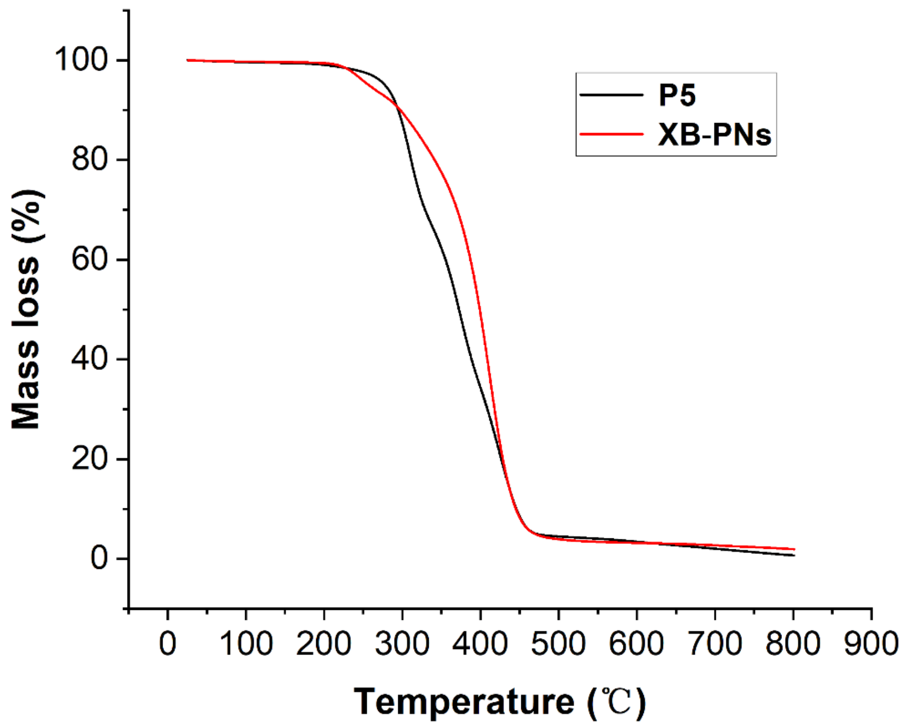

Figure S16. TGA curves of the polymer **P5** and **XB-PNs**.

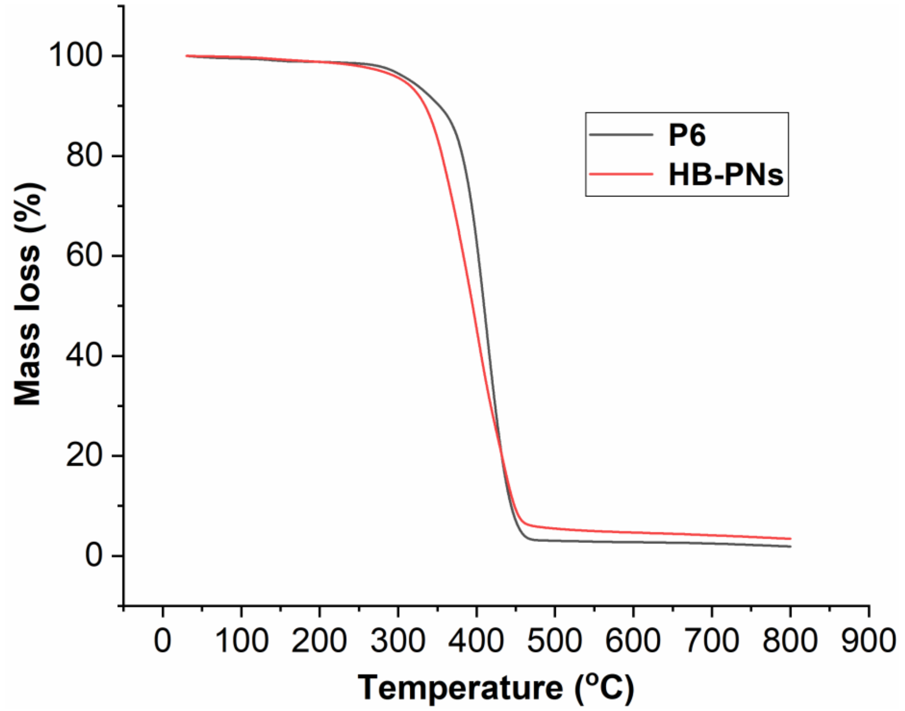

Figure S17. TGA curves of the polymer **P6** and **HB-PNs**.

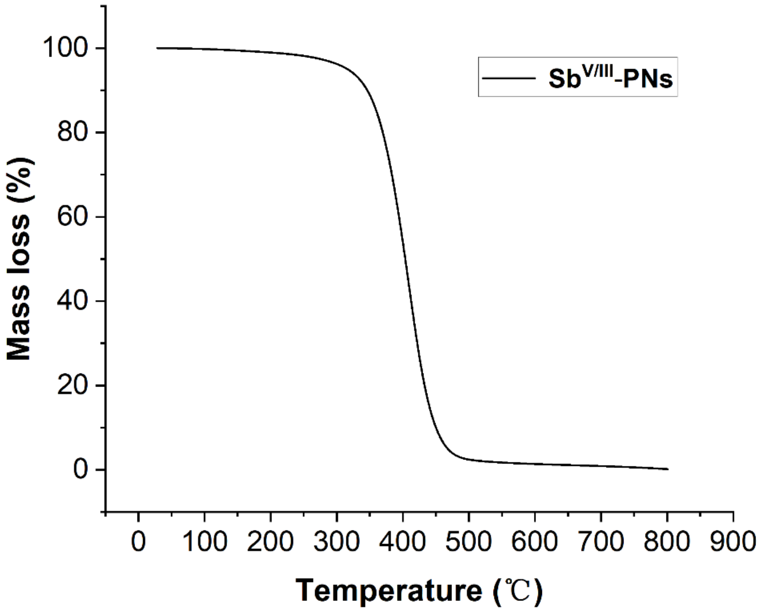

Figure S18. TGA curve of the polymer **$Sb^{V/III}$-PNs**.

6. Differential scanning calorimetry (DSC)

For DSC measurements, a small amount of sample (5.0 mg) was placed in a non-hermitic crimp pan and sealed. The sample underwent two consecutive heating/cooling cycles between -80 °C and 160 °C at a scanning rate of 10 K·min^{-1}. A 2 min isothermal hold was applied at each temperature extreme. Nitrogen purge gas was supplied at a flow rate of 20 mL·min^{-1}. The glass transition temperature (T_g) was determined from the second heating curve.

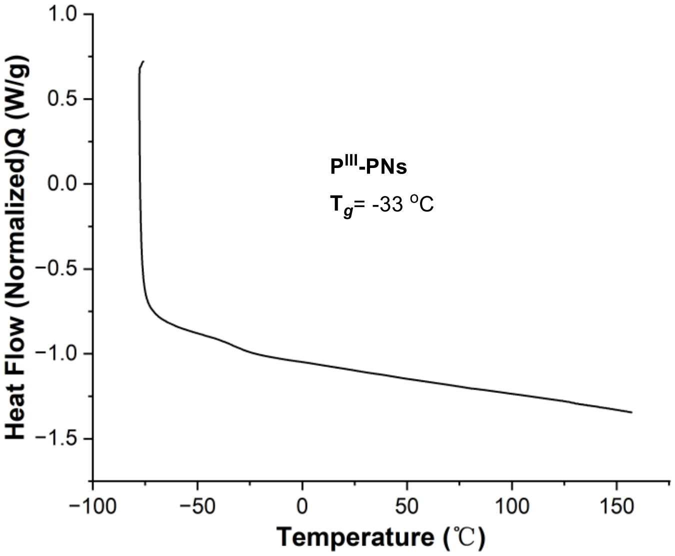

Figure S19. DSC curve of the polymer **P^{III}-PNs**.

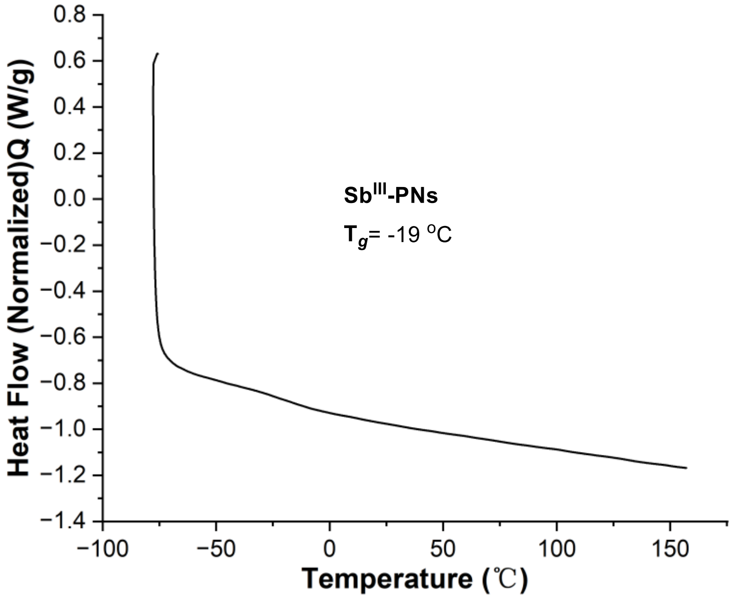

Figure S20. DSC curve of the polymer **Sb^{III}-PNs**.

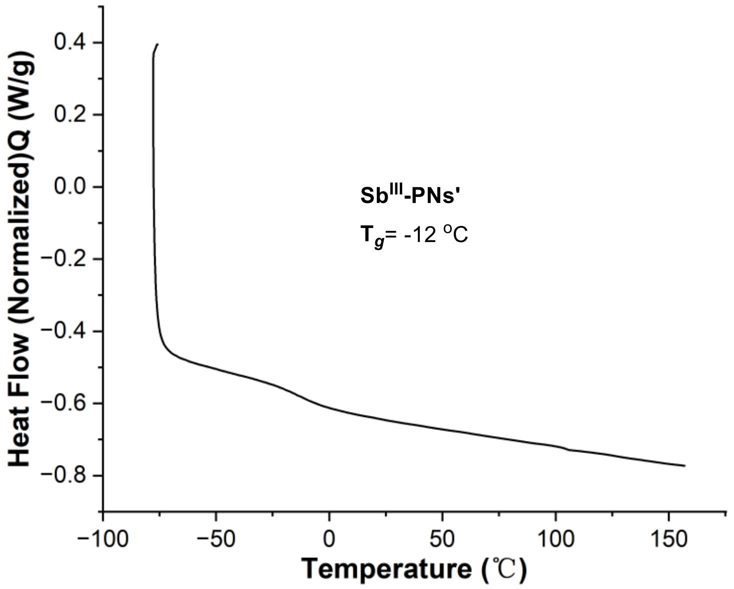

Figure S21. DSC curve of the polymer **Sb^{III}-PNs'**.

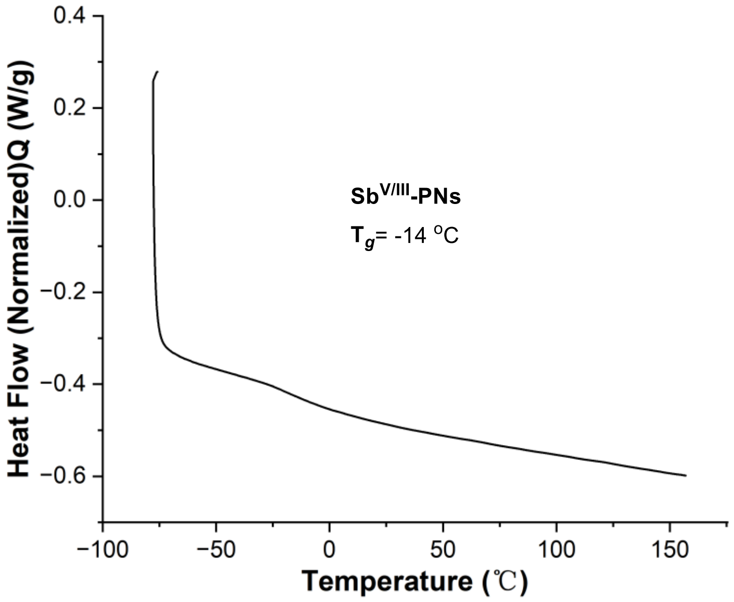

Figure S22. DSC curve of the polymer **Sb$^{V/III}$-PNs**.

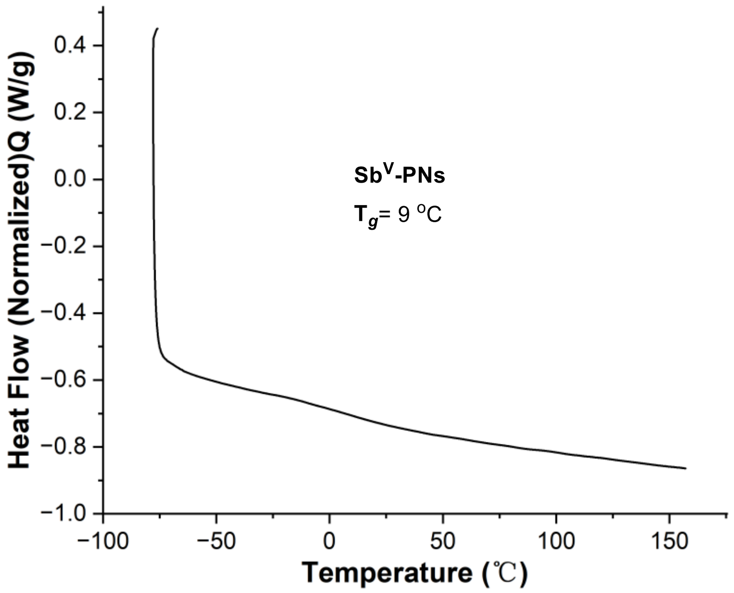

Figure S23. DSC curve of the polymer **Sb^{V}-PNs**.

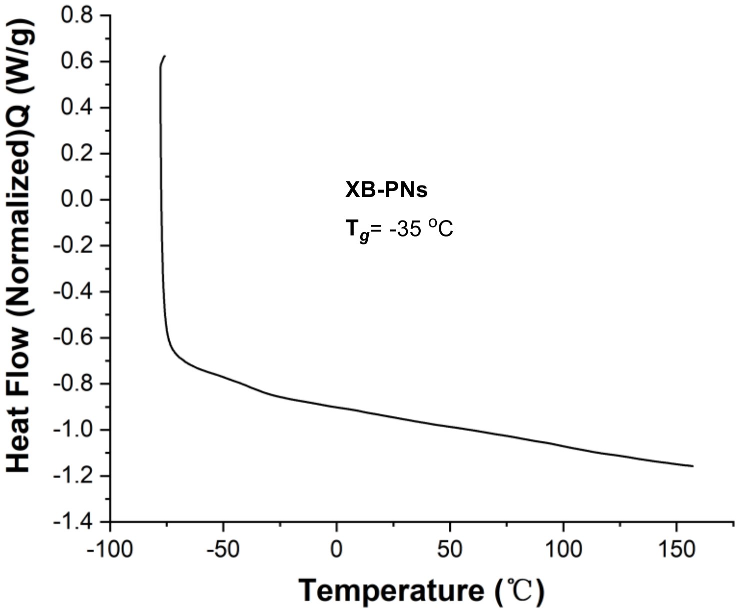

Figure S24. DSC curve of the polymer **XB-PNs**.

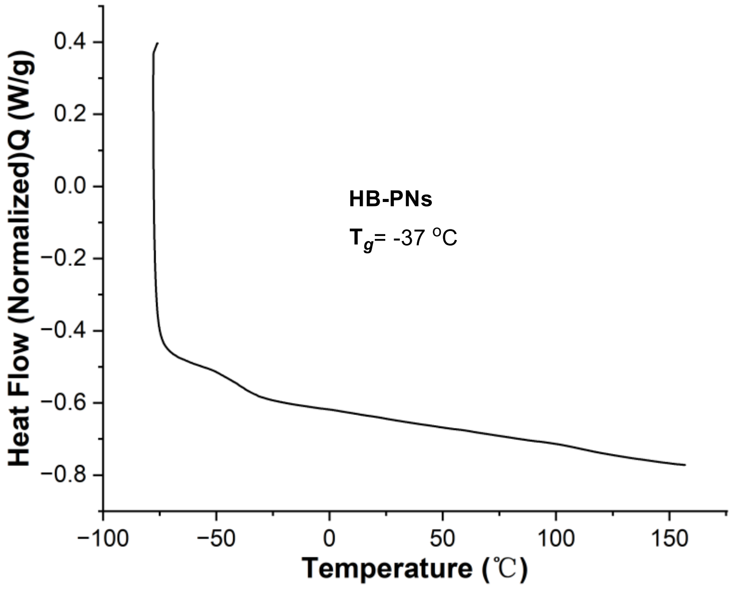

Figure S25. DSC curve of the polymer **HB-PNs**.

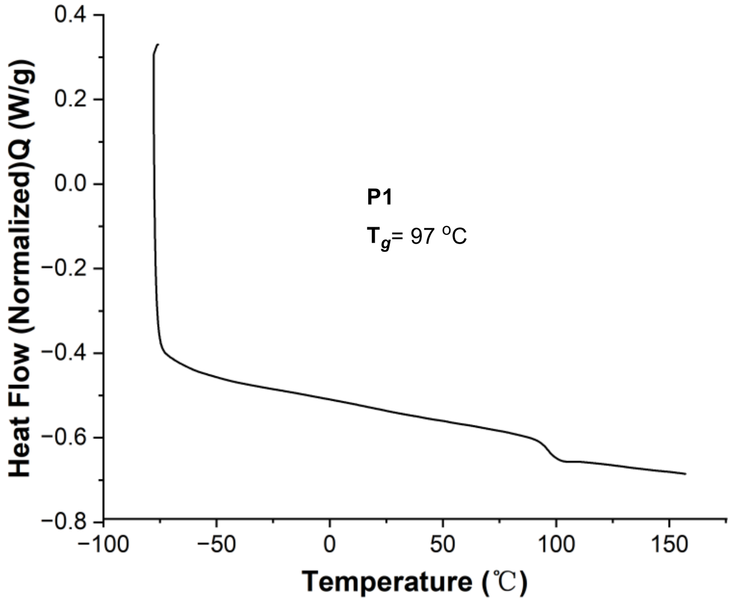

Figure S26. DSC curve of the polymer **P1**.

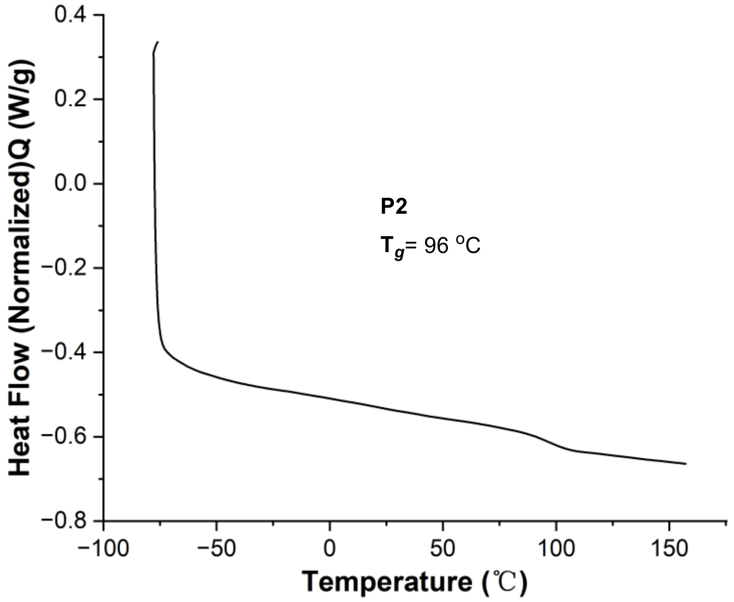

Figure S27. DSC curve of the polymer **P2**.

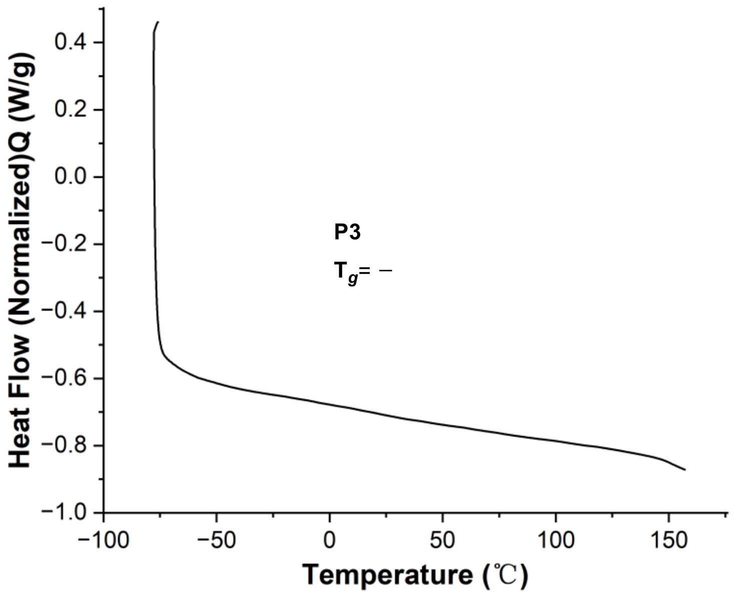

Figure S28. DSC curve of the polymer **P3**.

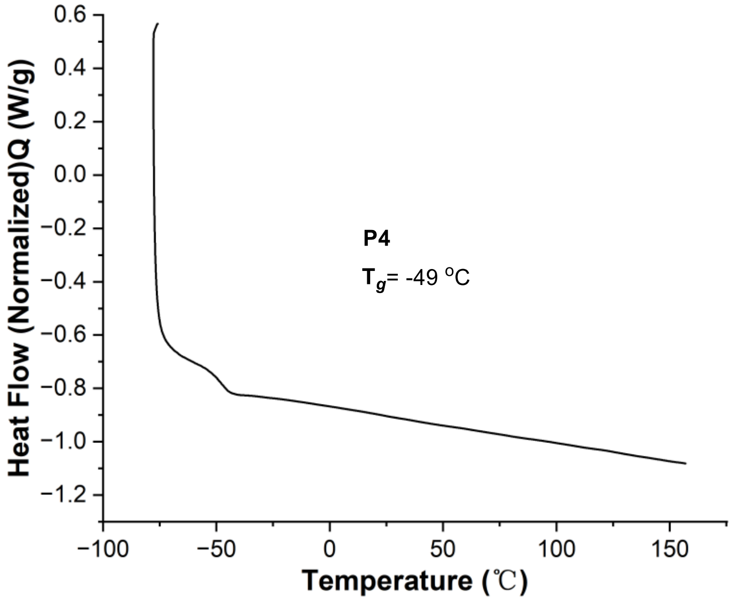

Figure S29. DSC curve of the polymer **P4**.

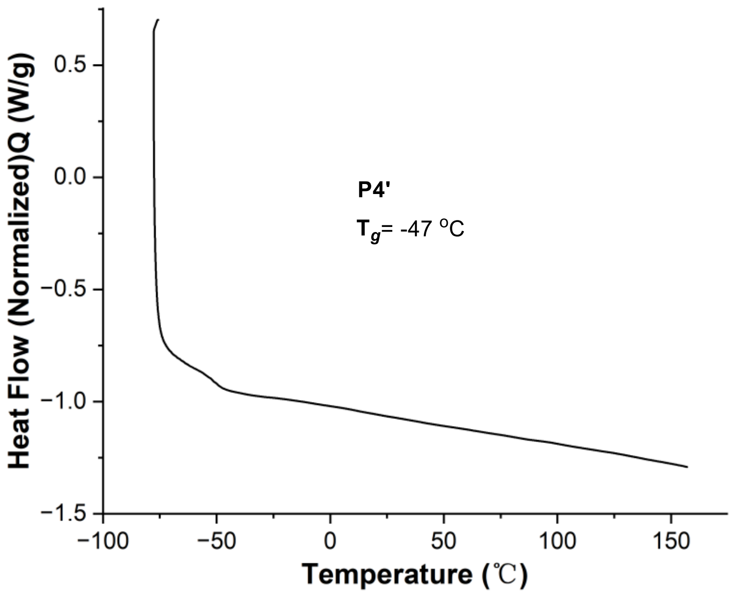

Figure S30. DSC curve of the polymer **P4'**.

7. Rheology

Viscoelastic properties of PNs were characterized by small-amplitude oscillatory shear measurements using parallel-plate geometry (20 mm diameter, 1.1 mm gap) in strain-controlled mode. Angular frequency sweeps were performed from 10^{-1} to 10^{2} rad/s.

Creep tests

Creep tests of PNs were performed at a constant load of 1000 Pa at 25 °C or 60 °C for 1000 s.

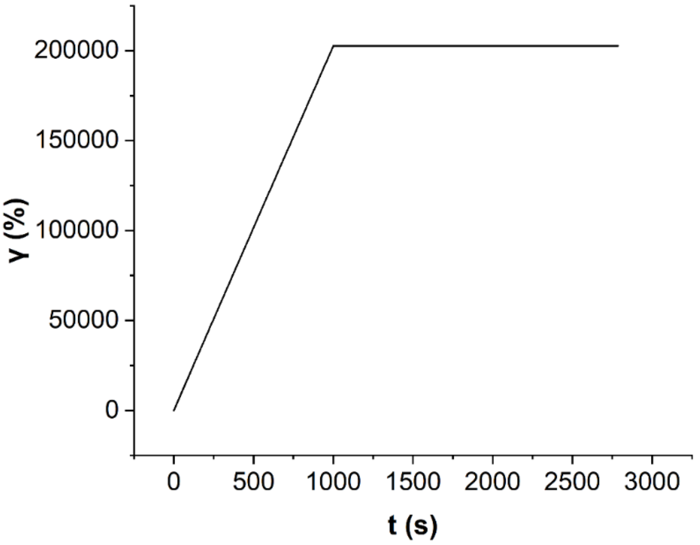

Figure S31. Creep tests curve of the polymer **P^{III}-PNs** at 25 °C.

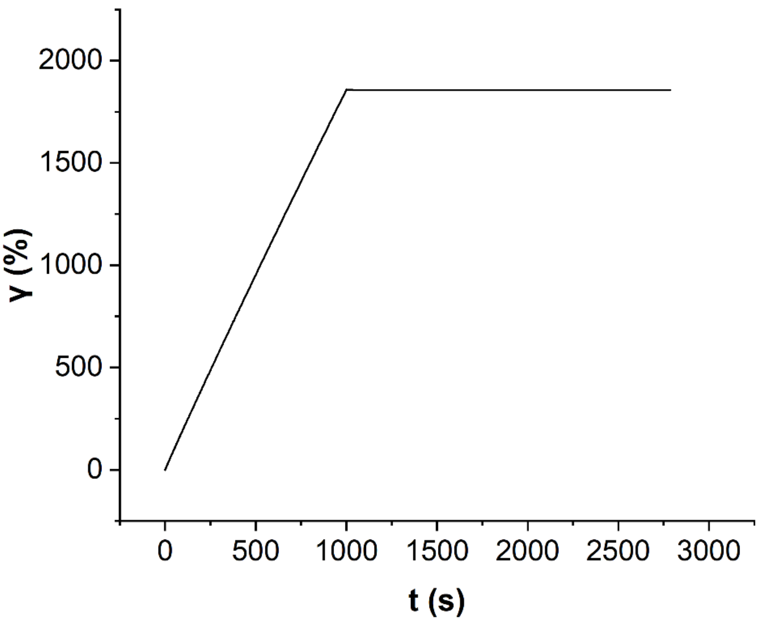

Figure S32. Creep tests curve of the polymer **Sb^{III}-PNs** at 25 °C.

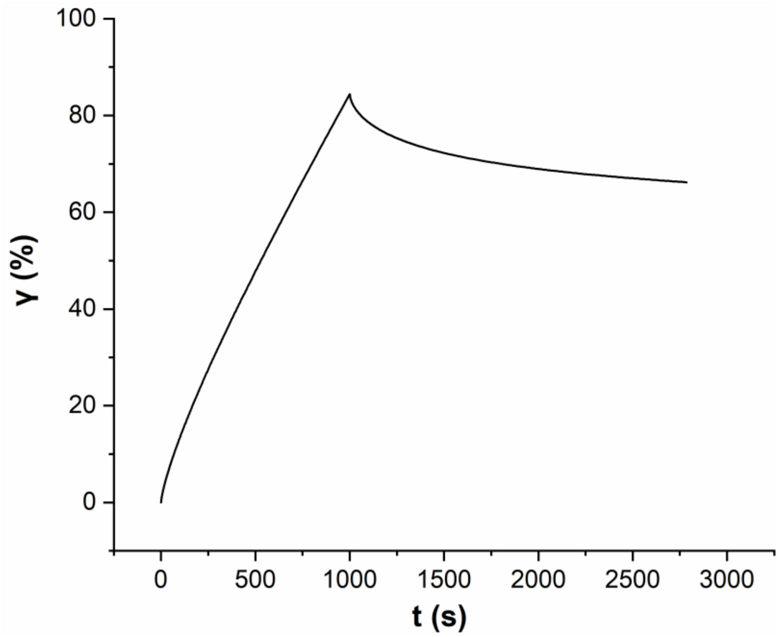

Figure S33. Creep tests curve of the polymer **Sb^{III}-PNs'** at 25 °C.

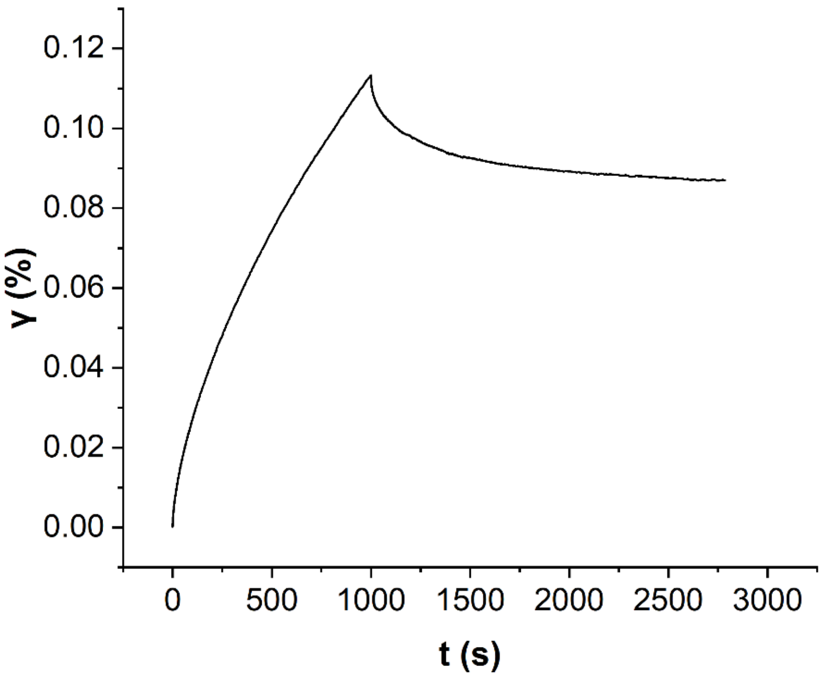

Figure S34. Creep tests curve of the polymer **Sb^{V}-PNs** at 25 °C.

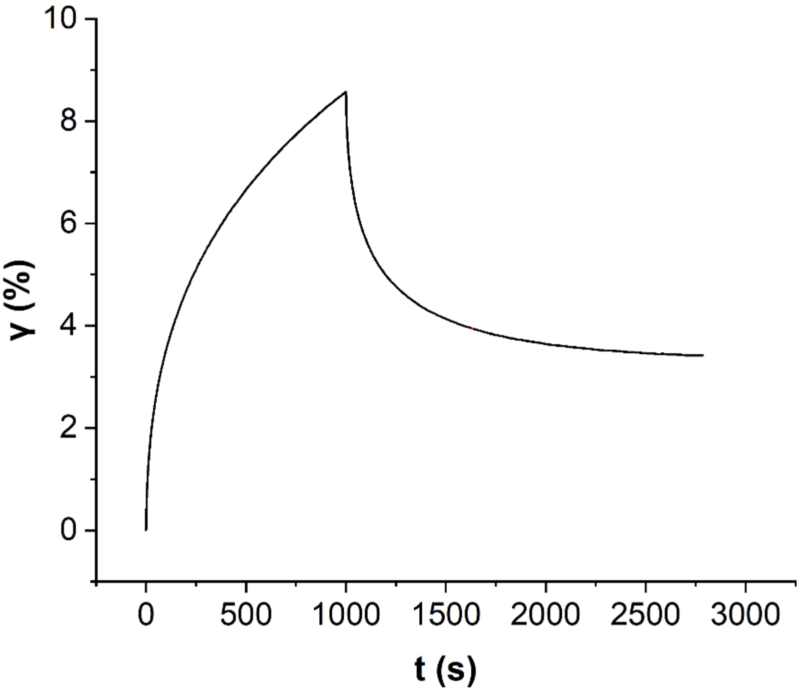

Figure S35. Creep tests curve of the polymer **Sb^{V}-PNs** at 60 °C.

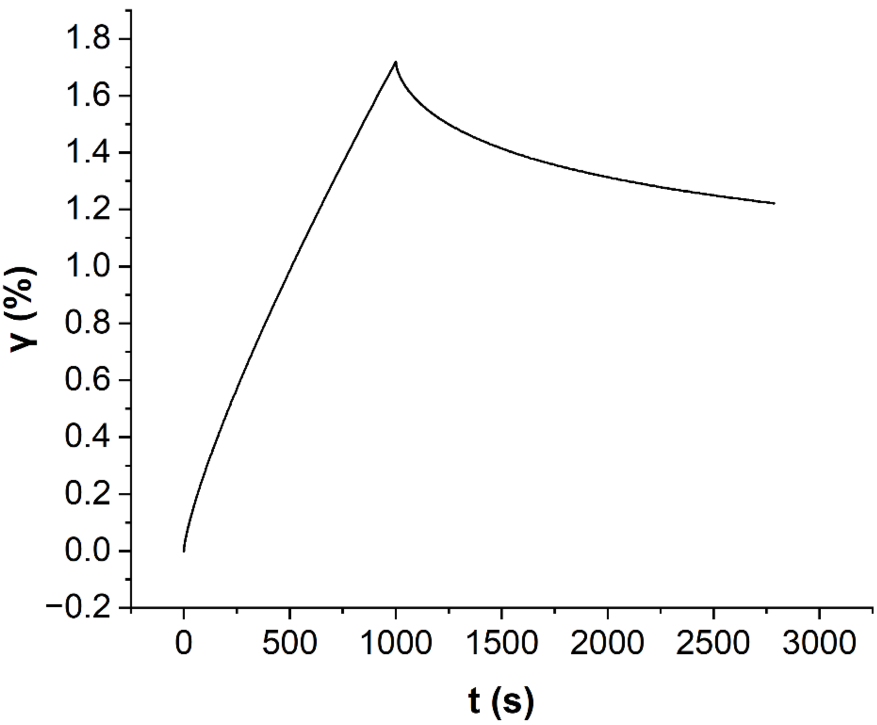

Figure S36. Creep tests curve of the polymer **$Sb^{V/III}$-PNs** at 25 °C.

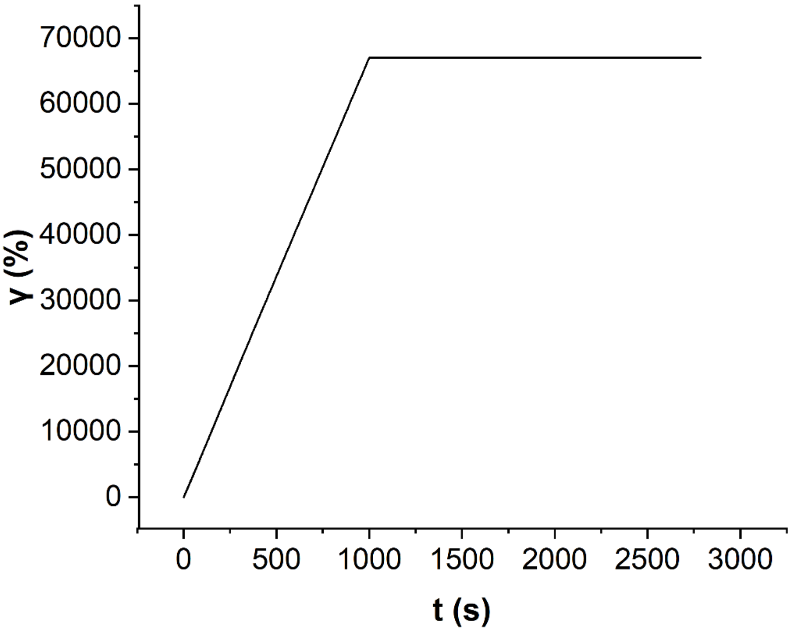

Figure S37. Creep tests curve of the polymer **XB-PNs** at 25 °C.

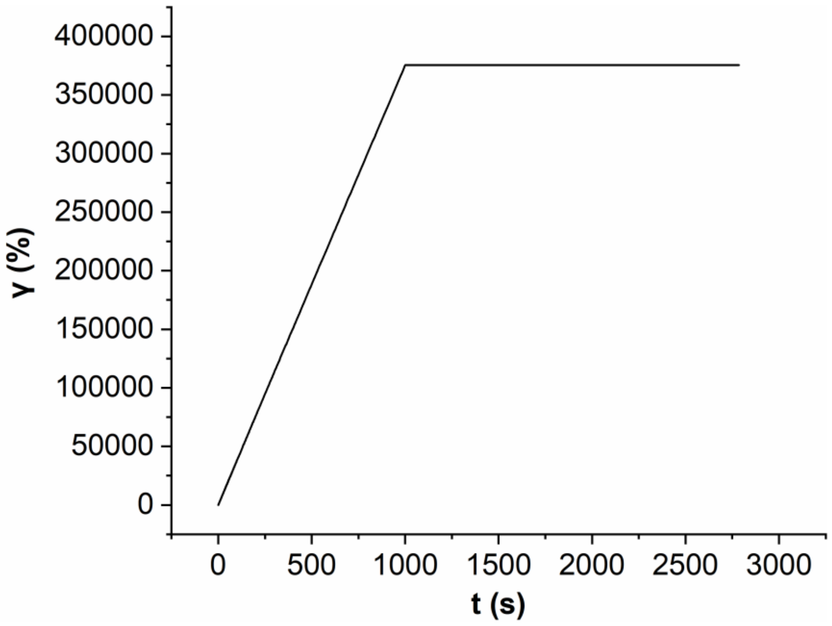

Figure S38. Creep tests curve of the polymer **HB-PNs** at 25 °C.

The **Sb^{V}-PNs** (1.0 g) and quinoline (46.8 mg, molar ratio 1:1 relative to target functional groups) were added to anhydrous DCM (30.0 mL) under nitrogen atmosphere. The above reaction mixture was stirred at room temperature for 30 min and concentrated under reduced pressure. The solid was dried in a vacuum oven at 50 °C to afford the target polymer.

Creep tests of PNs were performed at a constant load of 1000 Pa at 25 °C for 1000 s.

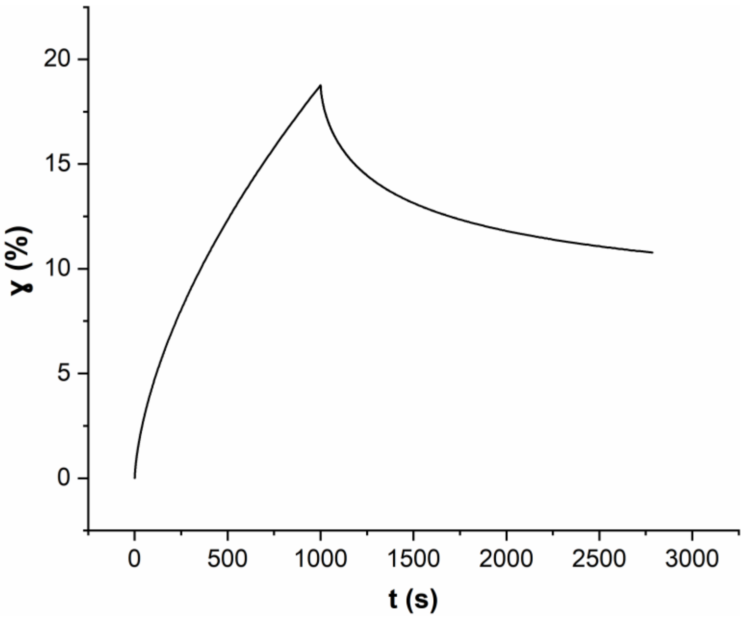

Figure S39. Creep tests curve of quinoline-containing polymer **Sb^{V}-PNs** at 25 °C.

Estimation of the relaxation activation energy (E_a)[6,7]

The shear modulus spectra were measured at four different temperatures to evaluate the relaxation activation energy (E_a). The characteristic relaxation time (τ) was determined from the crossover frequency (ω) at which the storage modulus (G') equals the loss modulus (G''), and τ was taken as $\tau = 1/\omega$.

Assuming an Arrhenius-type temperature dependence of the relaxation time, $\tau(T)$ can be expressed as:

$$\ln\tau = \ln\tau_0 + E_a/(RT)$$

where E_a is the apparent relaxation activation energy, τ_0 is the pre-exponential factor, and R is the gas constant. The Arrhenius plot of $\ln\tau$ versus $1/T$ (**Figure S42**, **S43**) exhibits a good linear relationship, confirming the validity of the Arrhenius model. From the slope of the fitted line, E_a was determined to be approximately 166 kJ mol^{-1} for **Sb^{III}-PNs** and 240 kJ mol^{-1} for **Sb^{V}-PNs**.

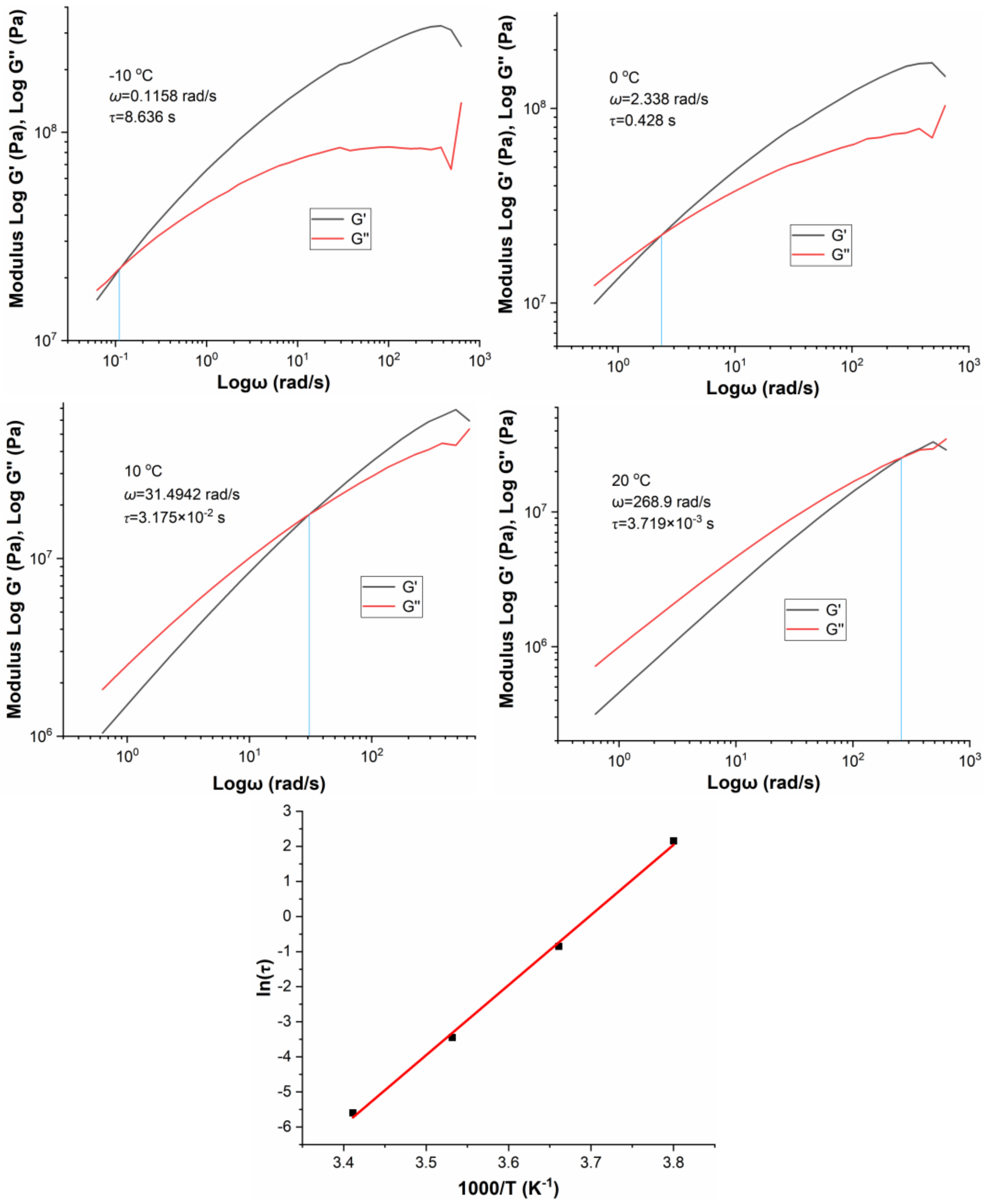

Figure S40. Temperature-dependent modulus spectra of **Sb^{III}-PNs** and Arrhenius plot (ln(τ) *vs.* 1000/T).

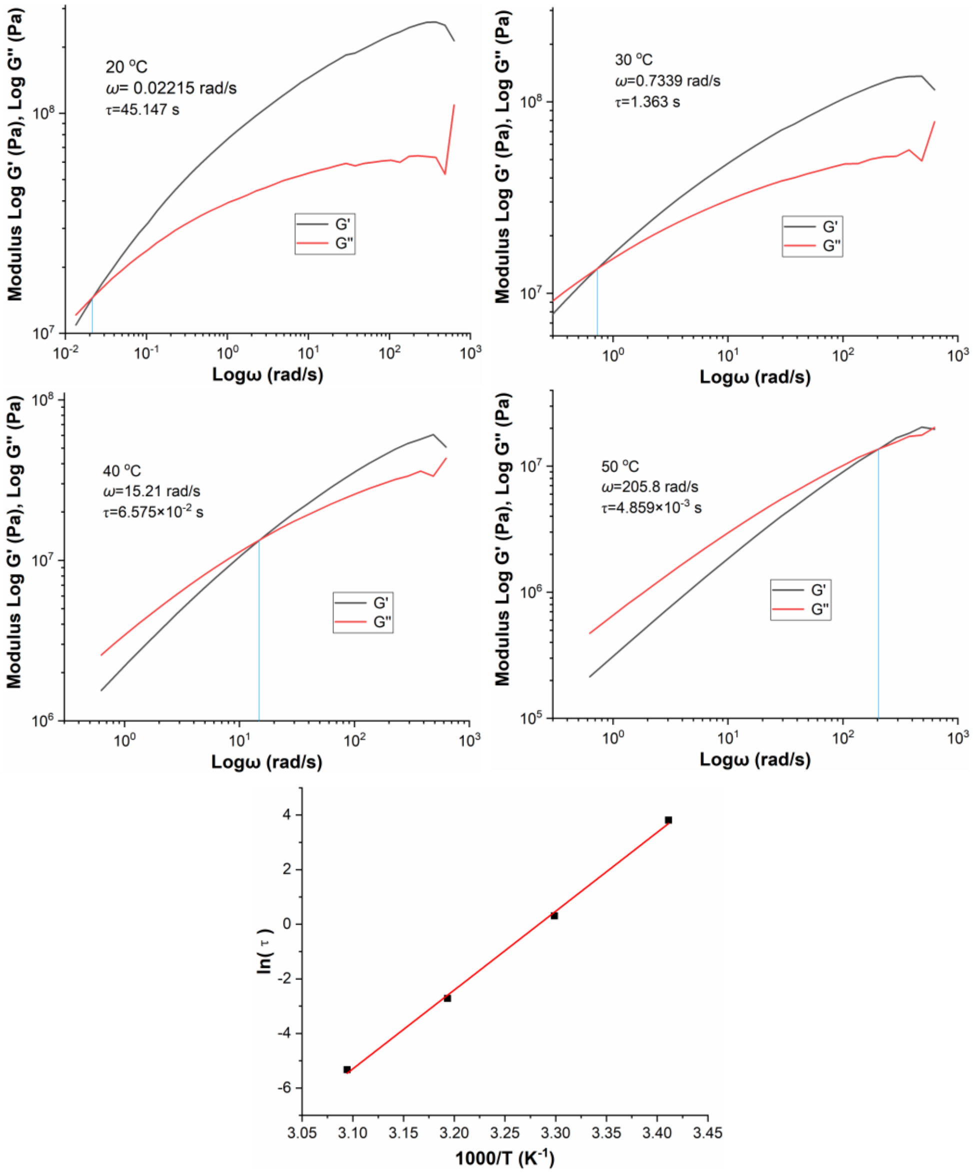

Figure S41. Temperature-dependent modulus spectra of **Sb^{V}-PNs** and Arrhenius plot ($\ln(\tau)$ *vs.* $1000/T$).

8. Self-healing

Samples of **Sb^{III}-PNs**, **Sb^{III}-PNs'**, **Sb$^{V/III}$-PNs** and **Sb^{V}-PNs** (1.0 g each) were individually dissolved in chloroform (10.0 mL), cast into separate petri dishes, and evaporated under ambient conditions. The resulting films were vacuum-dried at 50 °C for 24 h, cooled to room temperature, cutting with a razor blade, and subjected to condition-specific autonomous healing prior to optical microscopy analysis of the healed interfaces.

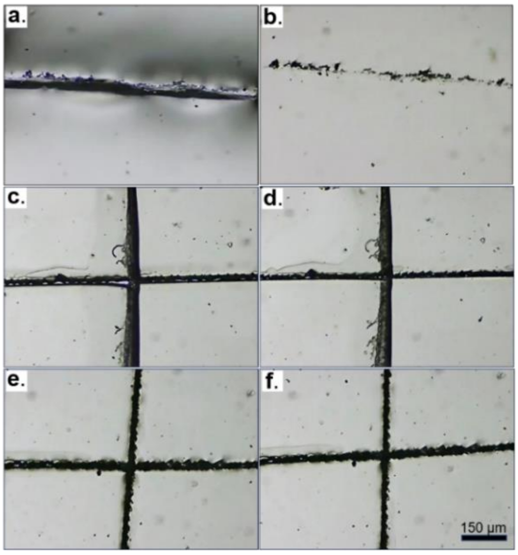

Figure S42. a) Optical micrographs of damaged film **Sb^{III}-PNs**. b) Optical micrographs of film **Sb^{III}-PNs** after repair for 5 h at room temperature. c) Optical micrographs of damaged film **Sb$^{V/III}$-PNs**. d) Optical micrographs of film **Sb$^{V/III}$-PNs** after repair for 5 h at room temperature. e) Optical micrographs of damaged film **Sb^{V}-PNs**. f) Optical micrographs of film **Sb^{V}-PNs** after repair for 5 h at room temperature.

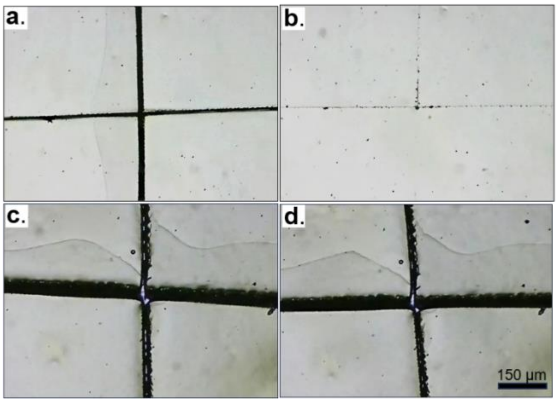

Figure S43. a) Optical micrographs of damaged film **Sb$^{V/III}$-PNs**. b) Optical micrographs of film **Sb$^{V/III}$-PNs** after repair for 20 h at room temperature. c) Optical micrographs of damaged film **Sb^{V}-PNs**. d) Optical micrographs of film **Sb^{V}-PNs** after repair for 20 h at room temperature.

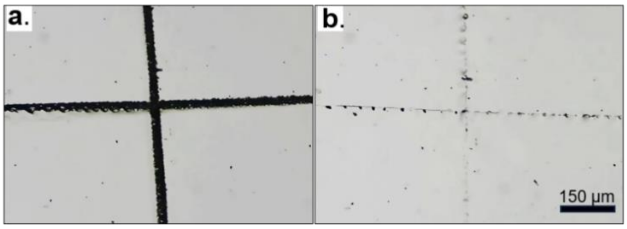

Figure S44. a) Optical micrographs of damaged film **Sb^{V}-PNs**. b) Optical micrographs of film **Sb^{V}-PNs** after repair for 24 h at 60 °C.

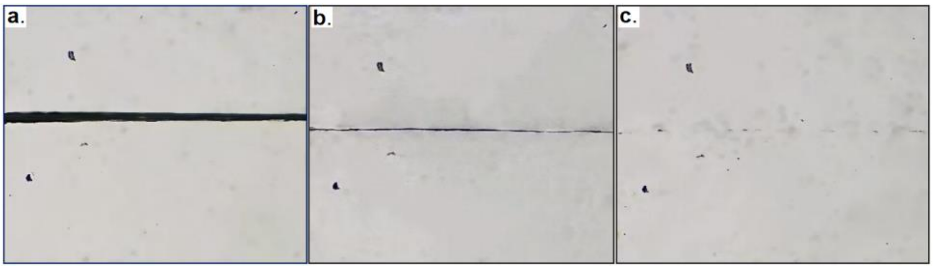

Figure S45. a) Optical micrographs of damaged film **Sb^{III}-PNs'**. b) Optical micrographs of film **Sb^{III}-PNs'** after repair for 5 h at room temperature. c) Optical micrographs of film **Sb^{III}-PNs'** after repair for 12 h at room temperature.

9. Tensile analysis

1 g of **Sb$^{V/III}$-PNs** was spread on a Teflon sheet at a thickness of 2 mm. After curing for 24 h, the sample was cut into 6 mm × 22 mm strips for further testing. Tensile experiments were performed with a strain rate of 10 mm/min at room temperature (25 °C) when evaluating the stretchability. For the self-healing samples, the strips were cut into two pieces and then put together at room temperature (25 °C) for 24 h. The healed polymer strips were then stretched following the same procedure to obtain the stress-strain curves.

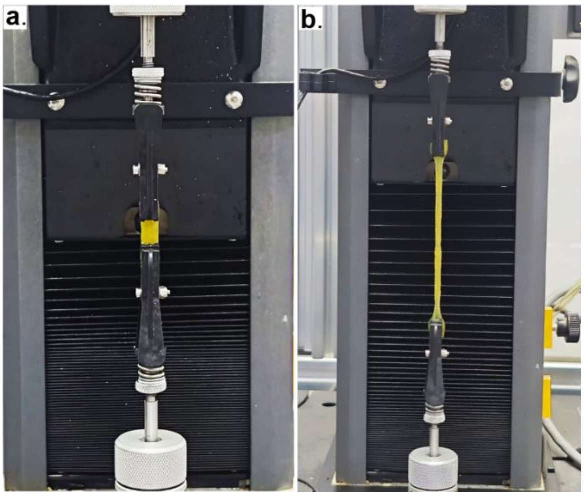

Figure S46. a) Optical images of film **Sb$^{V/III}$-PNs** before stretching. b) Optical images of film **Sb$^{V/III}$-PNs** after stretching.

1 g of **Sb$^{V/III}$-PNs** was spread on a Teflon sheet at a thickness of 2 mm. After curing for

24 h, the sample was cut into 6 mm × 22 mm strips. These strips were sectioned into two parts, promptly reassembled, and subsequently immersed in deionized water in a glass Petri dish at 25 °C for 24 h prior to testing. The healed polymer strips were then stretched following the same procedure to obtain the stress-strain curves.

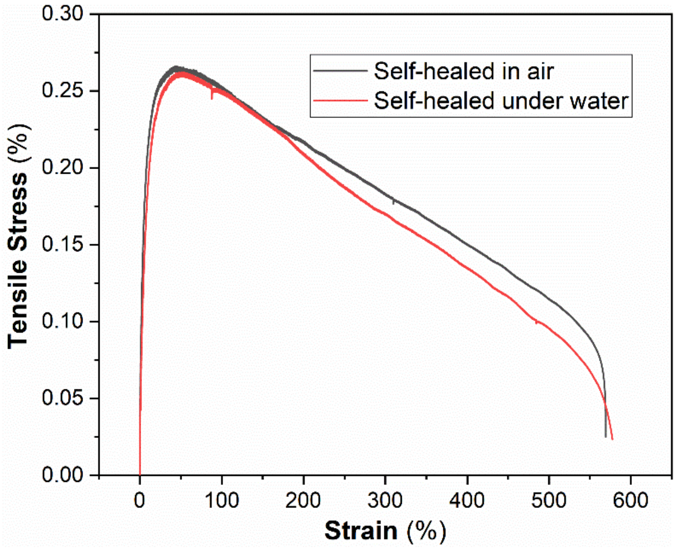

Figure S47. The stress-strain curves of **$Sb^{V/III}$-PNs** that self-healed in air and under water.

10. The cultivation of cocrystal (PnB^{V}-py)

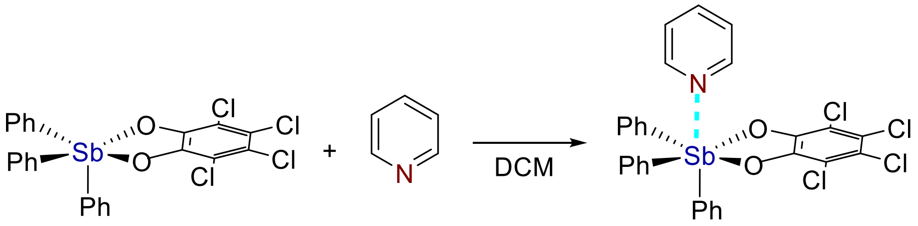

PnB^{V} **PnB^{V}**-py

Reactants **PnB^{V}** (0.6 mmol) and pyridine (1.2 mmol) were added to anhydrous DCM (40.0 mL) under nitrogen atmosphere. The above reaction mixture was stirred at room temperature for 1 h, concentrated under reduced pressure to approximately 20.0 mL and added *n*-hexane (60.0 mL) slowly. The two-phase solution was then placed for 2 h at room temperature to afford pale yellow crystals.

11. X-ray crystallographic data

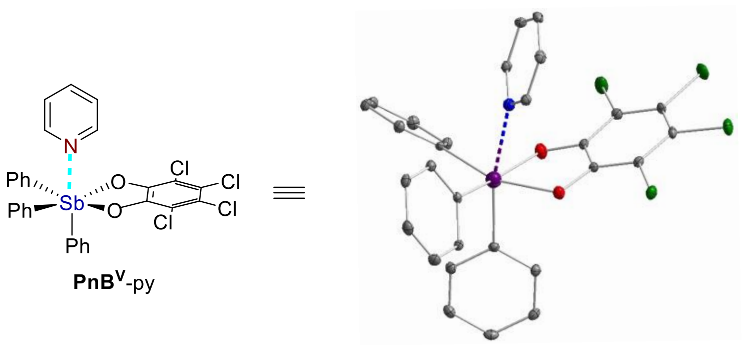

Figure S48. X-ray crystallographic structure of **PnB^{V}**-py (CCDC: 2480747).

Table S4. Crystal data and structure refinement for **PnB^{V}**-py.

Identification code	1
Empirical formula	C29H20Cl4NO2Sb
Formula weight	678.01
Temperature/K	89.9(2)
Crystal system	monoclinic
Space group	P21/n
a/Å	9.4475(2)
b/Å	21.2613(4)
c/Å	13.5360(4)
α/°	90
β/°	98.015(2)
γ/°	90
Volume/Å3	2692.36(11)
Z	4
ρcalcg/cm3	1.673
μ/mm-1	1.45
F(000)	1344
Crystal size/mm3	0.16 × 0.12 × 0.1
Radiation	Mo Kα (λ = 0.71073)
2Θ range for data collection/°	3.832 to 52.746
Index ranges	-11 ≤ h ≤ 11, -26 ≤ k ≤ 26, -16 ≤ l ≤
Reflections collected	20593
Independent reflections	5499 [Rint = 0.0309, Rsigma =
Data/restraints/parameters	5499/0/334
Goodness-of-fit on F2	1.05
Final R indexes [I>=2σ (I)]	R1 = 0.0252, wR2 = 0.0528
Final R indexes [all data]	R1 = 0.0320, wR2 = 0.0557
Largest diff. peak/hole / e Å-3	0.55/-0.33

12. Computational details

The structure of different monomers were optimized by DFT at B3LYP /def2tzvp theory of level with Grimme's dispersion having BJ-damping[8-11], using Gaussian 16 Revision A.03 program package[12]. Frequency calculations were carried out at the same level to verify all the optimized structures as ground-state structures by the absence of imaginary frequencies. The electrostatic potential (ESP) surfaces and molecular orbitals associated with pnictogen-bonding were visualized via Multiwfn Version 3.8(dev)[13-16] and VMD 1.9.3 program[17].

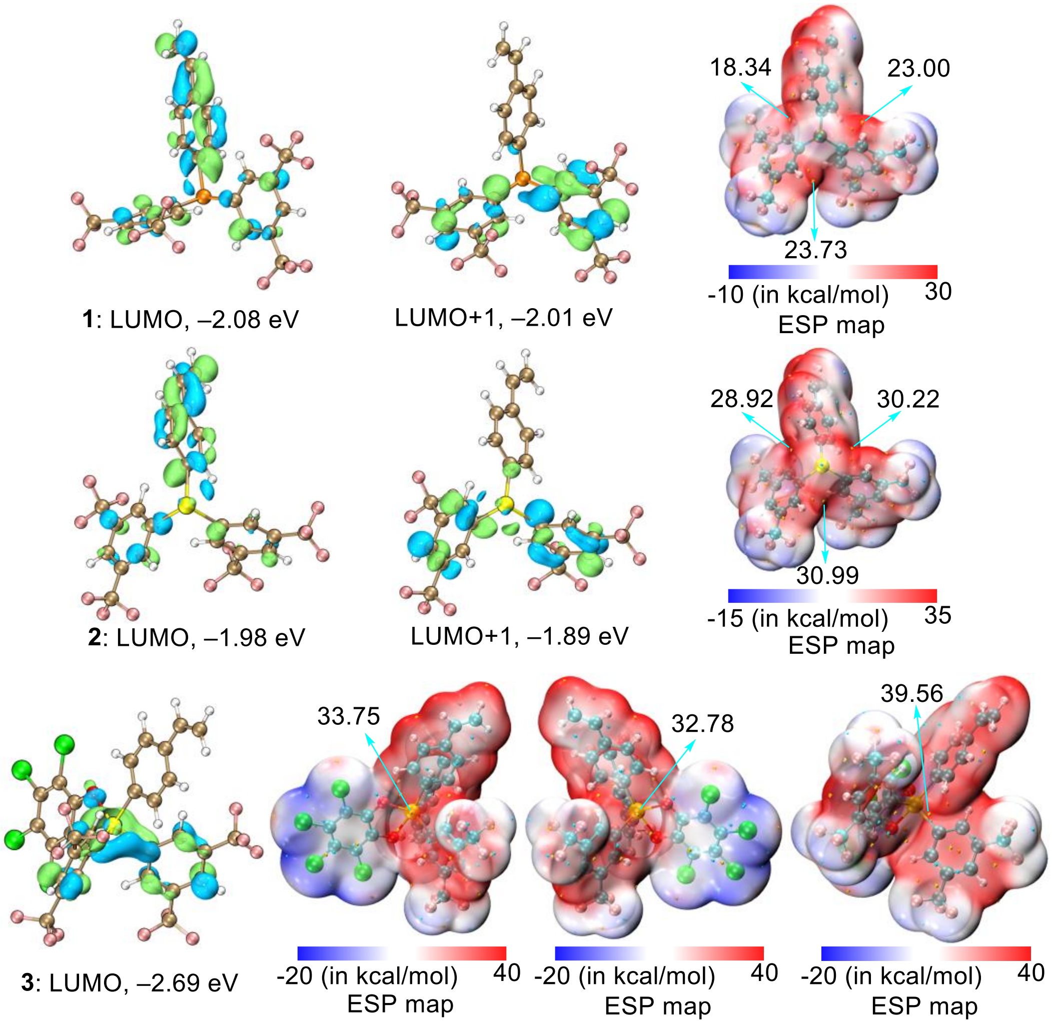

Figure S49. ESP maps and LUMO energies of monomers.

Cartesian coordinate of **1**

P	-0.25661400	0.52560800	-1.78246400
C	-1.42819900	-0.43355300	-0.73556100
C	1.35405600	0.02601700	-1.04627200

C	-0.47844100	2.21188700	-1.10986000
C	1.88434000	-1.20784900	-1.43900400
C	3.09456400	-1.64805500	-0.92919600
C	3.81689700	-0.85728900	-0.03846200
C	3.30340100	0.37218500	0.33647700
C	2.07812900	0.81379500	-0.15932700
C	-2.77164500	-0.42906800	-1.12630800
C	-3.72130400	-1.11823100	-0.39056200
C	-3.35266300	-1.84208100	0.74114300
C	-2.02246300	-1.85854800	1.12277500
C	-1.06425900	-1.15647200	0.39428100
C	-0.09427000	3.28028600	-1.92115700
C	-0.19160600	4.58555900	-1.46350100
C	-0.69243000	4.87218500	-0.18962800
C	-1.08775600	3.79516700	0.61491600
C	-0.98169900	2.49176000	0.16537100
C	-0.78389700	6.27004200	0.24128400
C	-1.27642500	6.72455900	1.39225400
C	-5.17465400	-1.06934800	-0.78205500
F	-5.34628000	-0.69278100	-2.06157000
F	-5.85914500	-0.19090900	-0.01566100
F	-5.77365600	-2.26678400	-0.62953000
C	-1.57521200	-2.62191000	2.34108200
F	-2.58245800	-3.28676900	2.93140300
F	-0.62089300	-3.52530400	2.03418700
F	-1.04193800	-1.79610200	3.26824400
C	4.05348400	1.26549700	1.28888700
F	5.16975100	0.68839200	1.76525500
F	4.42205000	2.41976700	0.69275400
F	3.29157800	1.60456600	2.35037600
C	3.67586700	-2.96569700	-1.36978700
F	2.74728400	-3.77869800	-1.90431000
F	4.63552500	-2.79142800	-2.30527200
F	4.24821400	-3.62540000	-0.34376600
H	1.34235700	-1.82912200	-2.13974500
H	4.76195800	-1.20015200	0.35547600
H	1.69046500	1.77210900	0.15633700
H	-3.07238300	0.11444400	-2.01206500
H	-4.09200100	-2.39029300	1.30564300
H	-0.03390300	-1.17802600	0.71972800
H	0.28478400	3.08789700	-2.91751800
H	0.11661700	5.40145000	-2.10566400

H	-1.47703000	3.97675800	1.60748800
H	-1.28956900	1.68316700	0.81435600
H	-0.40344500	6.99059700	-0.47615200
H	-1.67866800	6.07107400	2.15557900
H	-1.29313700	7.78398500	1.60776400

Cartesian coordinate of **2**

Sb	-0.29763300	0.49115600	-2.04114400
C	-1.61935200	-0.51626000	-0.64380700
C	1.51245200	-0.18199100	-1.04772900
C	-0.36516100	2.40274700	-1.05130200
C	1.99581300	-1.46047700	-1.32804000
C	3.13250300	-1.93994000	-0.69042100
C	3.81467800	-1.14638800	0.22624900
C	3.34255300	0.12781400	0.49644300
C	2.19763400	0.61032000	-0.13514800
C	-2.97972700	-0.57570200	-0.94249100
C	-3.86867900	-1.19074300	-0.06870900
C	-3.41394600	-1.75963200	1.11500700
C	-2.05998100	-1.70861100	1.41031300
C	-1.16689900	-1.09067800	0.53901200
C	0.09950900	3.52111500	-1.74056300
C	0.12667900	4.76690700	-1.12805800
C	-0.31561600	4.93753200	0.18716000
C	-0.78955500	3.80943000	0.86924500
C	-0.81434900	2.56579900	0.26152500
C	-0.26533300	6.27437200	0.78689200
C	-0.64791100	6.61904300	2.01511300
C	-5.34132900	-1.20053300	-0.38207900
F	-5.57467600	-1.22503000	-1.70854000
F	-5.95445200	-0.09714900	0.10244100
F	-5.96685000	-2.26370500	0.15674000
C	-1.53565500	-2.28324900	2.69939200
F	-2.41734900	-3.10924700	3.28945600
F	-0.39647600	-2.97825600	2.50798700
F	-1.24937200	-1.30652500	3.58979100
C	4.04276400	1.01800900	1.48859500
F	5.14459800	0.44744000	2.00588400
F	4.42242300	2.18341400	0.92260000
F	3.23328000	1.33560900	2.52177800
C	3.66660100	-3.30718400	-1.02548800
F	2.69835100	-4.13596600	-1.46005400

F	4.59824000	-3.24565900	-2.00358600
F	4.25304600	-3.89318100	0.03529000
H	1.48328100	-2.09977900	-2.03564400
H	4.69638600	-1.52079400	0.72417900
H	1.84216400	1.60459500	0.10057000
H	-3.36008600	-0.14883200	-1.86191100
H	-4.10337300	-2.24588700	1.78867700
H	-0.11627800	-1.06599900	0.79370400
H	0.44835700	3.43074100	-2.76297300
H	0.49578000	5.62513100	-1.67660700
H	-1.14089300	3.90190700	1.88815100
H	-1.18393900	1.71687300	0.82139700
H	0.13231600	7.04722100	0.13617000
H	-1.05377300	5.90786800	2.72293400
H	-0.56368200	7.64206400	2.35495500

Cartesian coordinate of **3**

C	0.78552400	-1.78773200	-0.57423000
C	1.83668900	-2.57812200	-0.13192400
C	2.16115400	-3.74525300	-0.81990800
C	1.44439500	-4.12012300	-1.94400900
C	0.38693400	-3.32492200	-2.37711200
C	0.04627300	-2.16605800	-1.69534900
Sb	0.29916200	0.00930000	0.47836500
O	-1.60717700	-0.13449400	-0.36302800
O	-0.86146100	0.01727600	2.12664200
C	2.00190900	0.14086900	1.77013300
C	0.58102300	1.78553000	-0.67786100
C	1.55326500	2.70195700	-0.30119500
C	1.74211000	3.85755200	-1.05485400
C	0.96906100	4.09726400	-2.17952700
C	-0.00771900	3.17659300	-2.54622000
C	-0.21334000	2.02582600	-1.79849600
C	1.89575100	0.19093400	3.15678600
C	3.04511200	0.27958400	3.93149300
C	4.31823000	0.32060800	3.35480300
C	4.40821600	0.27024600	1.95730600
C	3.26853000	0.18164300	1.17848400
C	-4.49605700	-0.09395800	2.58252000
C	-3.13242500	-0.02870600	2.90341100
C	-2.18910300	-0.04401100	1.88865800
C	-2.58366600	-0.12404800	0.54558800

C	-3.93405700	-0.18694700	0.22493500
C	-4.89444000	-0.17252000	1.24557900
Cl	-4.37169200	-0.28091100	-1.44508200
Cl	-6.57080400	-0.25214800	0.83895200
Cl	-5.67240400	-0.07584600	3.84693300
Cl	-2.58387000	0.07162100	4.54030200
C	5.49197000	0.41365400	4.22819600
C	6.76870100	0.45529400	3.85223300
C	-0.42030500	-3.76534800	-3.57226700
F	-1.33947100	-4.69070400	-3.22675100
F	0.36135900	-4.32295600	-4.51818700
F	-1.07818500	-2.74236400	-4.14166200
C	3.32236000	-4.56944200	-0.32822100
F	4.48940100	-3.90422000	-0.47758100
F	3.43957700	-5.73376400	-0.98626400
F	3.20367700	-4.85376900	0.98391000
C	2.82604700	4.81893900	-0.64190500
F	2.76984000	5.08914200	0.67691500
F	4.05210100	4.30241600	-0.88137300
F	2.75114700	5.98741400	-1.29923600
C	-0.88408900	3.46466600	-3.73923100
F	-0.20993500	4.12206300	-4.70285400
F	-1.93606900	4.23718800	-3.39735800
F	-1.37648300	2.33980400	-4.28434900
H	2.40729100	-2.30197900	0.74399600
H	1.70285600	-5.02157900	-2.47973000
H	-0.79467100	-1.57319000	-2.02106500
H	2.16395100	2.53299600	0.57481000
H	1.12203200	4.99144800	-2.76544400
H	-0.99352300	1.33225900	-2.07349500
H	0.92549100	0.16137900	3.62978600
H	2.95409700	0.31788100	5.01008200
H	5.37442000	0.30054500	1.47291900
H	3.37738500	0.14445200	0.10031100
H	5.26484600	0.45015500	5.28895800
H	7.55990300	0.52430900	4.58587500
H	7.07583500	0.42256300	2.81486700

13. Copies of NMR spectra and GPC spectra

^{1}H NMR spectrum of compound **1** ($CDCl_3$, 400 MHz)

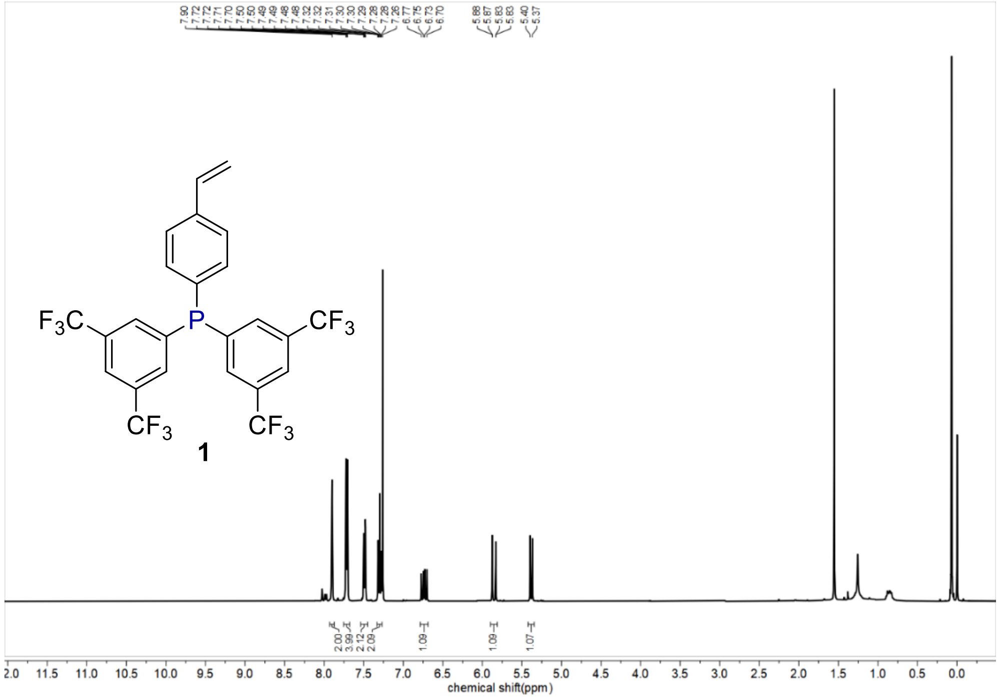

^{13}C NMR spectrum of compound **1** ($CDCl_3$, 100 MHz)

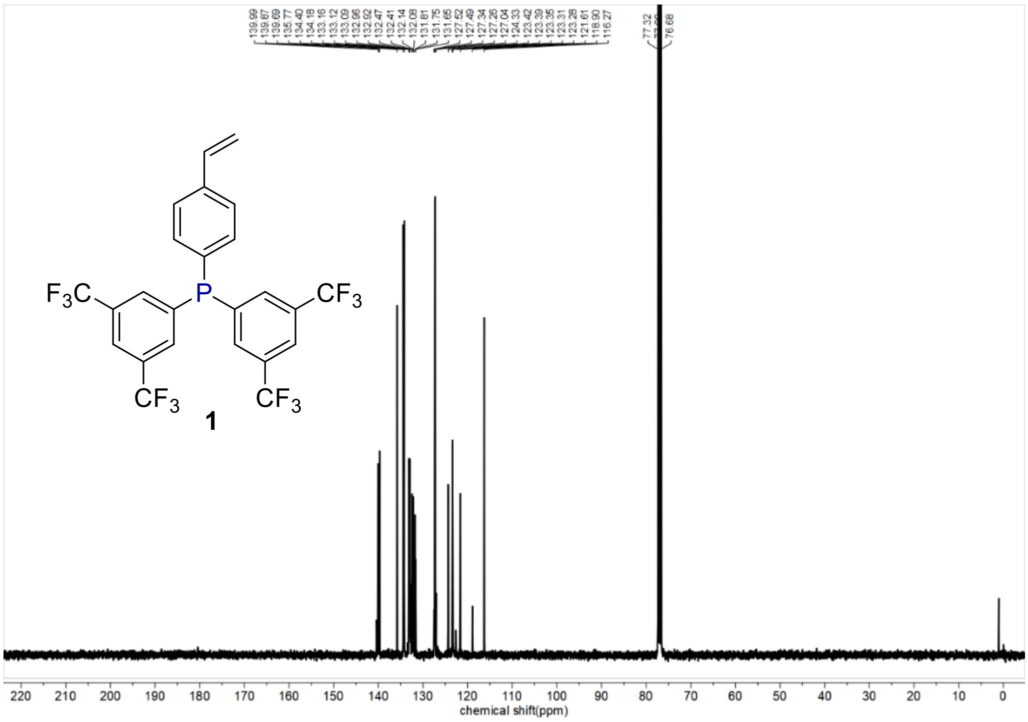

^{13}C NMR spectrum of compound **1** ($CDCl_3$, 100 MHz)

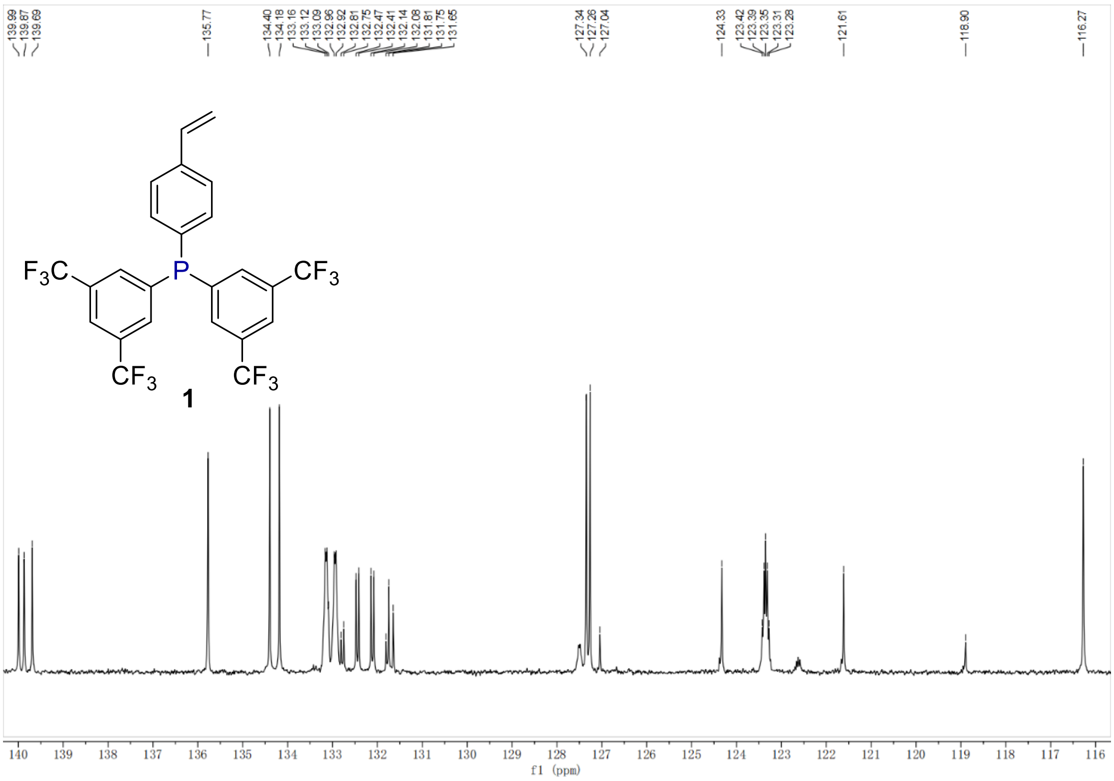

^{19}F NMR spectrum of compound **1** ($CDCl_3$, 376 MHz)

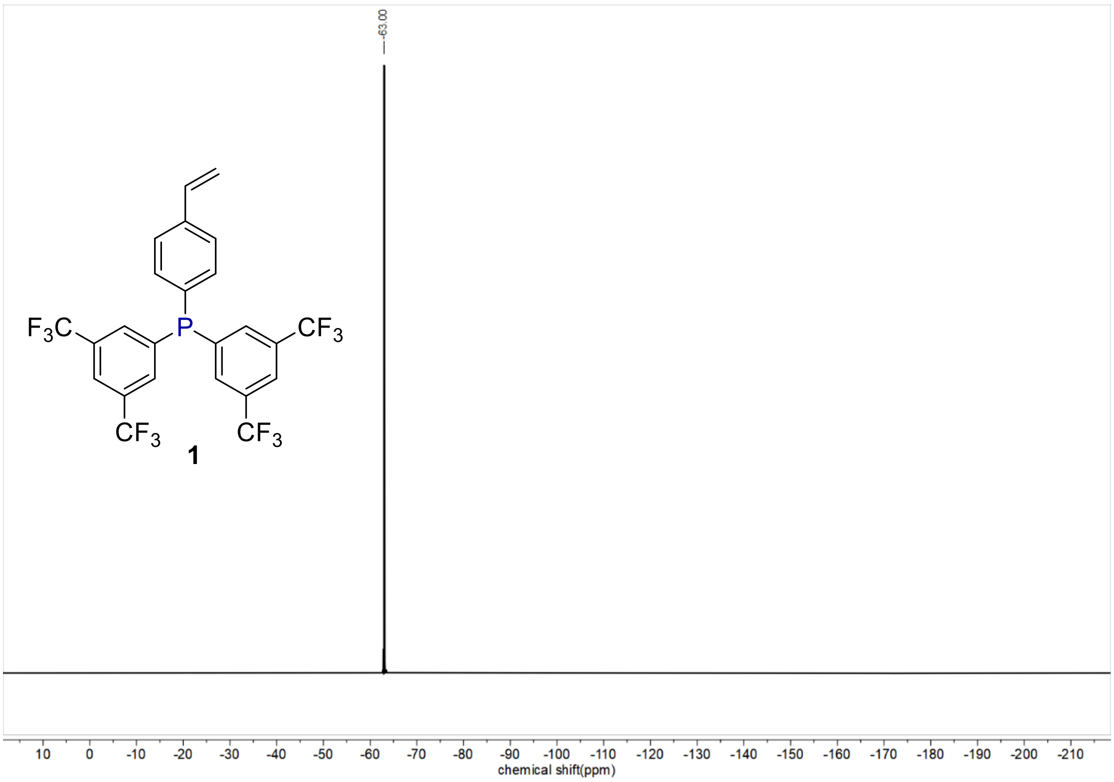

^{31}P NMR spectrum of compound **1** ($CDCl_3$, 162 MHz)

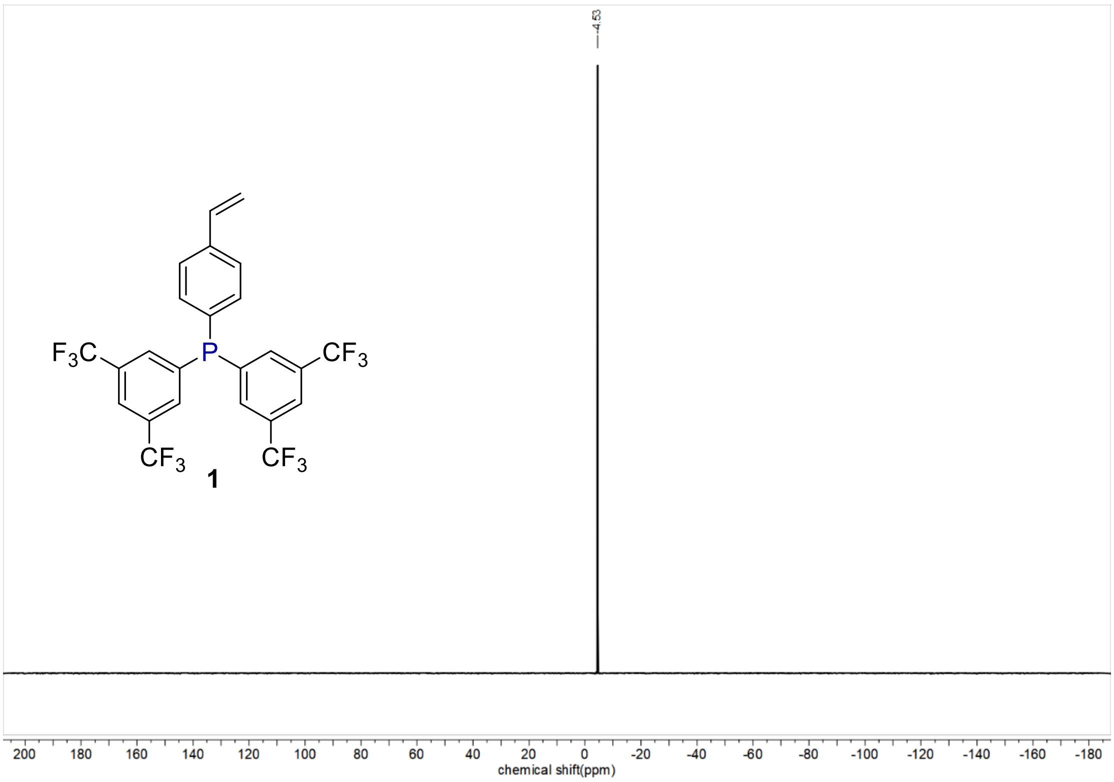

^{1}H NMR spectrum of compound **M1** ($CDCl_3$, 400 MHz)

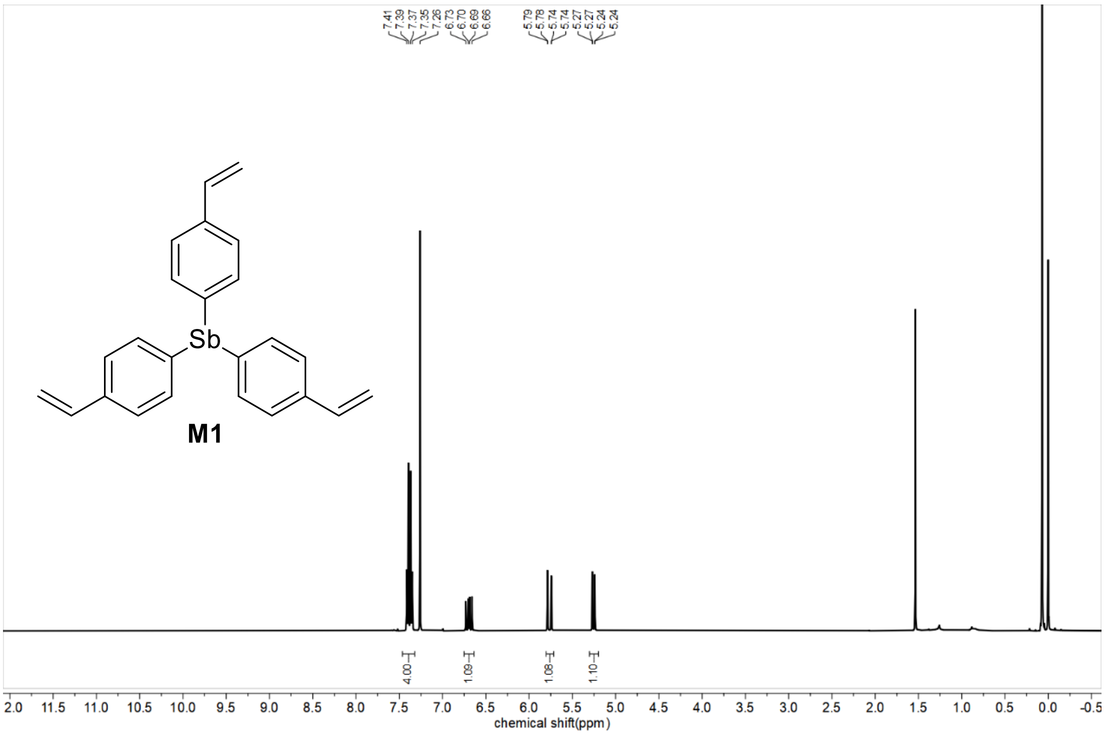

^{13}C NMR spectrum of compound **M1** ($CDCl_3$, 100 MHz)

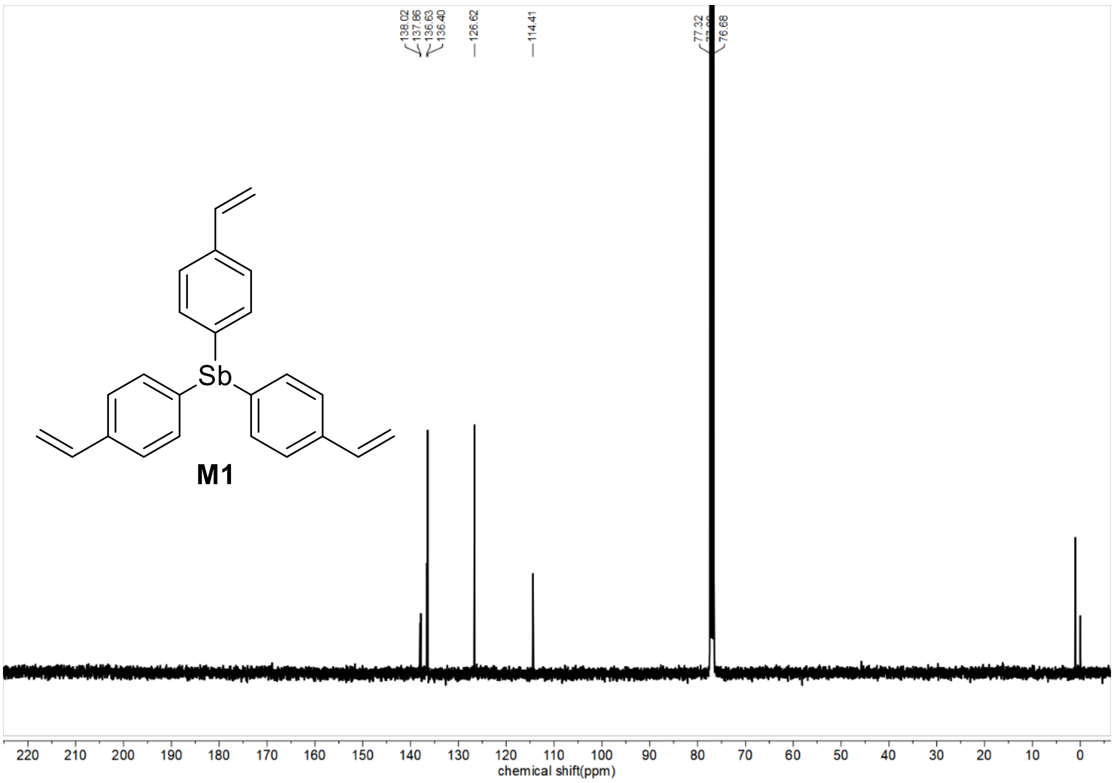

^{1}H NMR spectrum of compound **2** ($CDCl_3$, 400 MHz)

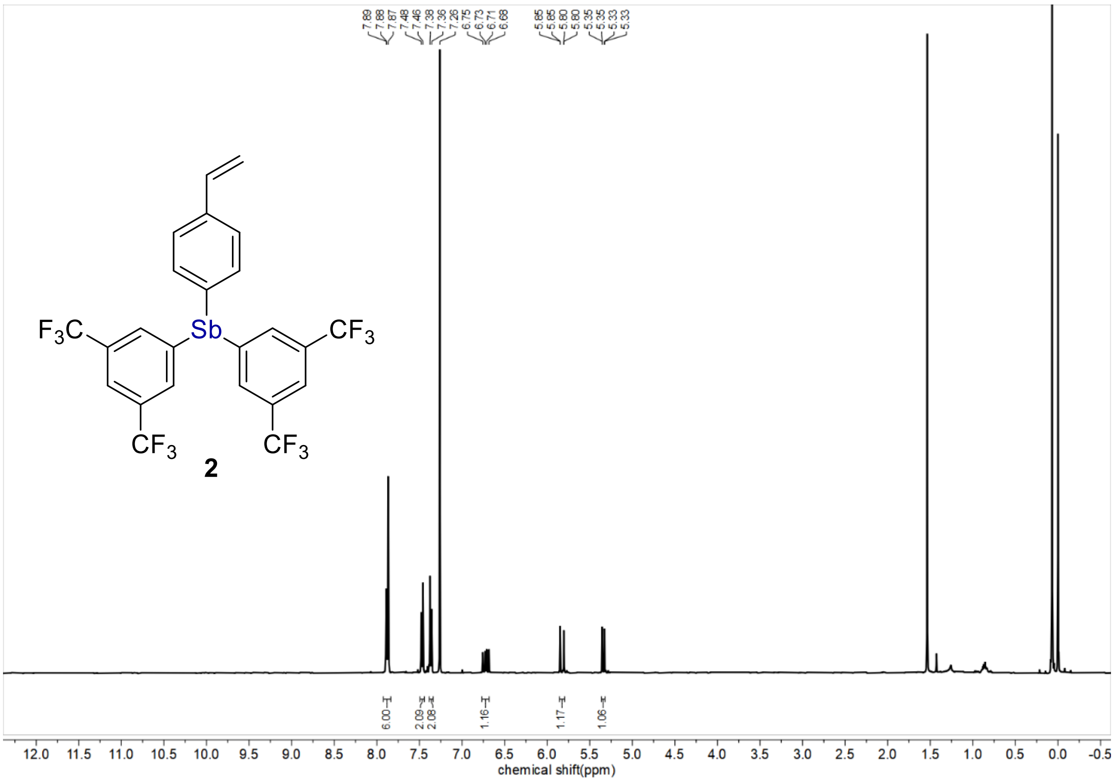

^{13}C NMR spectrum of compound **2** ($CDCl_3$, 100 MHz)

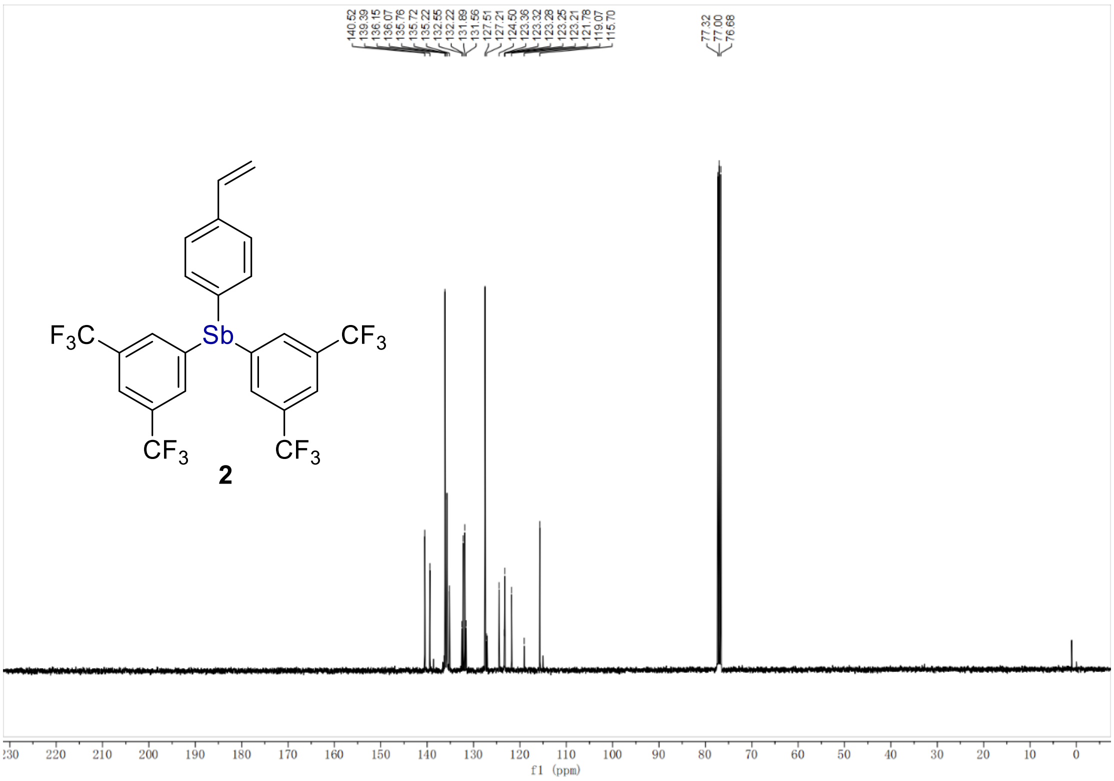

^{13}C NMR spectrum of compound **2** ($CDCl_3$, 100 MHz)

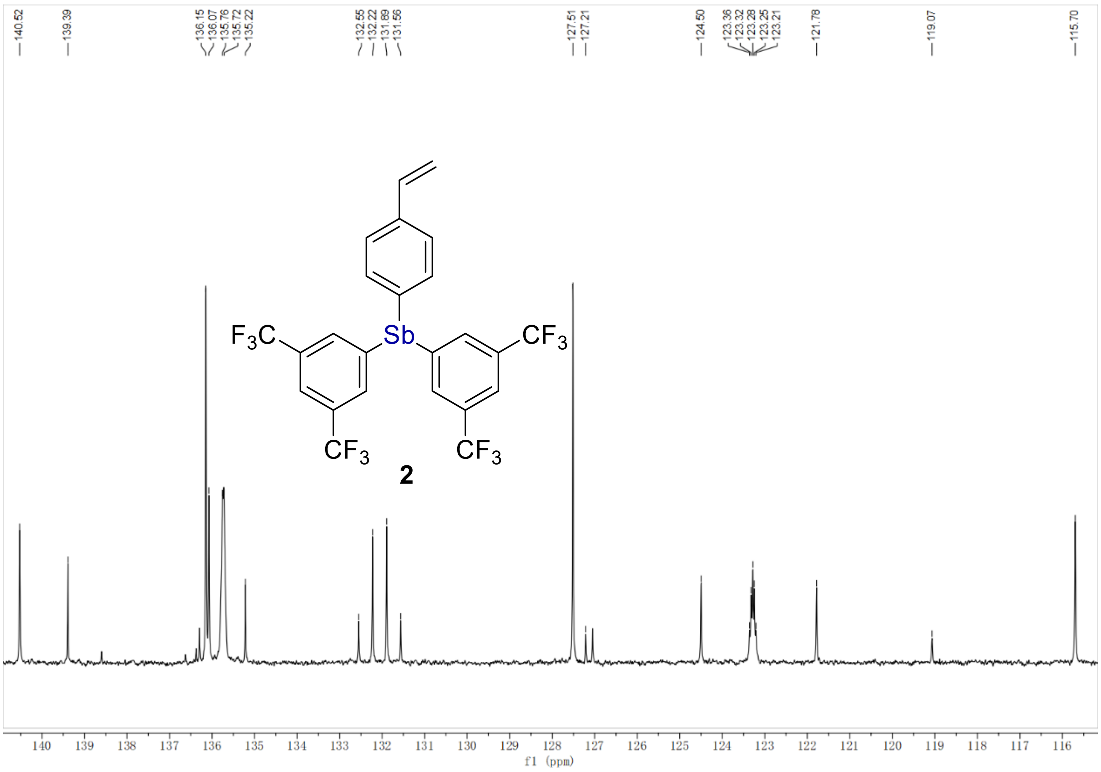

^{19}F NMR spectrum of compound **2** ($CDCl_3$, 376 MHz)

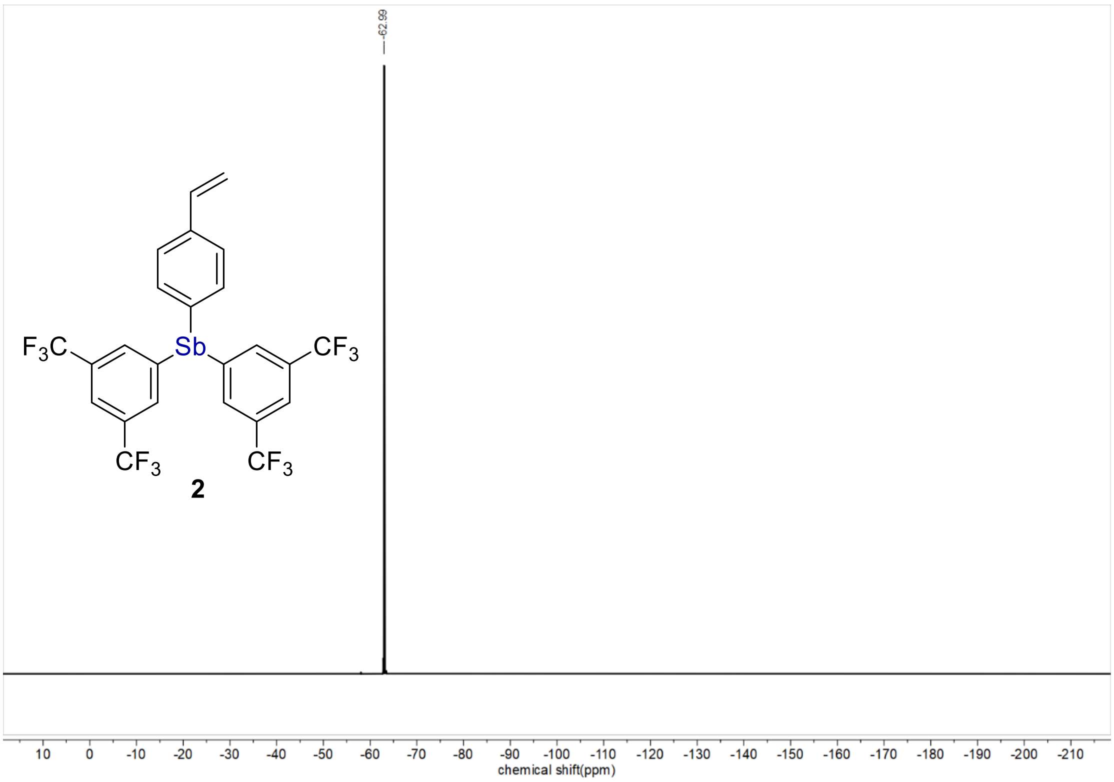

^{1}H NMR spectrum of compound **3** ($CDCl_3$, 400 MHz)

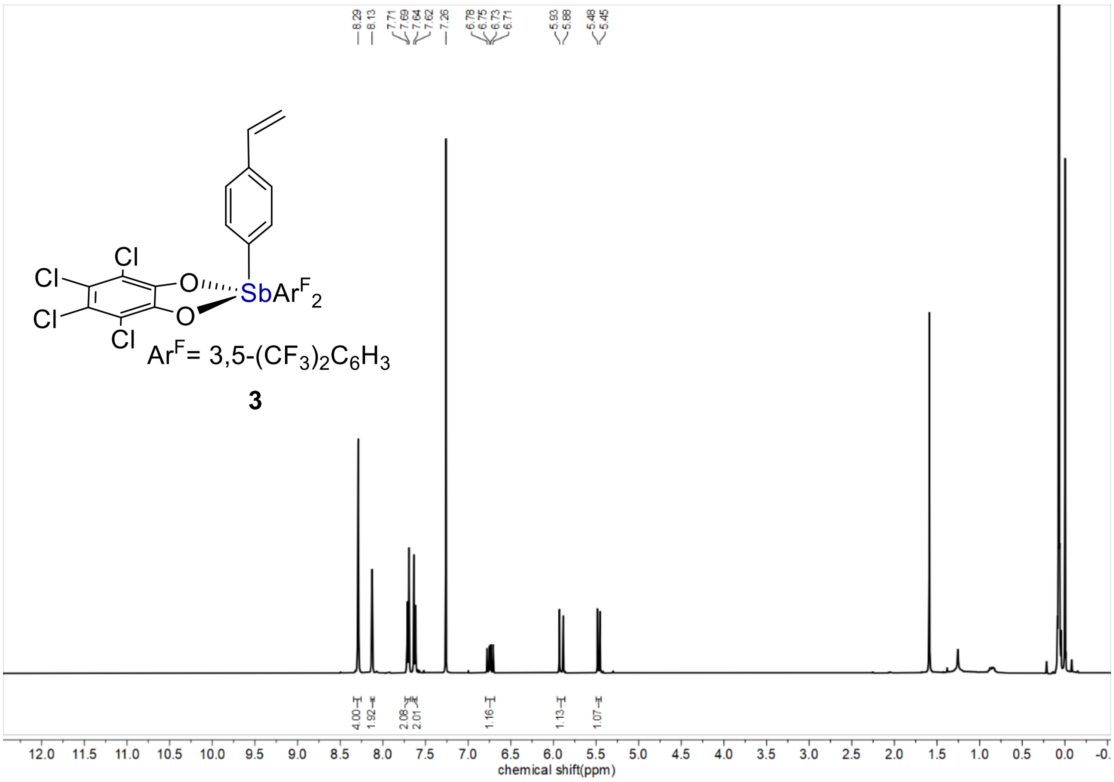

^{13}C NMR spectrum of compound **3** ($CDCl_3$, 100 MHz)

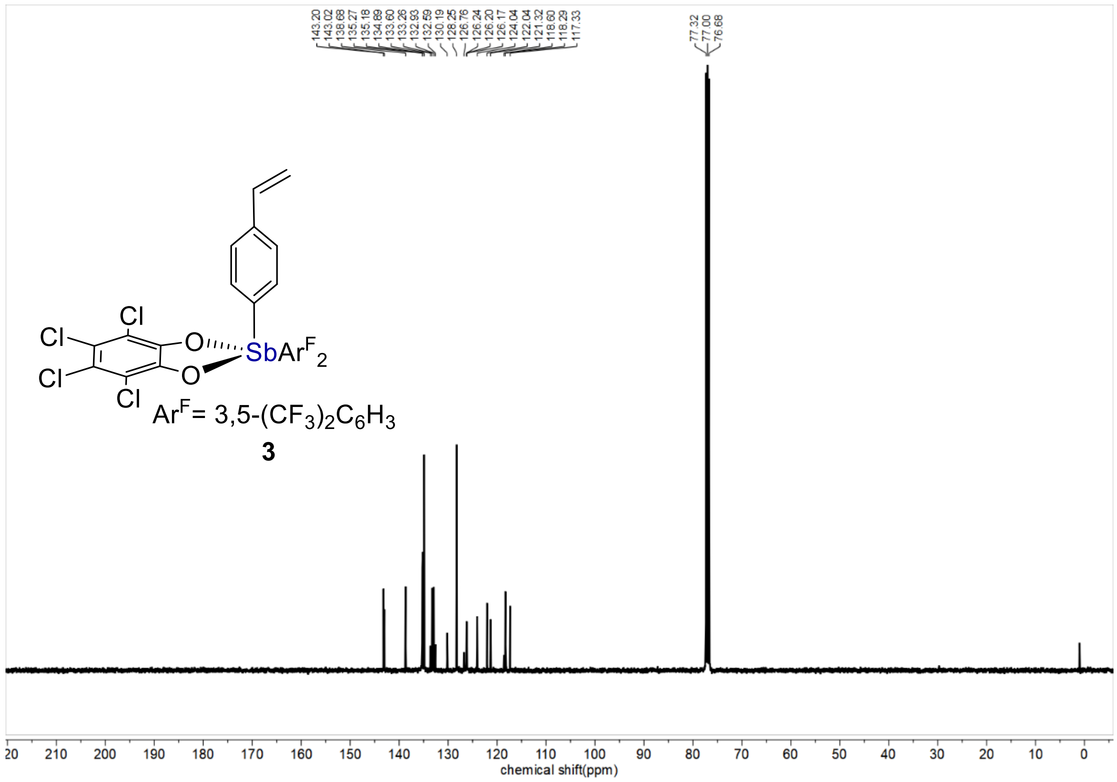

^{13}C NMR spectrum of compound **3** ($CDCl_3$, 100 MHz)

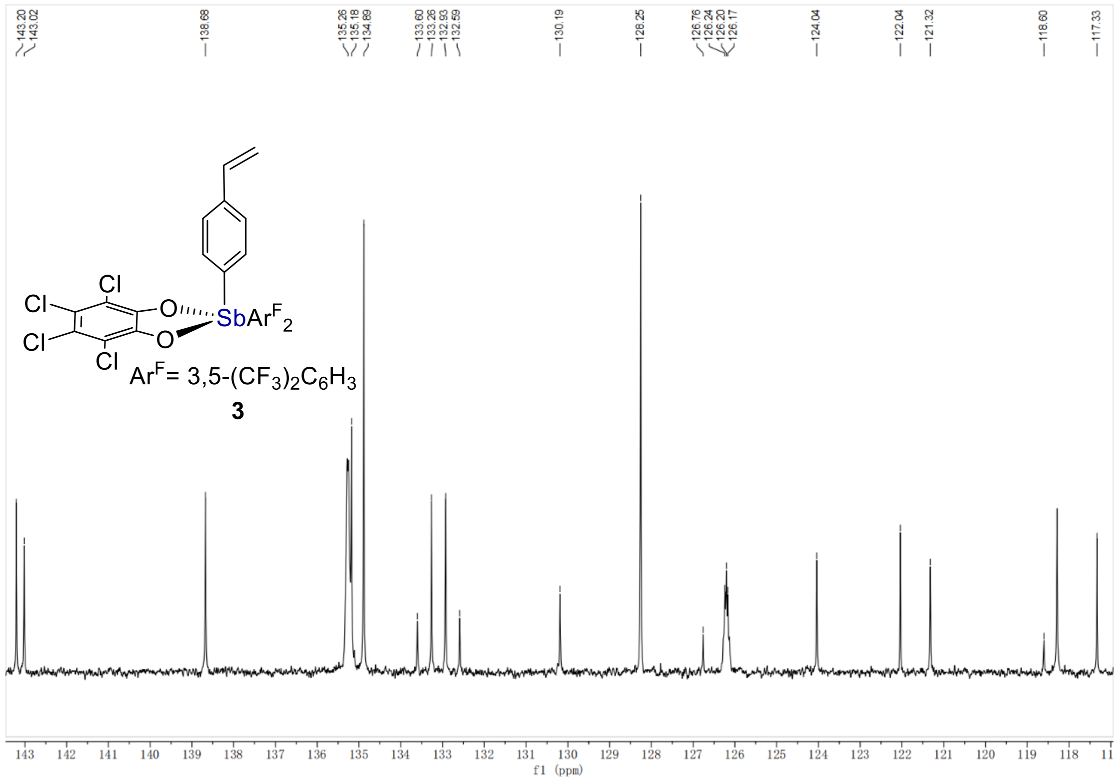

^{19}F NMR spectrum of compound **3** ($CDCl_3$, 376 MHz)

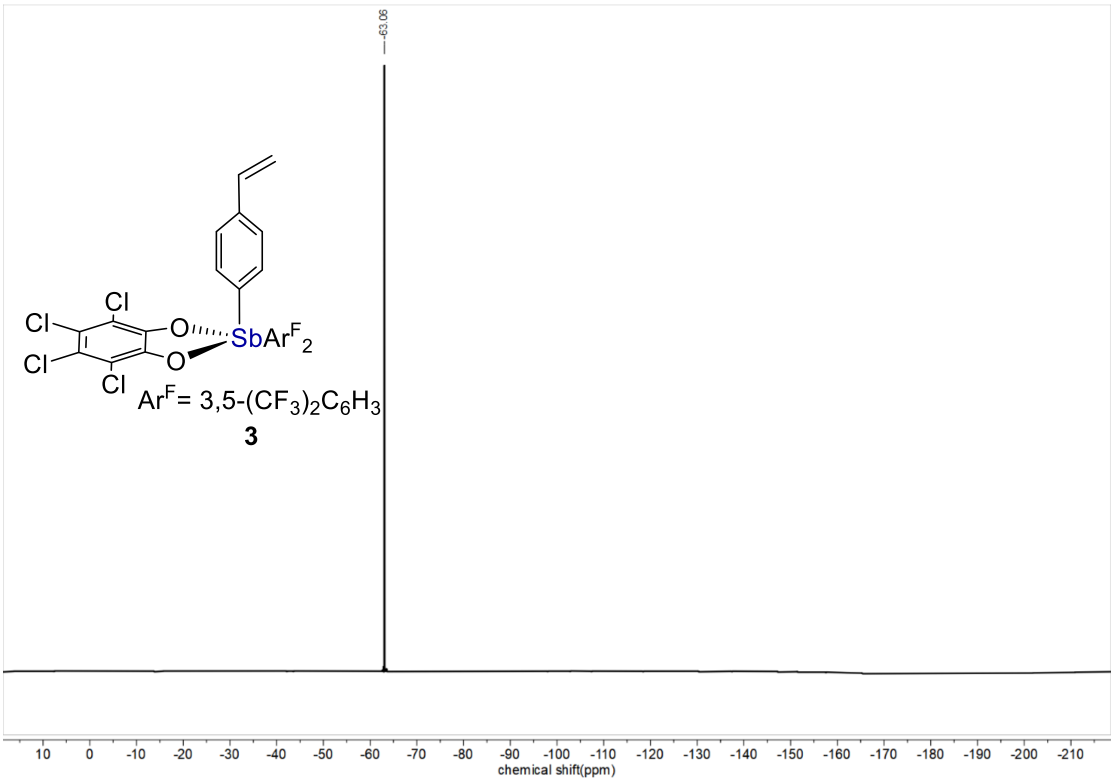

^{1}H NMR spectrum of compound **M2** ($CDCl_3$, 400 MHz)

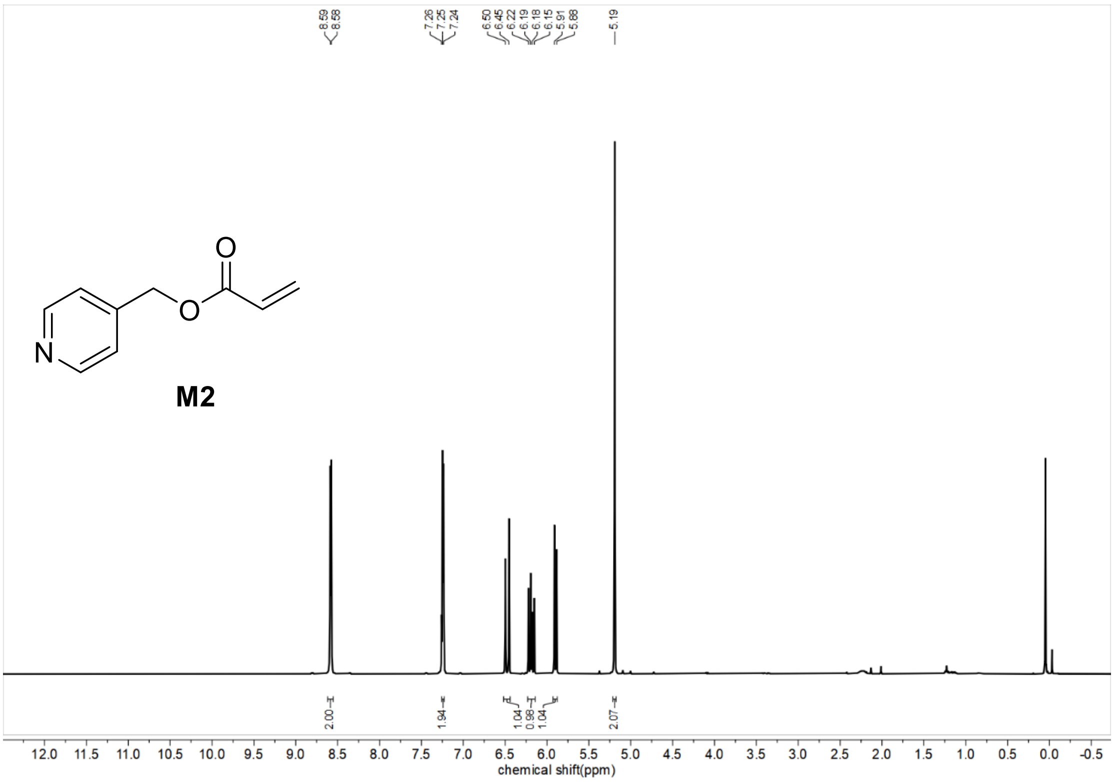

^{13}C NMR spectrum of compound **M2** ($CDCl_3$, 100 MHz)

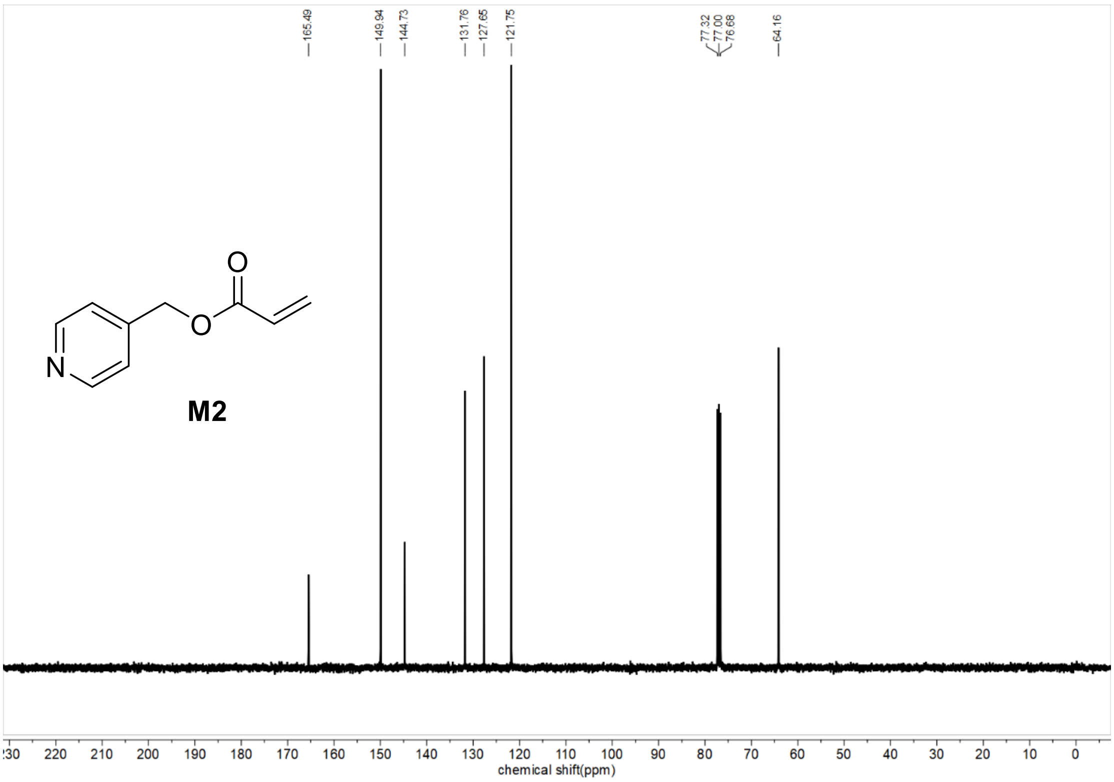

^{1}H NMR spectrum of compound **M3** ($CDCl_3$, 400 MHz)

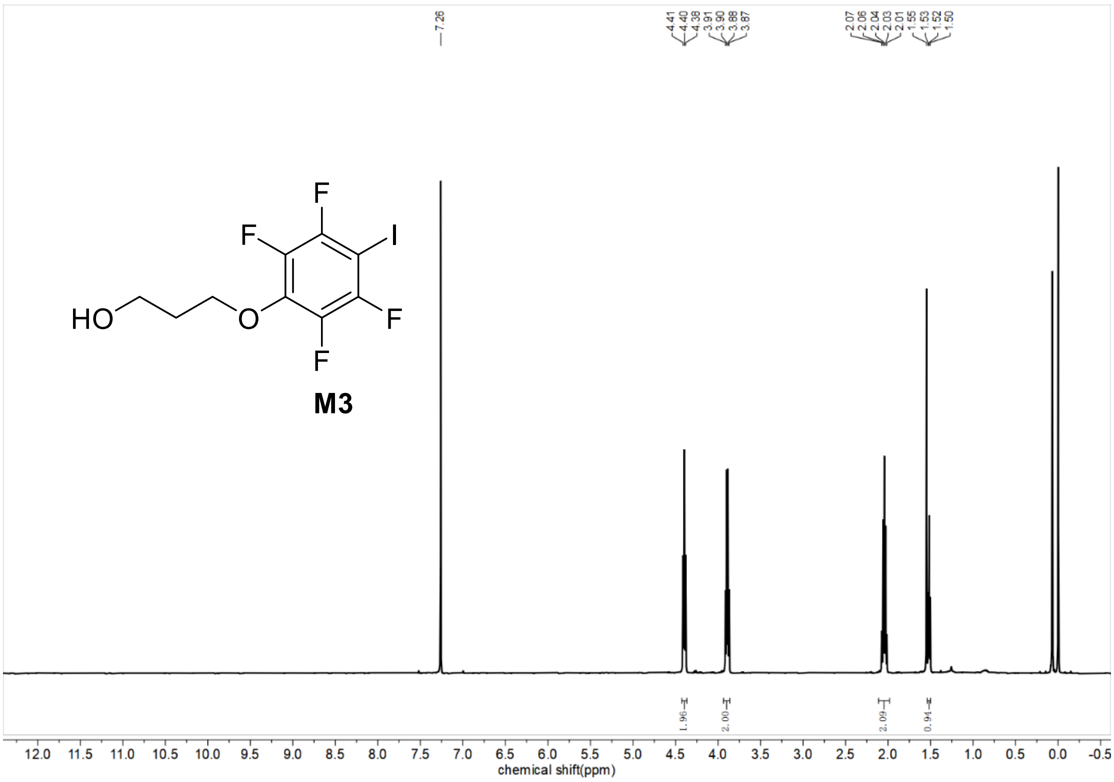

^{13}C NMR spectrum of compound **M3** ($CDCl_3$, 100 MHz)

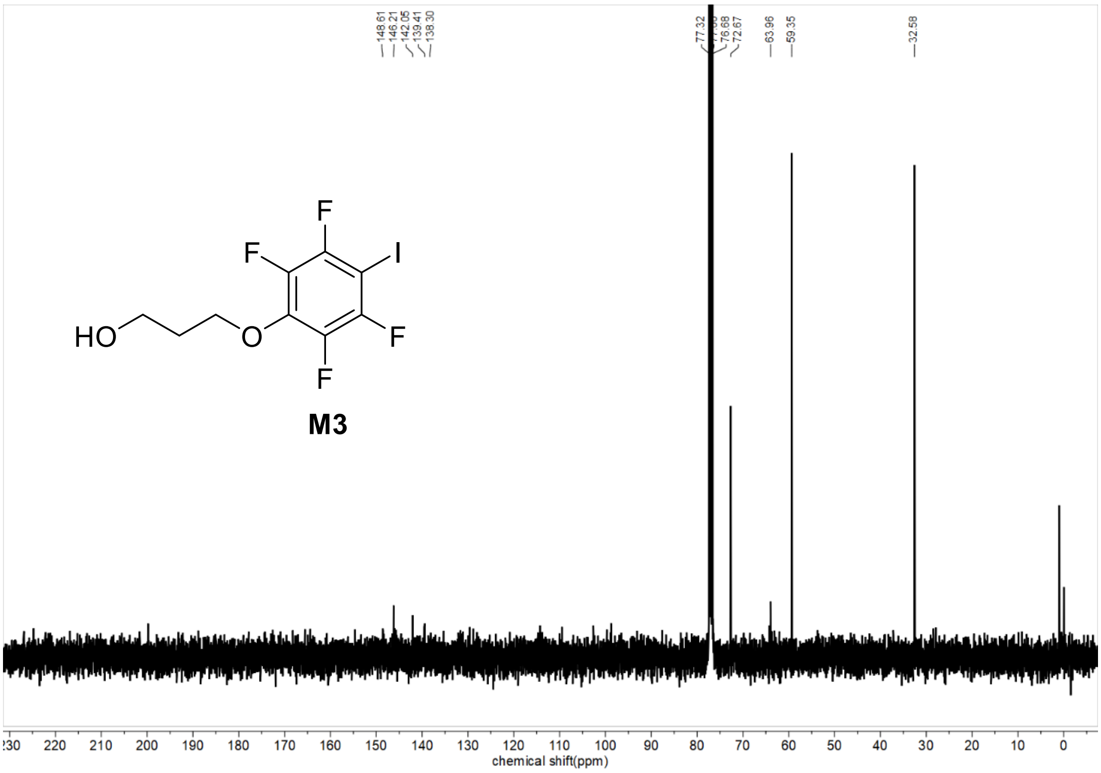

^{19}F NMR spectrum of **M3** ($CDCl_3$, 376 MHz)

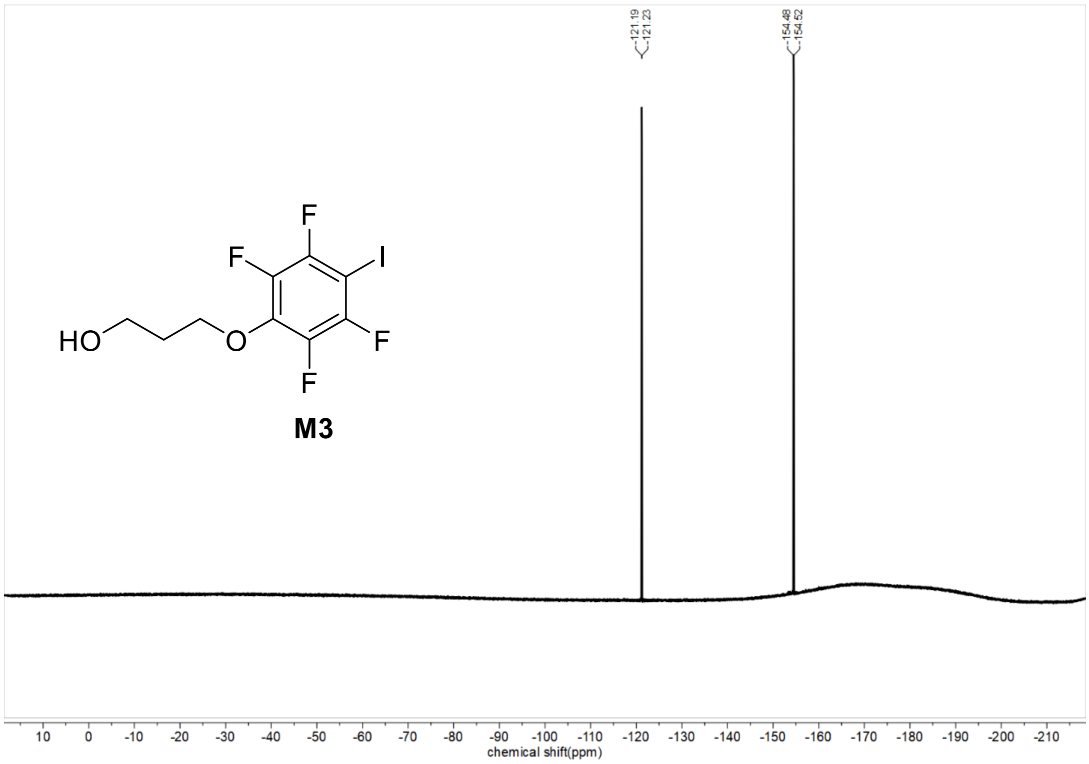

^{1}H NMR spectrum of compound **M4** ($CDCl_3$, 400 MHz)

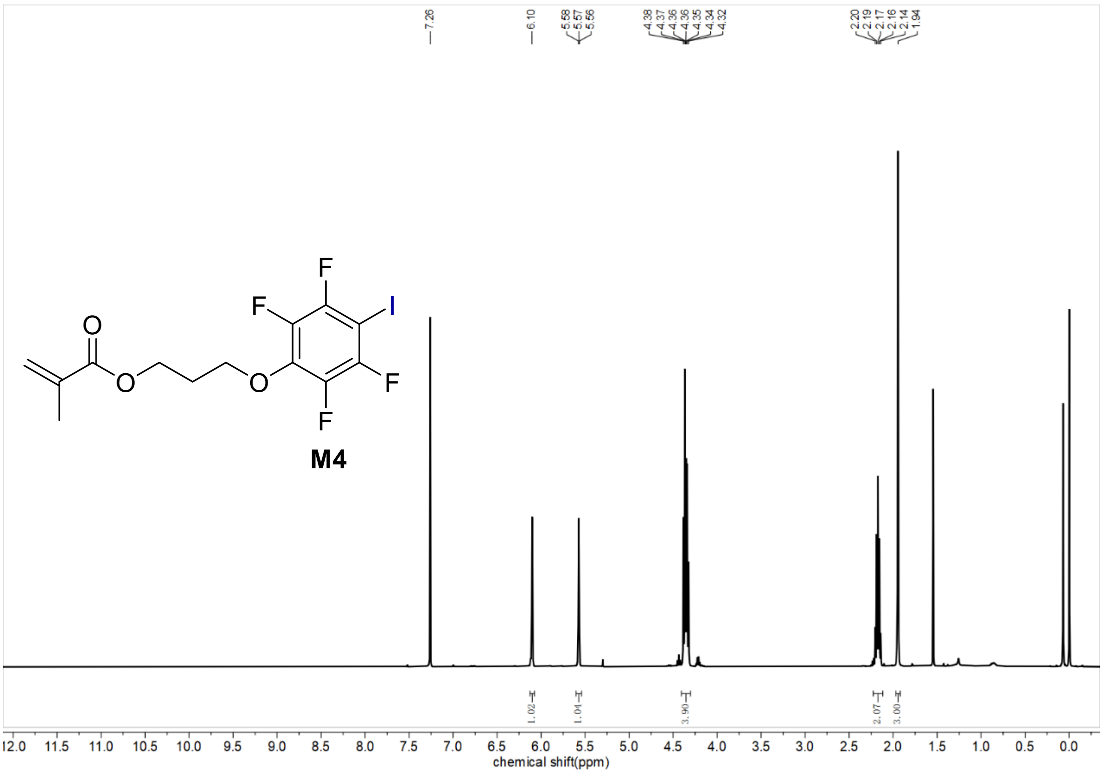

^{13}C NMR spectrum of compound **M4** ($CDCl_3$, 100 MHz)

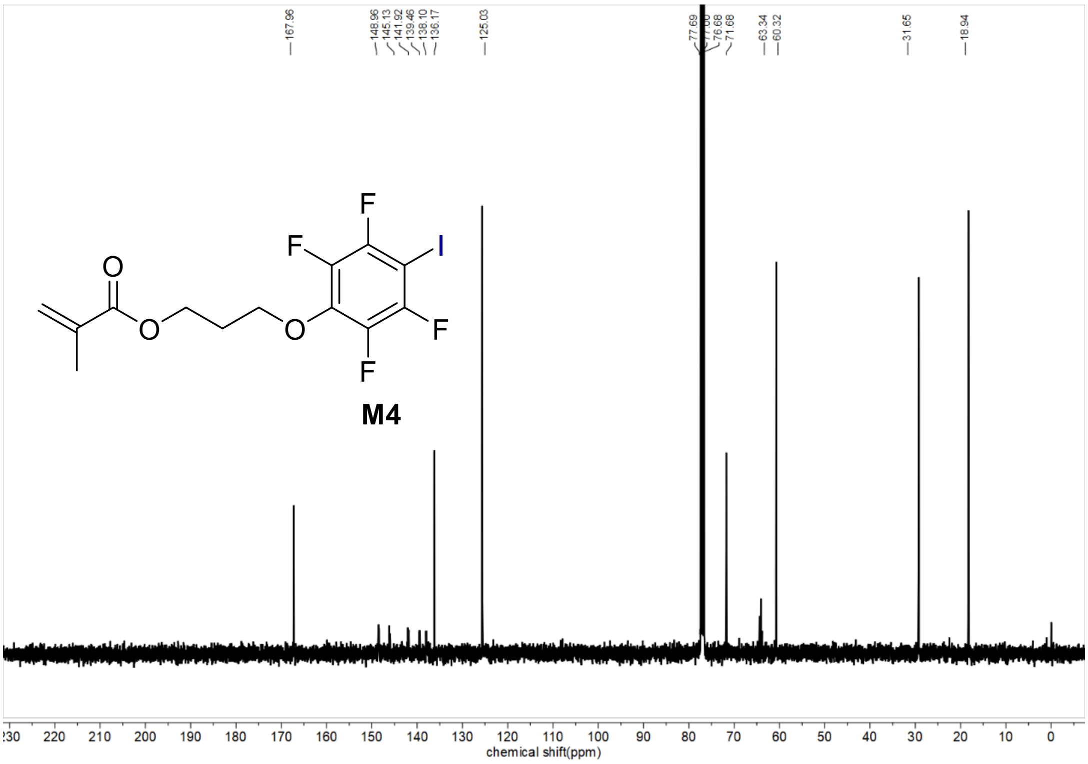

^{19}F NMR spectrum of **M4** ($CDCl_3$, 376 MHz)

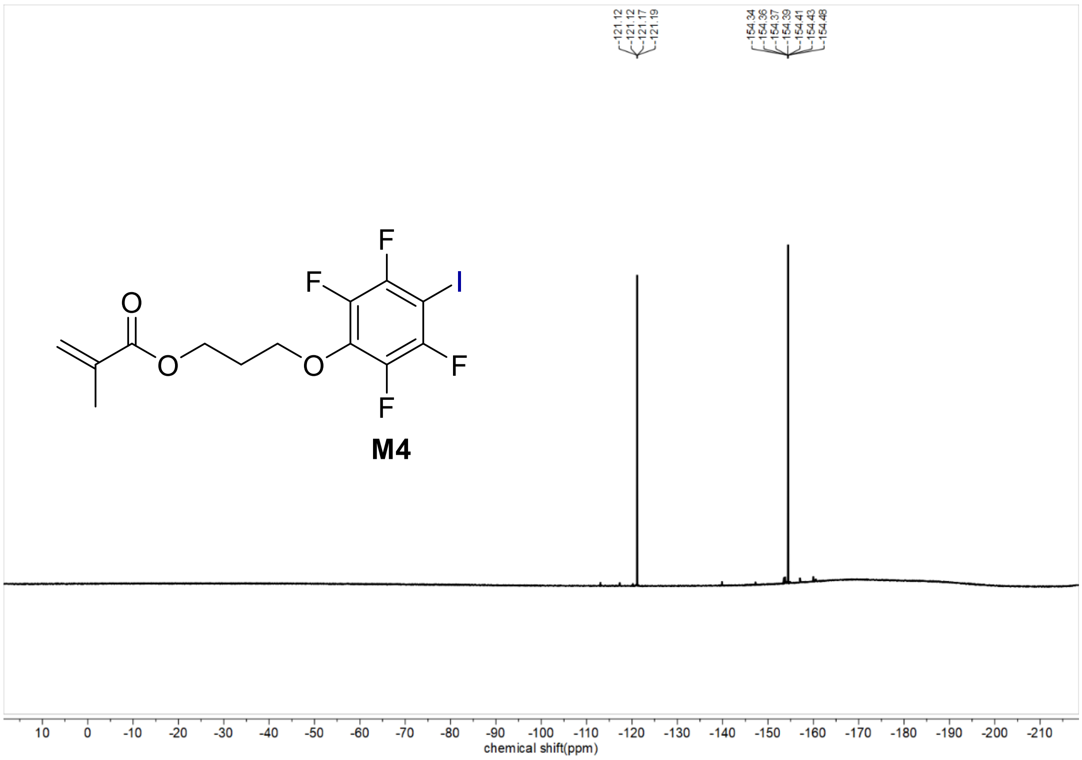

^{1}H NMR spectrum of compound **M5** ($CDCl_3$, 400 MHz)

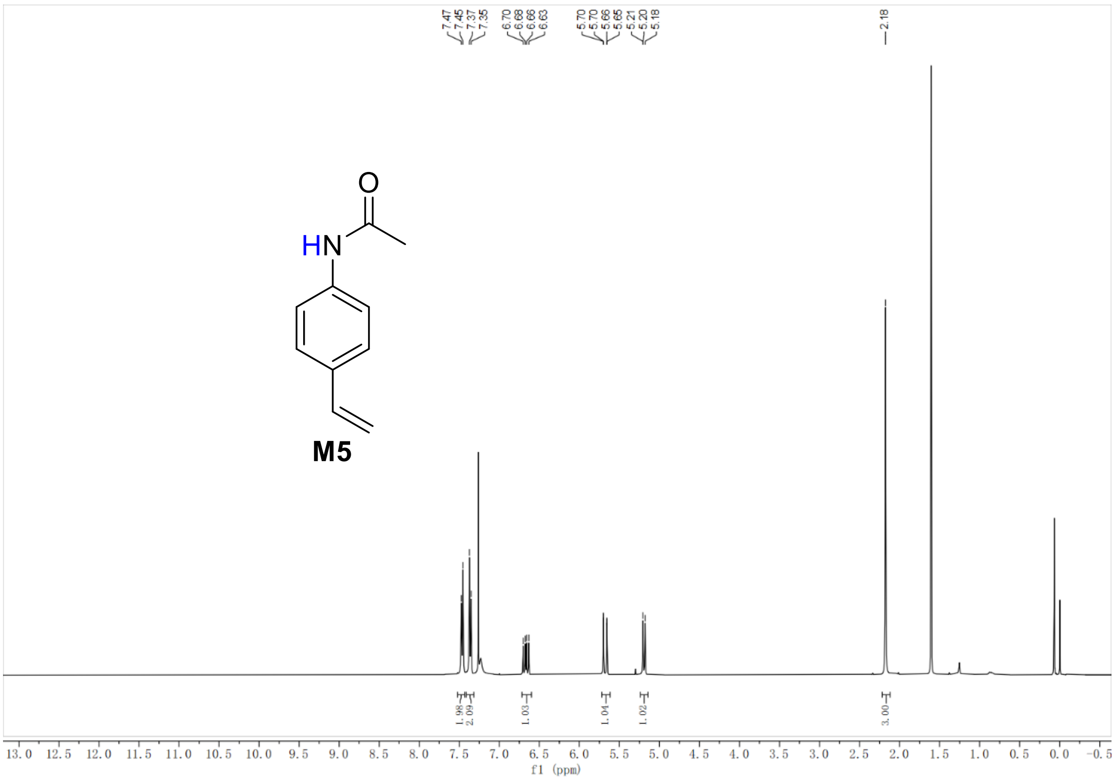

^{13}C NMR spectrum of compound **M5** ($CDCl_3$, 100 MHz)

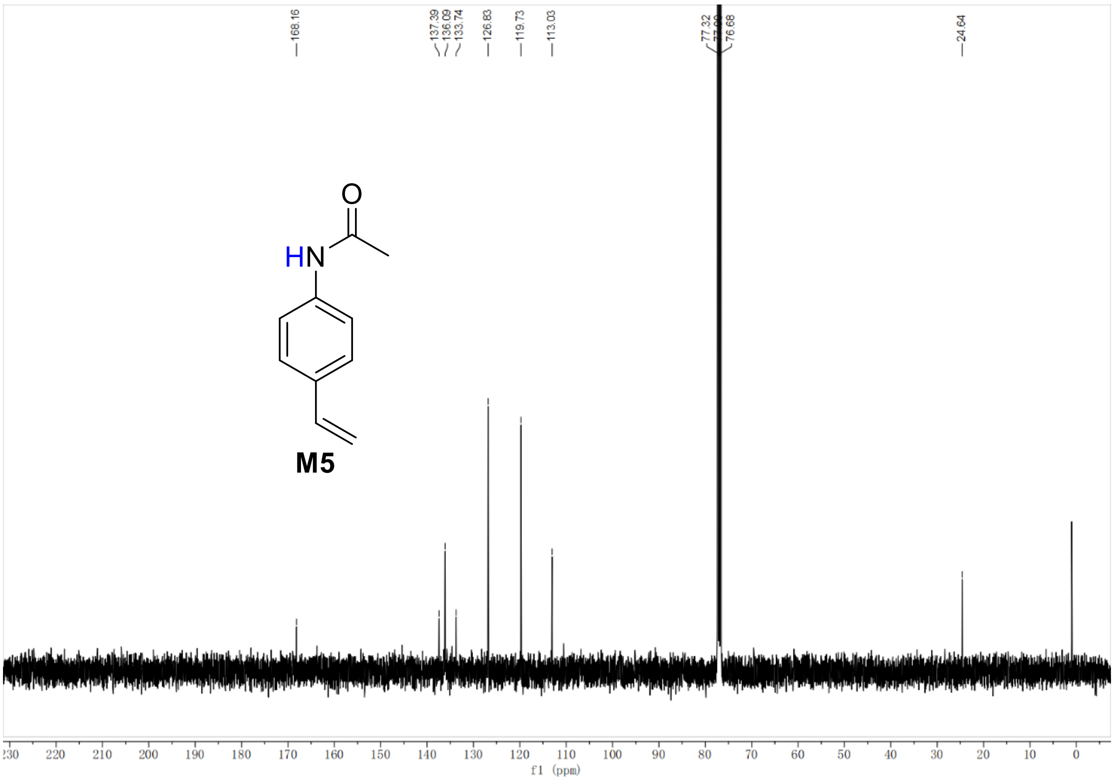

^{1}H NMR spectrum of **P1** (CD_2Cl_2, 400 MHz)

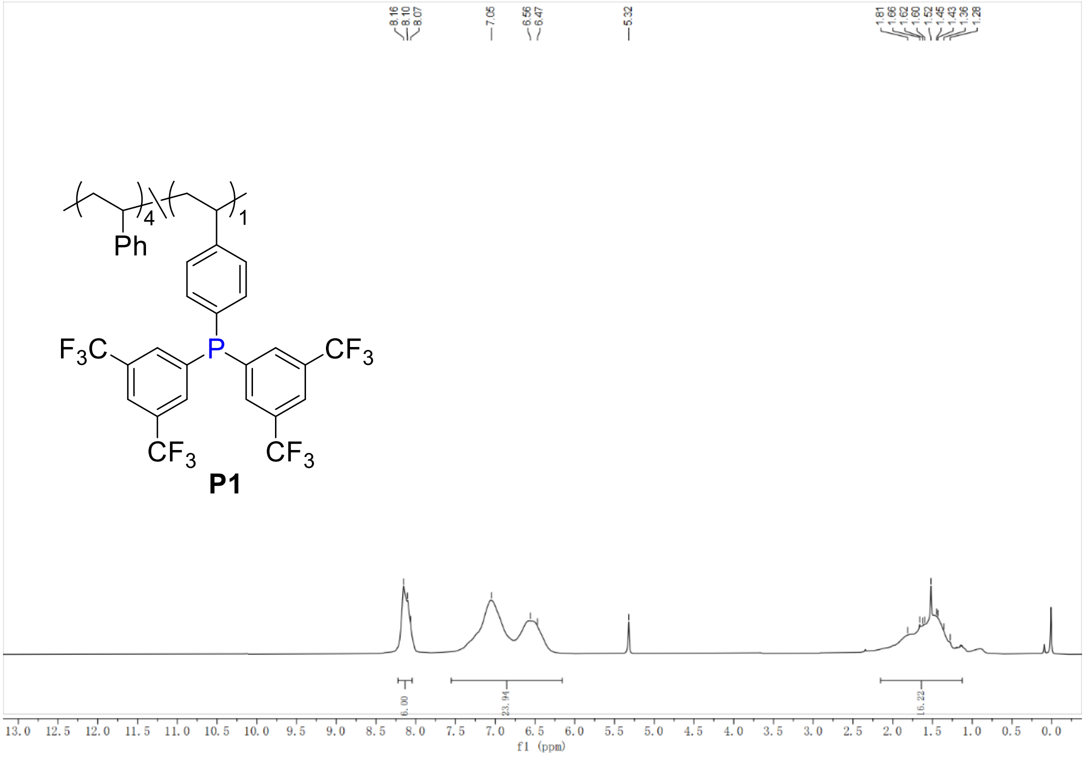

^{19}F NMR spectrum of **P1** ($CDCl_3$, 376 MHz)

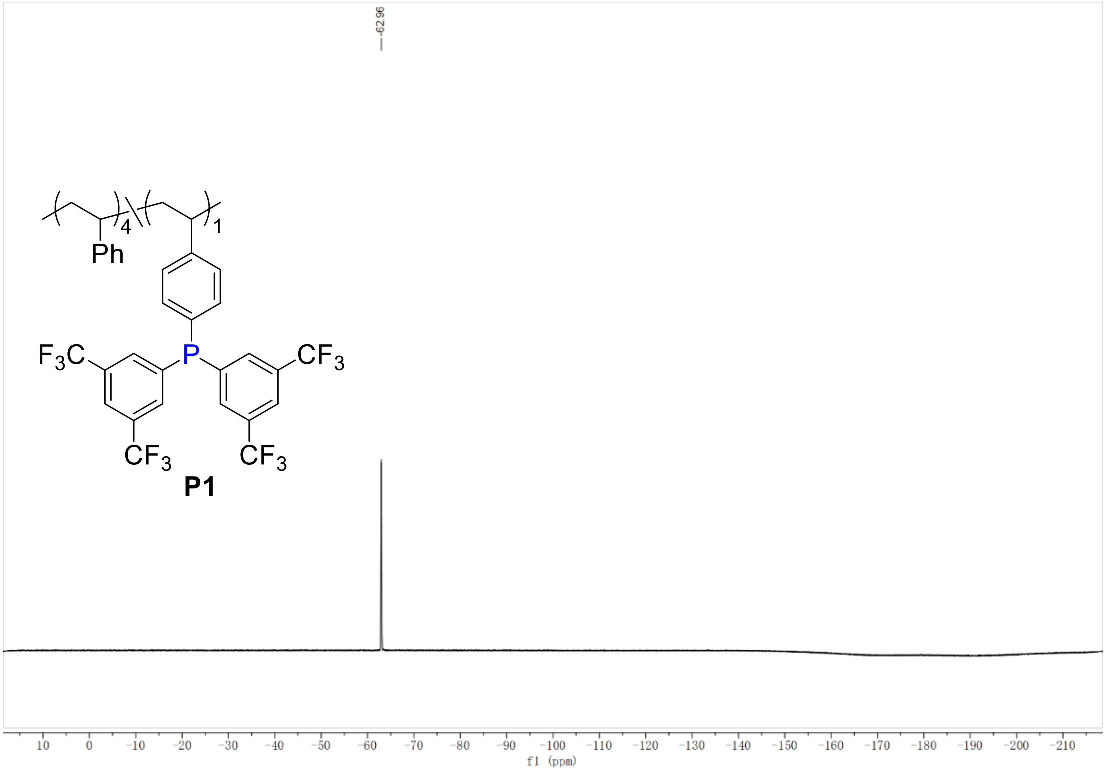

^{31}P NMR spectrum of **P1** ($CDCl_3$, 162 MHz)

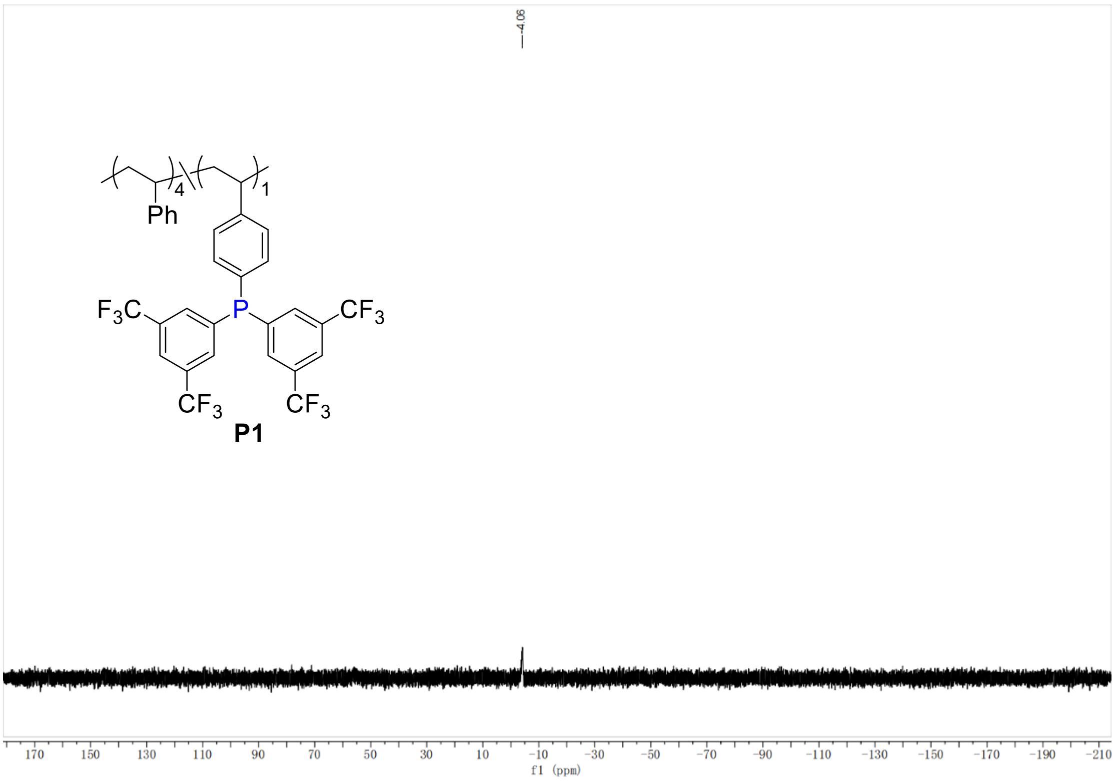

GPC curve of **P1**

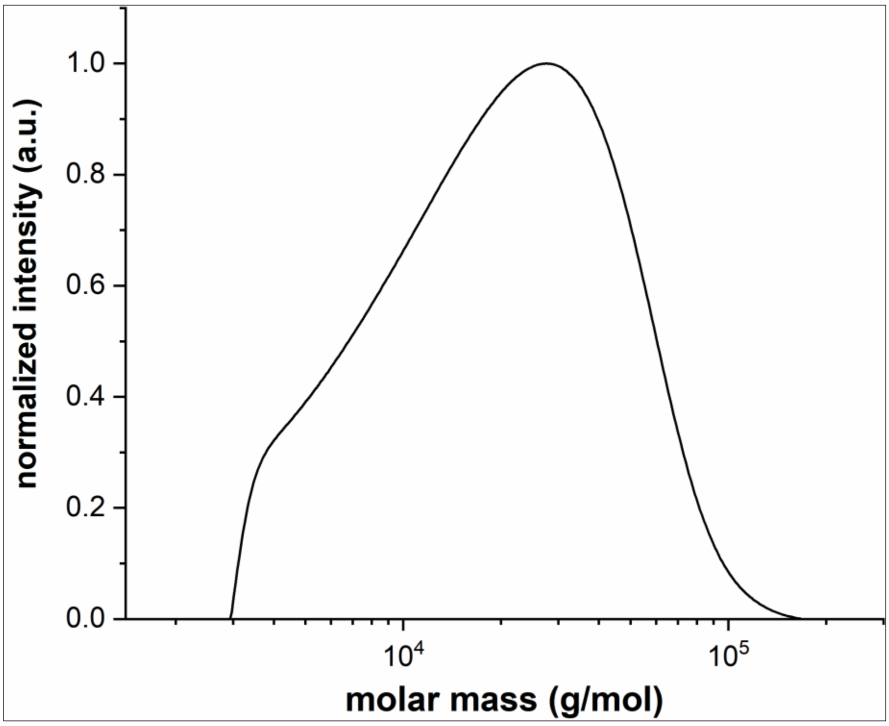

^{1}H NMR spectrum of **P2** (CD_2Cl_2, 400 MHz)

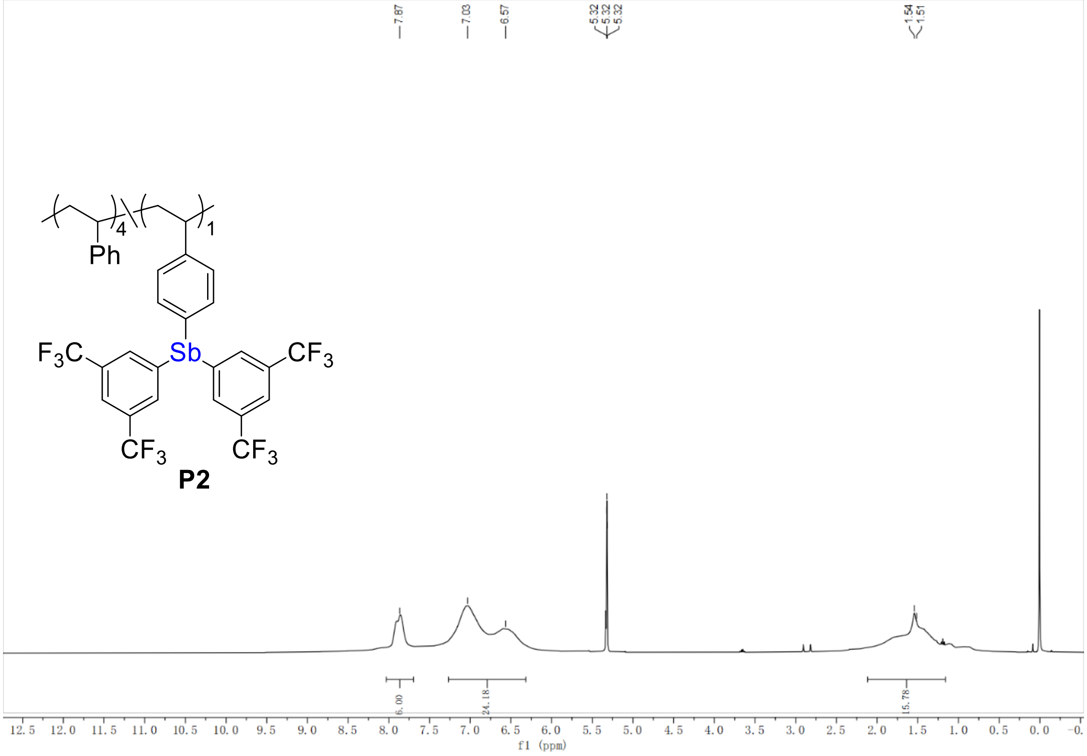

^{19}F NMR spectrum of **P2** ($CDCl_3$, 376 MHz)

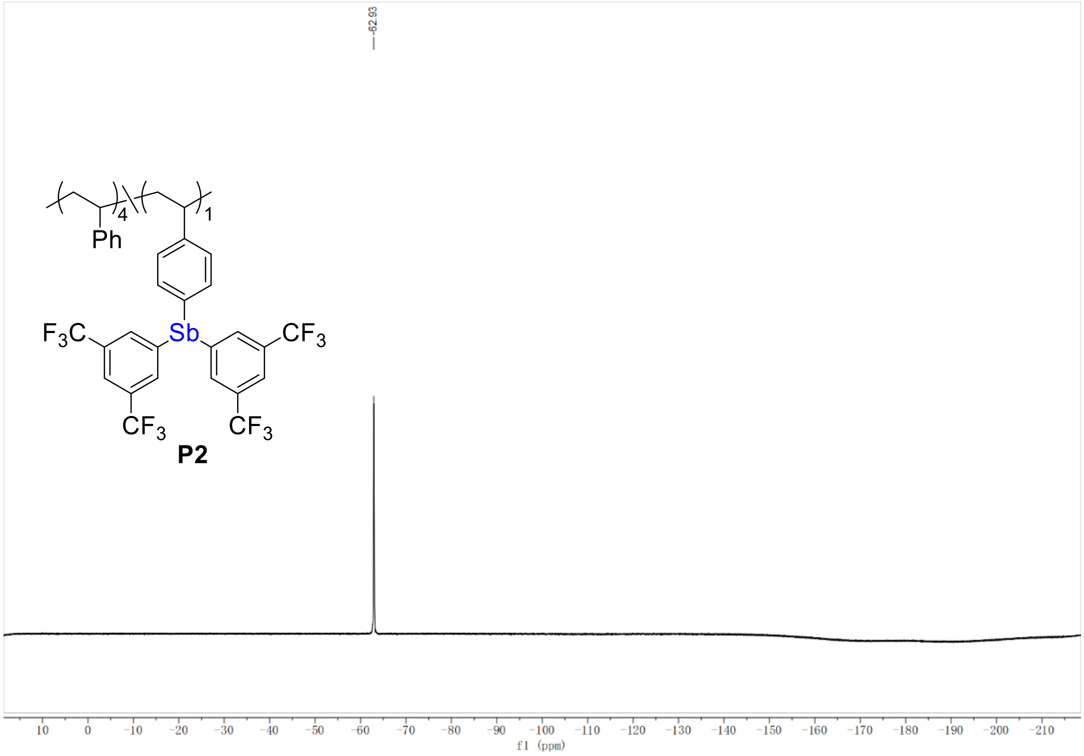

GPC curve of **P2**

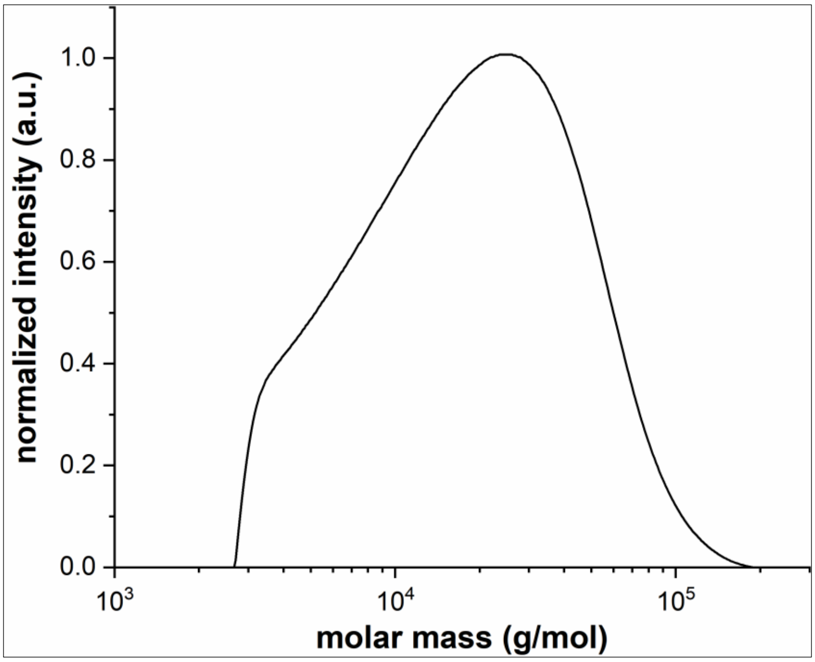

^{1}H NMR spectrum of **P3** (CD_2Cl_2, 400 MHz)

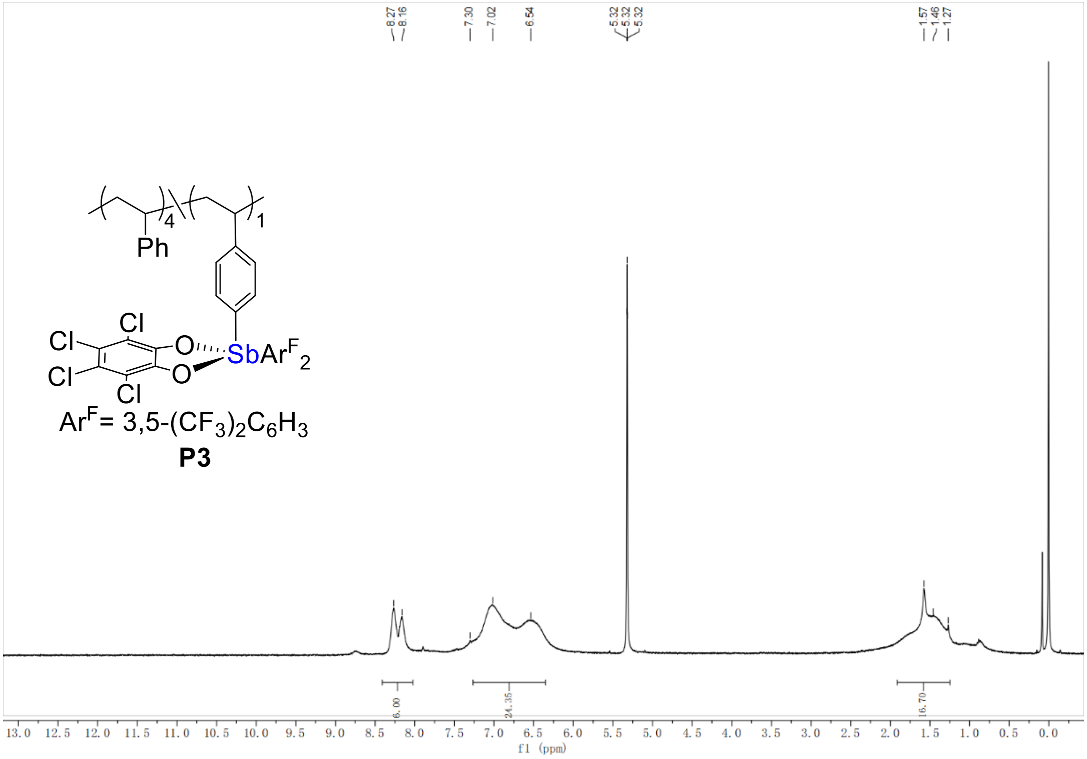

^{19}F NMR spectrum of **P3** ($CDCl_3$, 376 MHz)

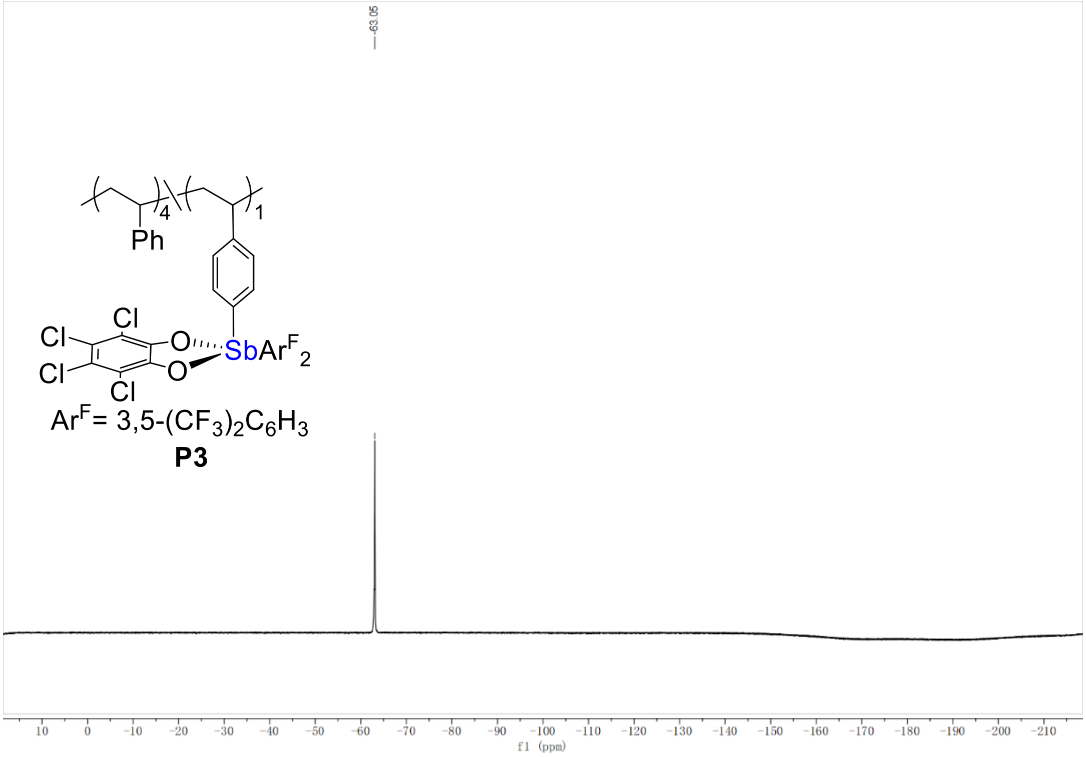

GPC curve of **P3**

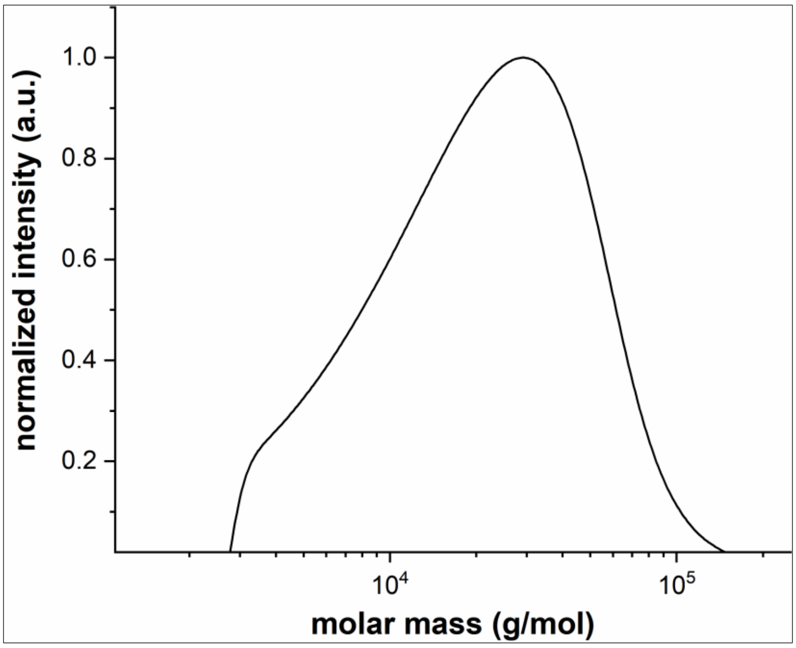

^{1}H NMR spectrum of **P4** ($CDCl_3$, 400 MHz)

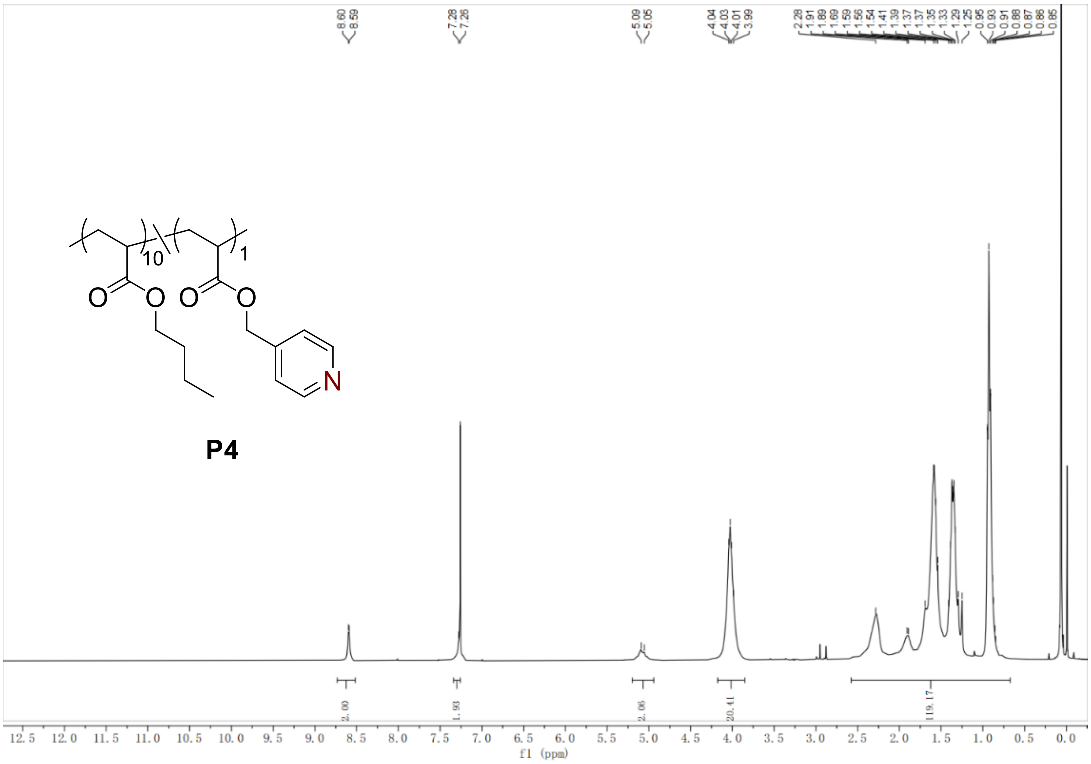

GPC curve of **P4**

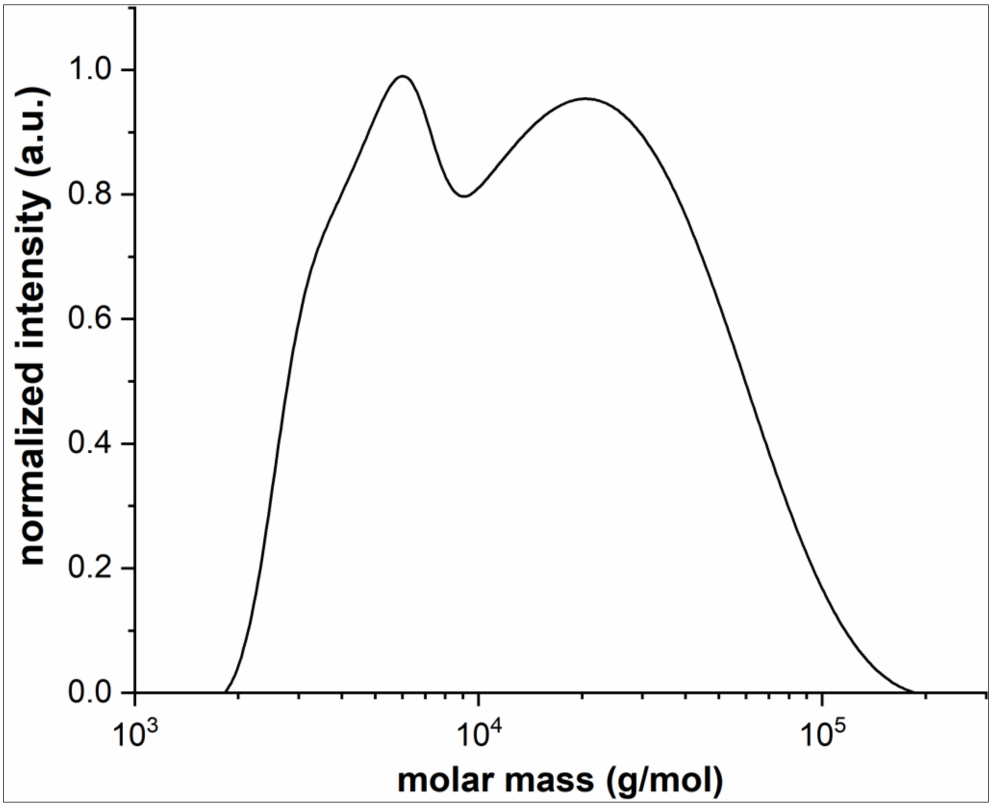

^{1}H NMR spectrum of **P4’** ($CDCl_3$, 400 MHz)

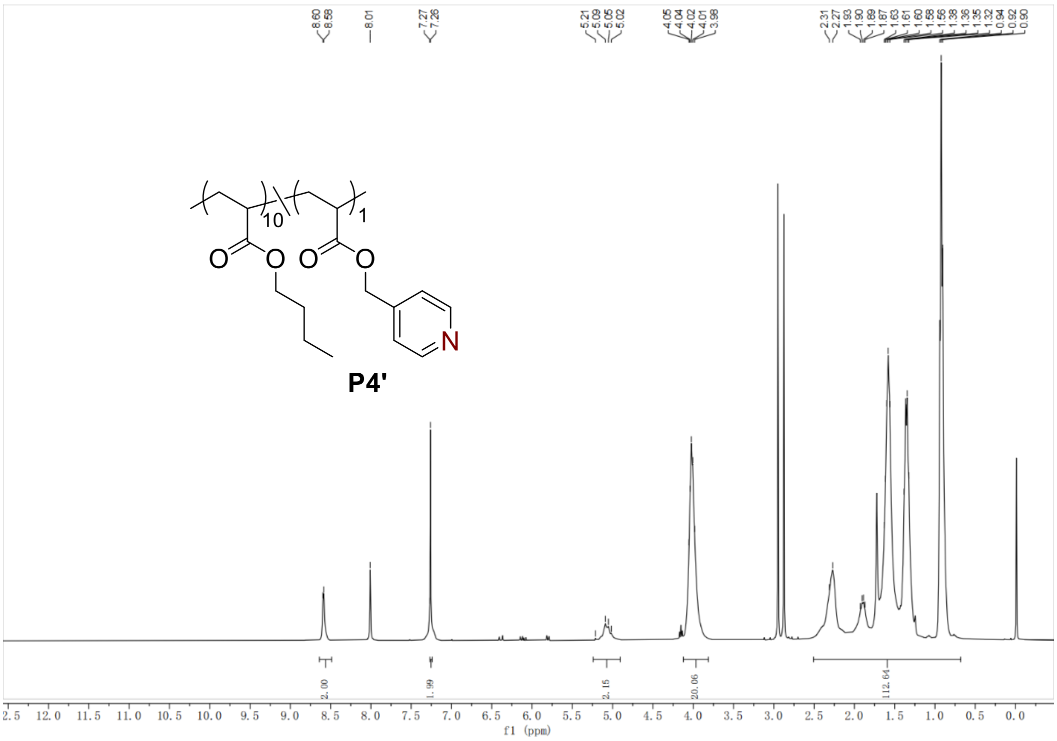

GPC curve of **P4’**

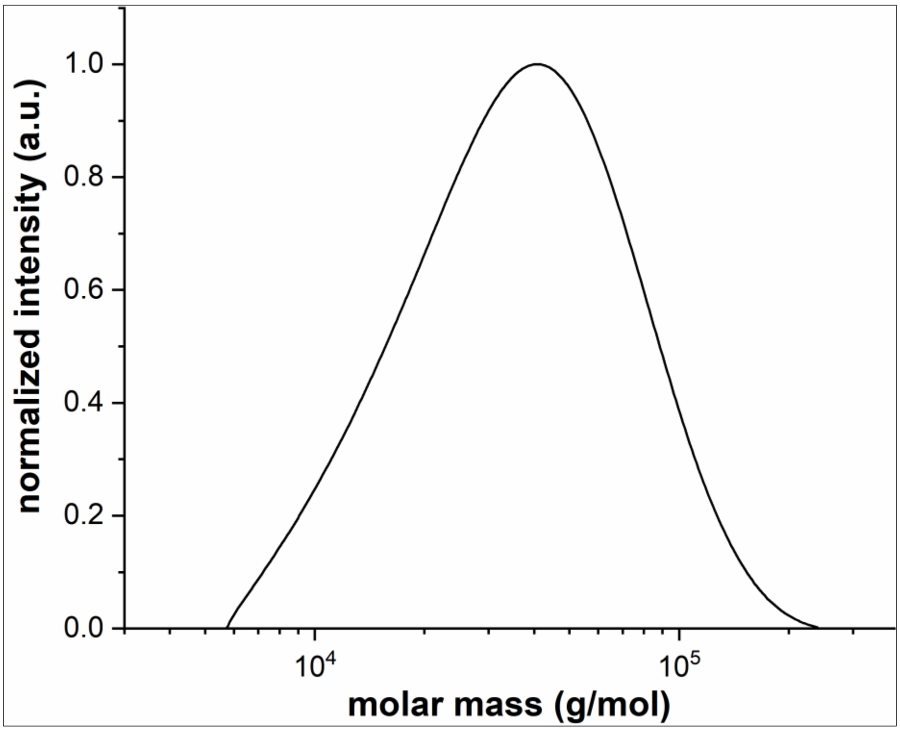

^{1}H NMR spectrum of **P5** (CD_2Cl_2, 400 MHz)

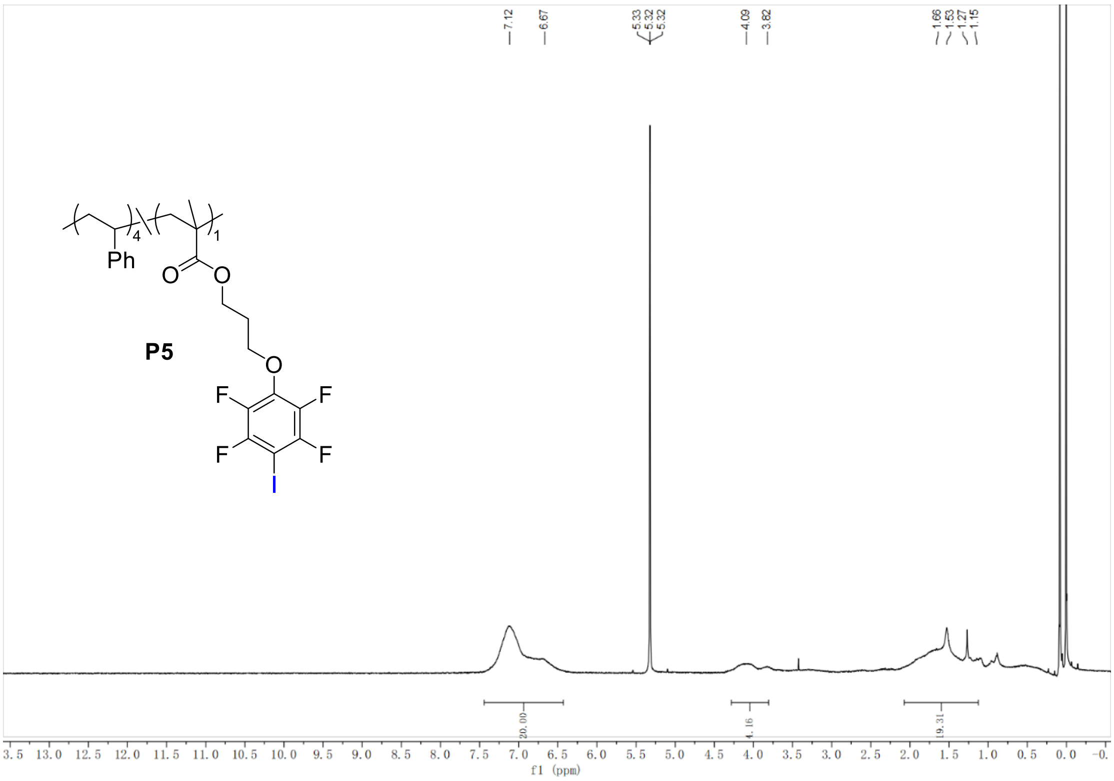

^{19}F NMR spectrum of **P5** ($CDCl_3$, 376 MHz)

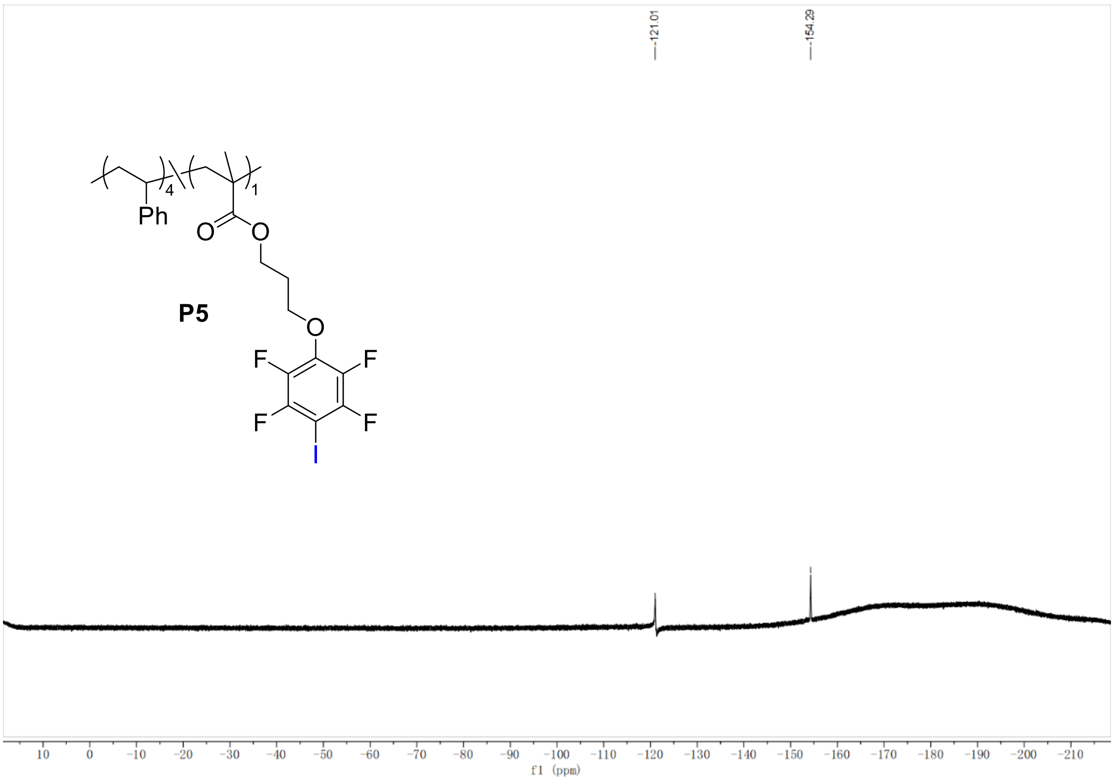

GPC curve of **P5**

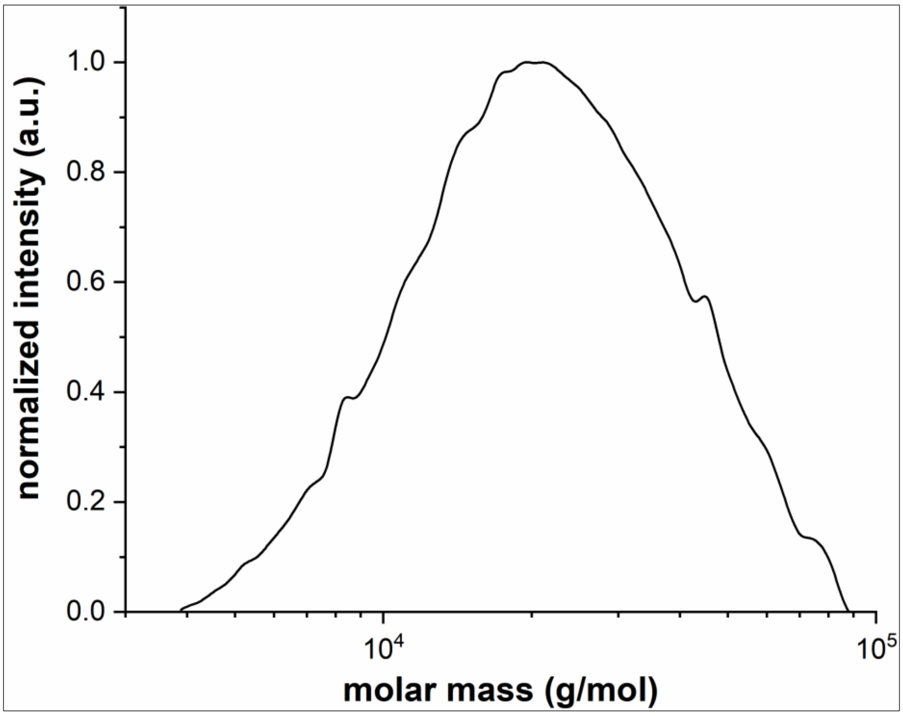

^{1}H NMR spectrum of **P6** ($CDCl_3$, 400 MHz)

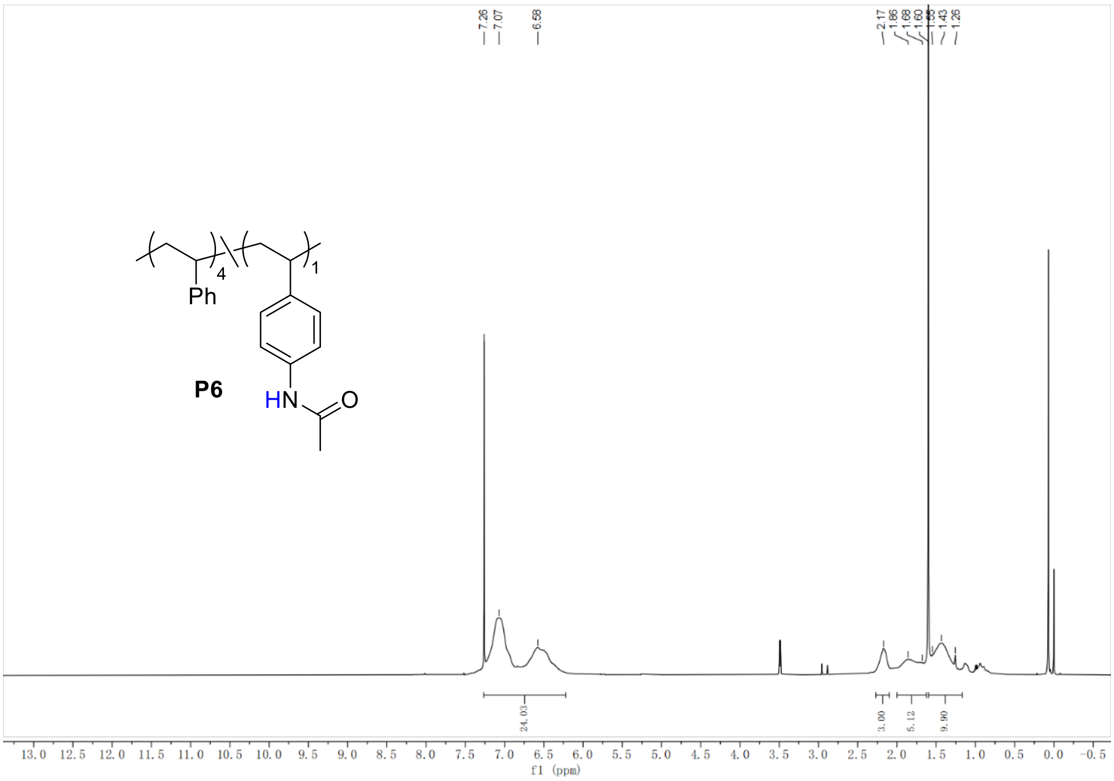

GPC curve of **P6**

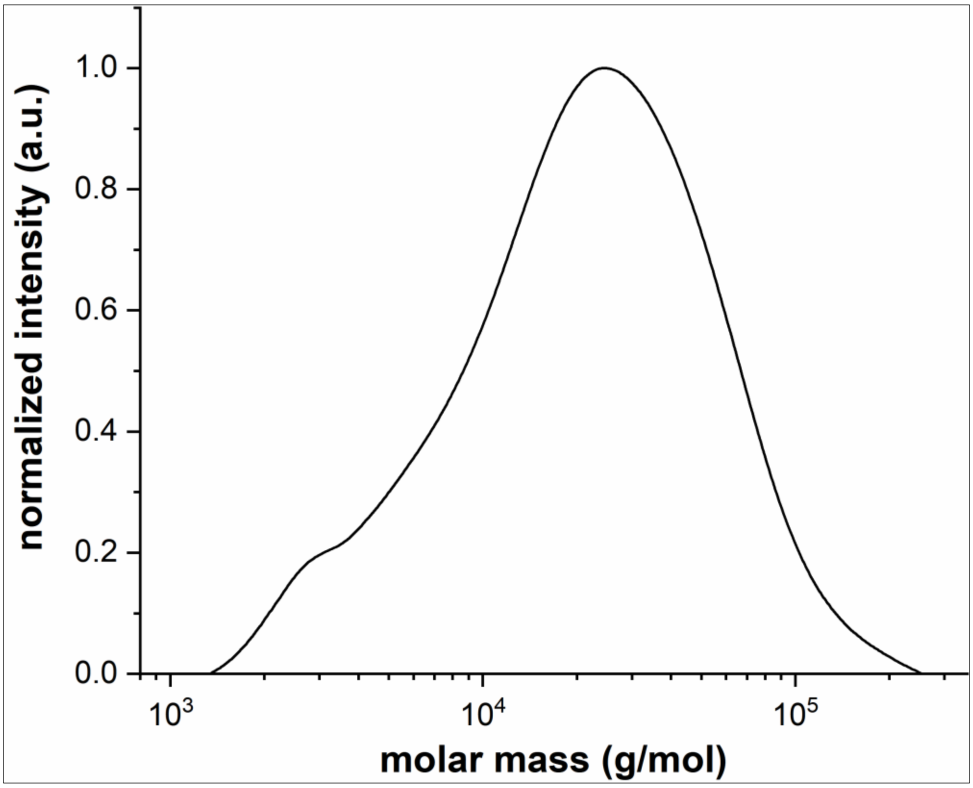

^1H NMR spectrum of **P^{III}-PNs** ($CDCl_3$, 400 MHz)

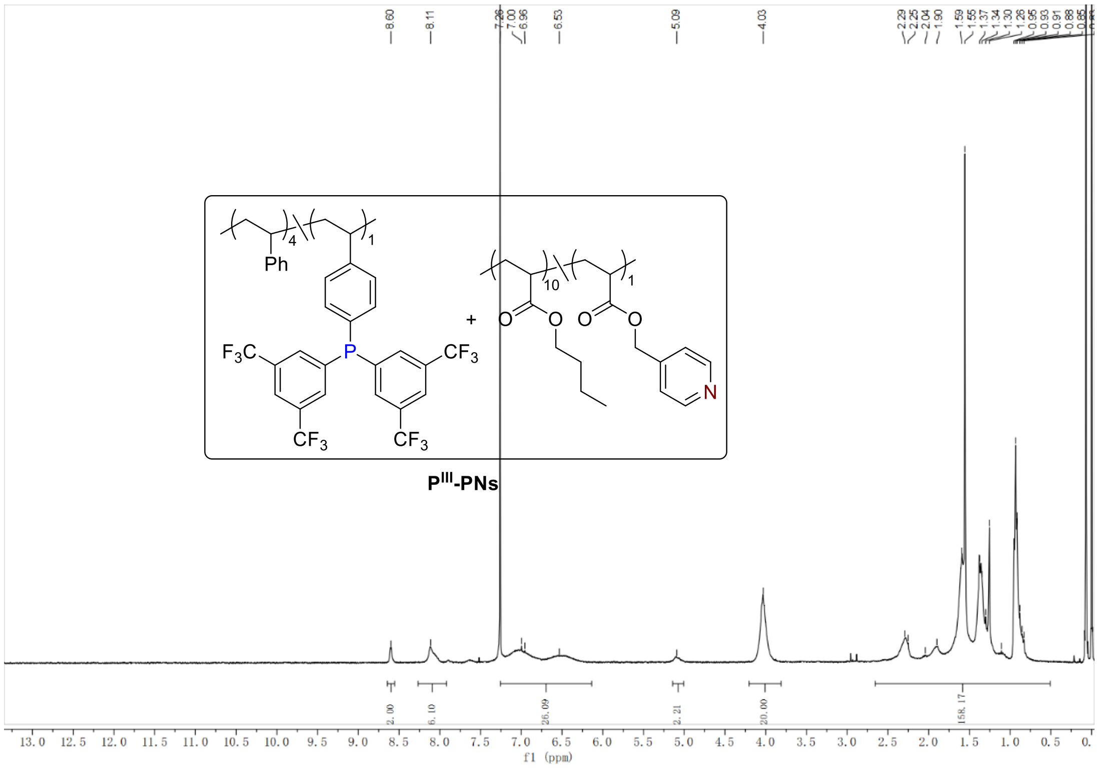

^{19}F NMR spectrum of **P^{III}-PNs** ($CDCl_3$, 376 MHz)

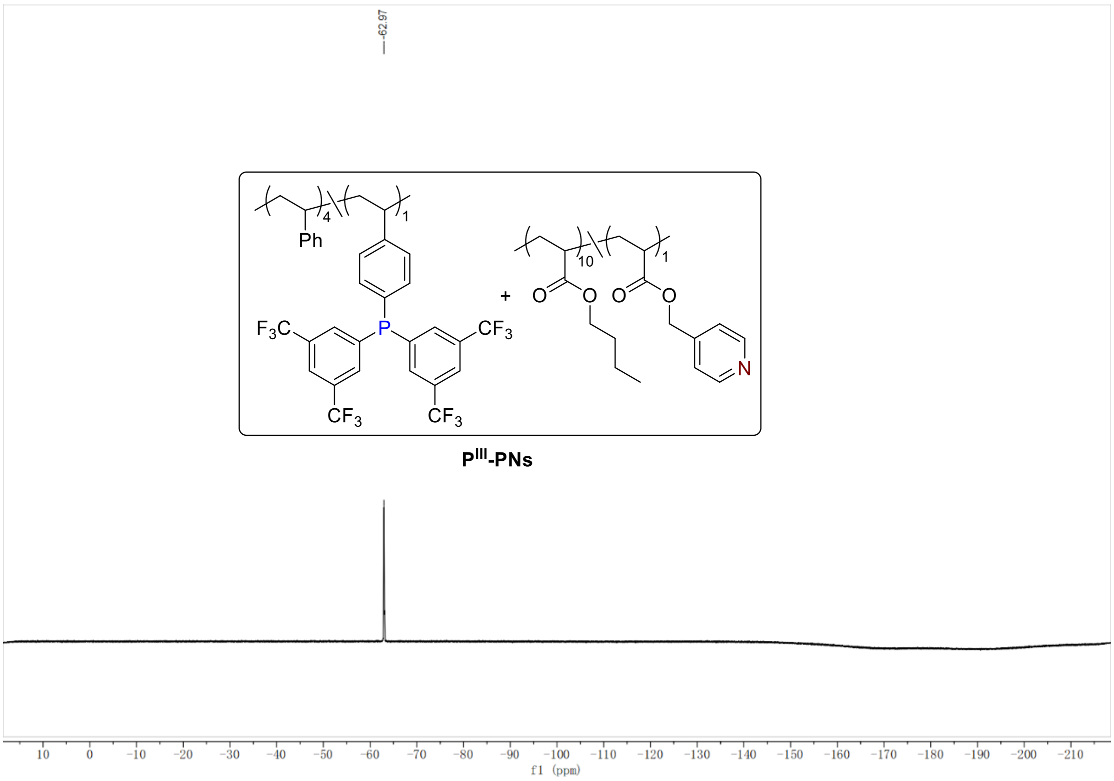

^{1}H NMR spectrum of **Sb^{III}-PNs** ($CDCl_3$, 400 MHz)

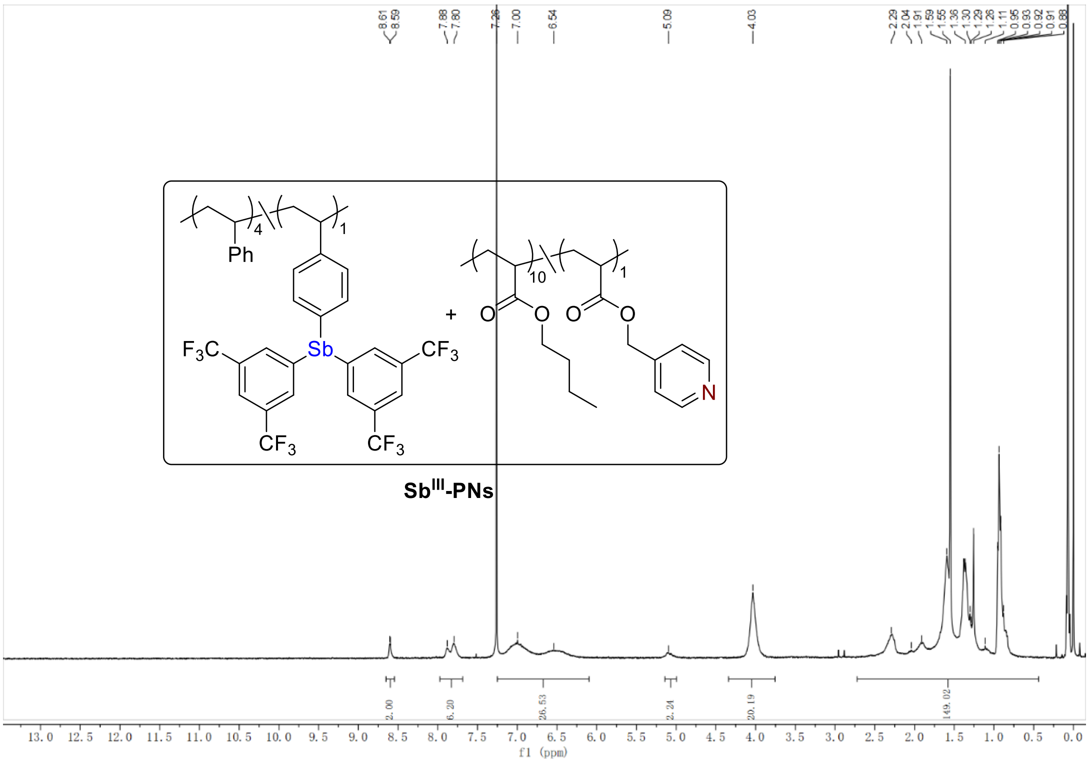

^{19}F NMR spectrum of **Sb^{III}-PNs** ($CDCl_3$, 376 MHz)

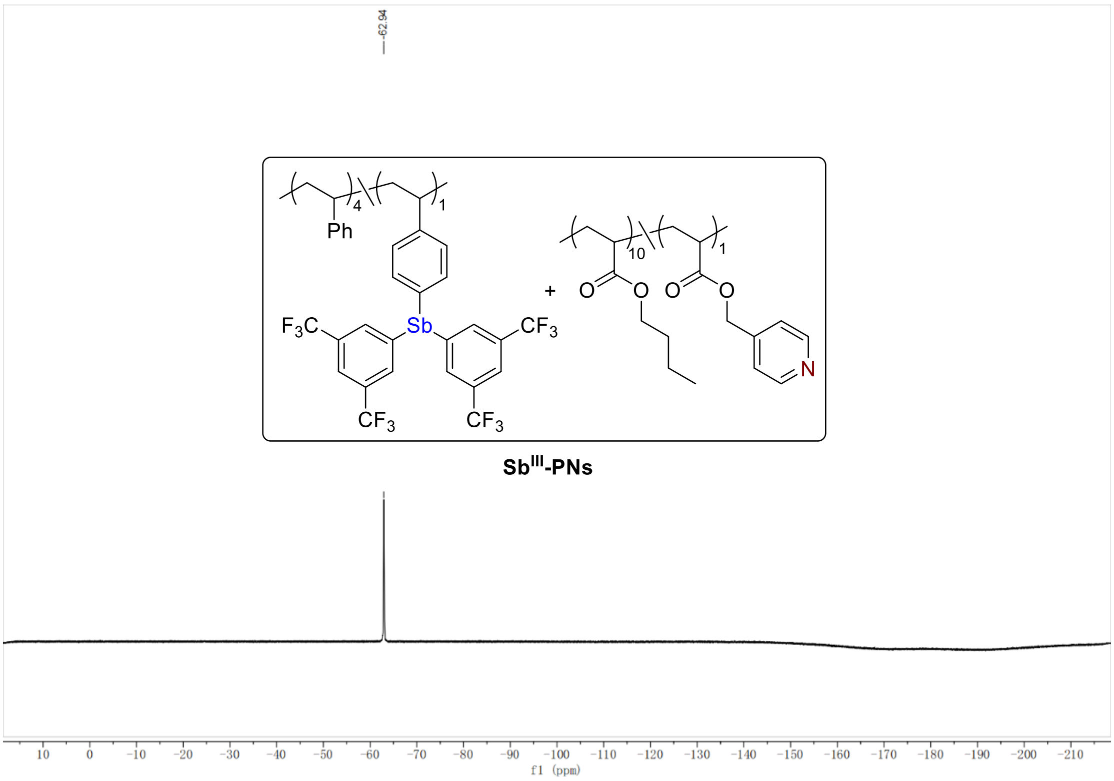

^{1}H NMR spectrum of **Sb^{III}-PNs'** ($CDCl_3$, 400 MHz)

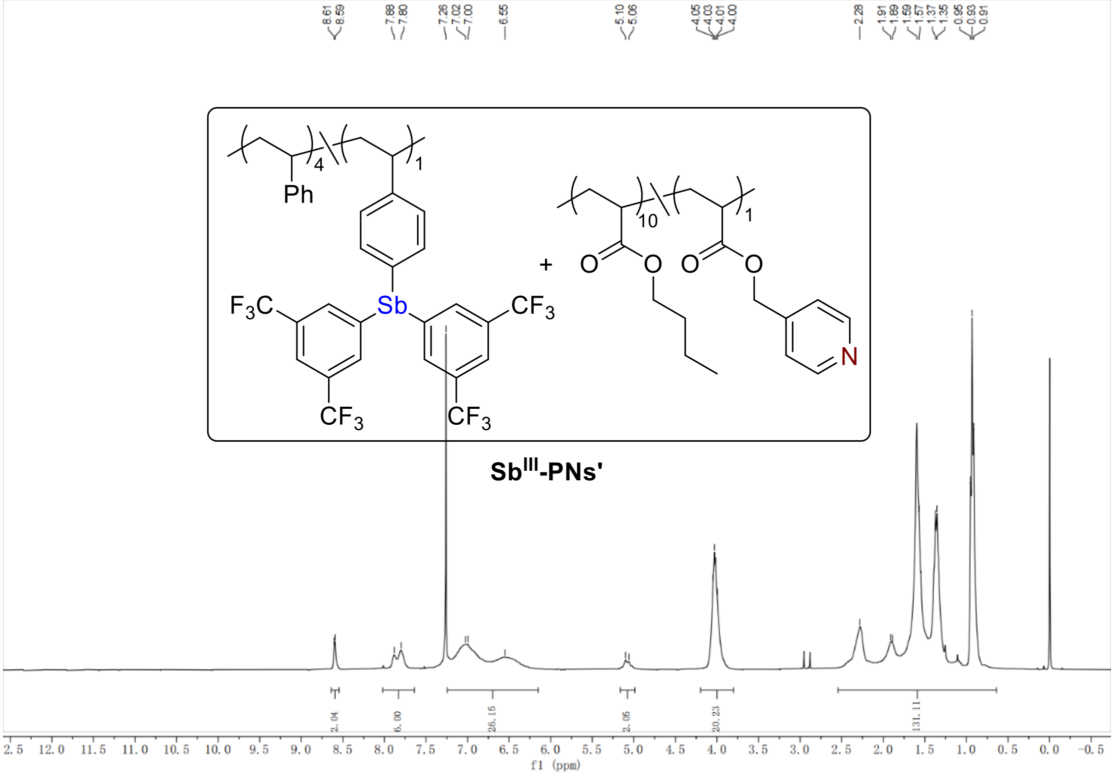

^{19}F NMR spectrum of **Sb^{III}-PNs'** ($CDCl_3$, 376 MHz)

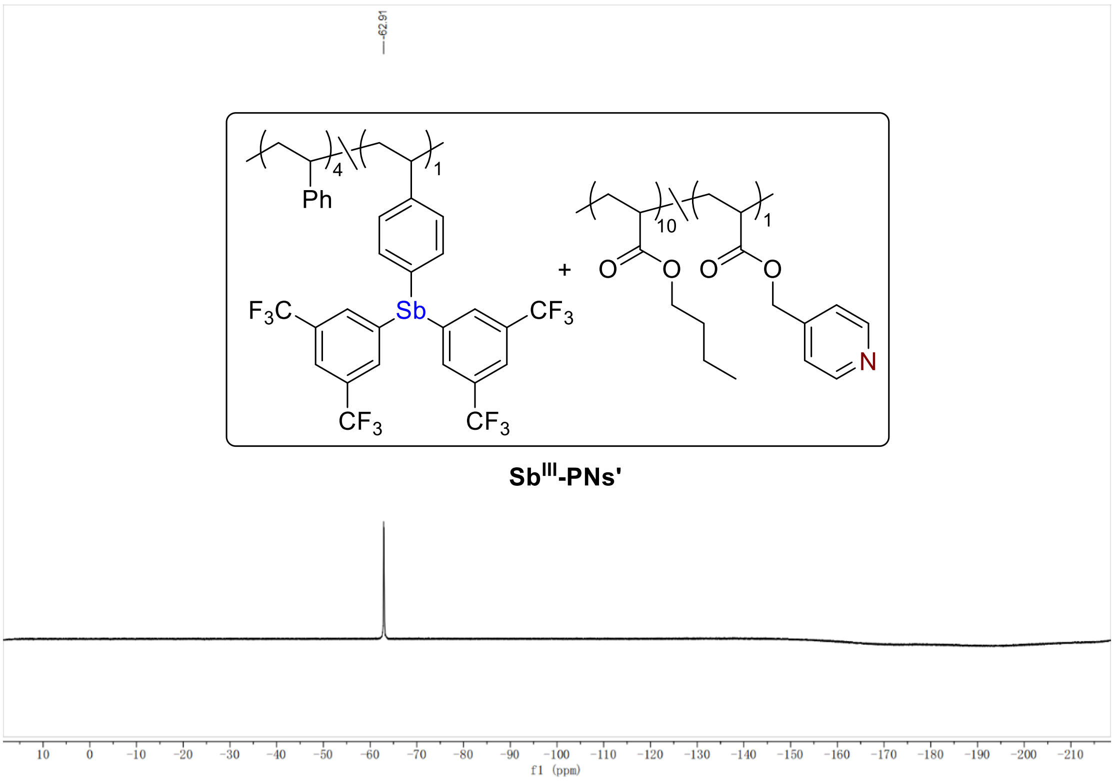

^{1}H NMR spectrum of **Sb^{V}-PNs** (CD_2Cl_2, 400 MHz)

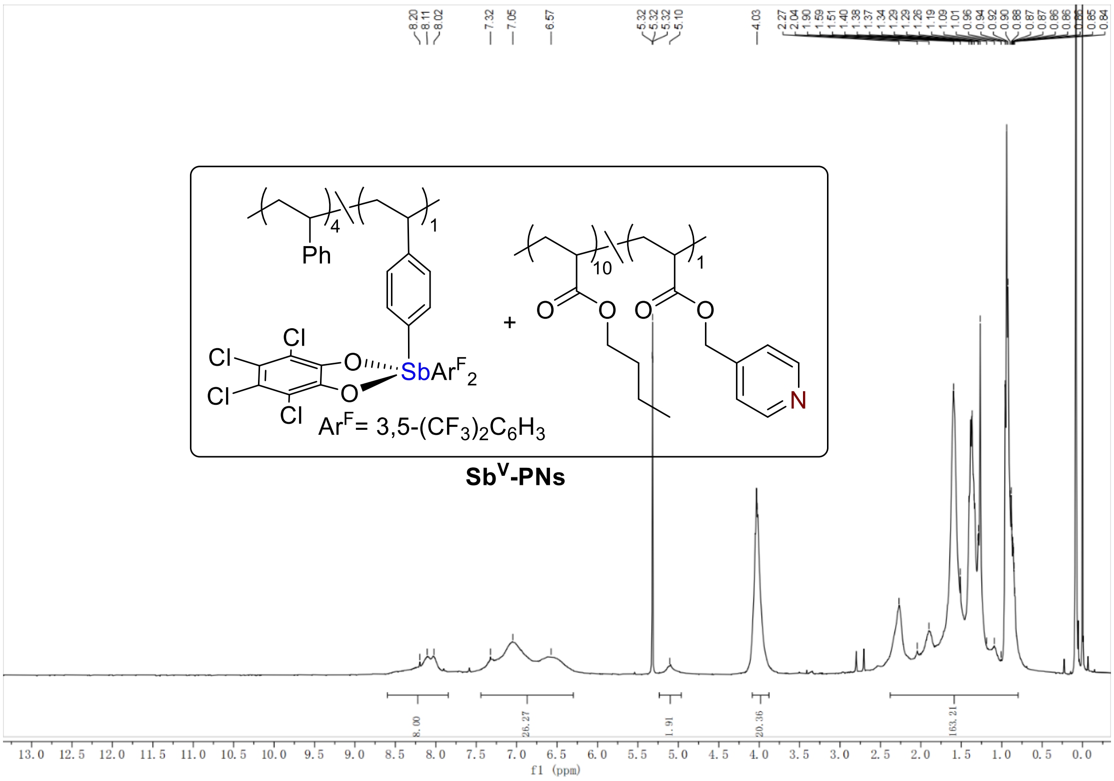

^{19}F NMR spectrum of **Sb^{V}-PNs** ($CDCl_3$, 376 MHz)

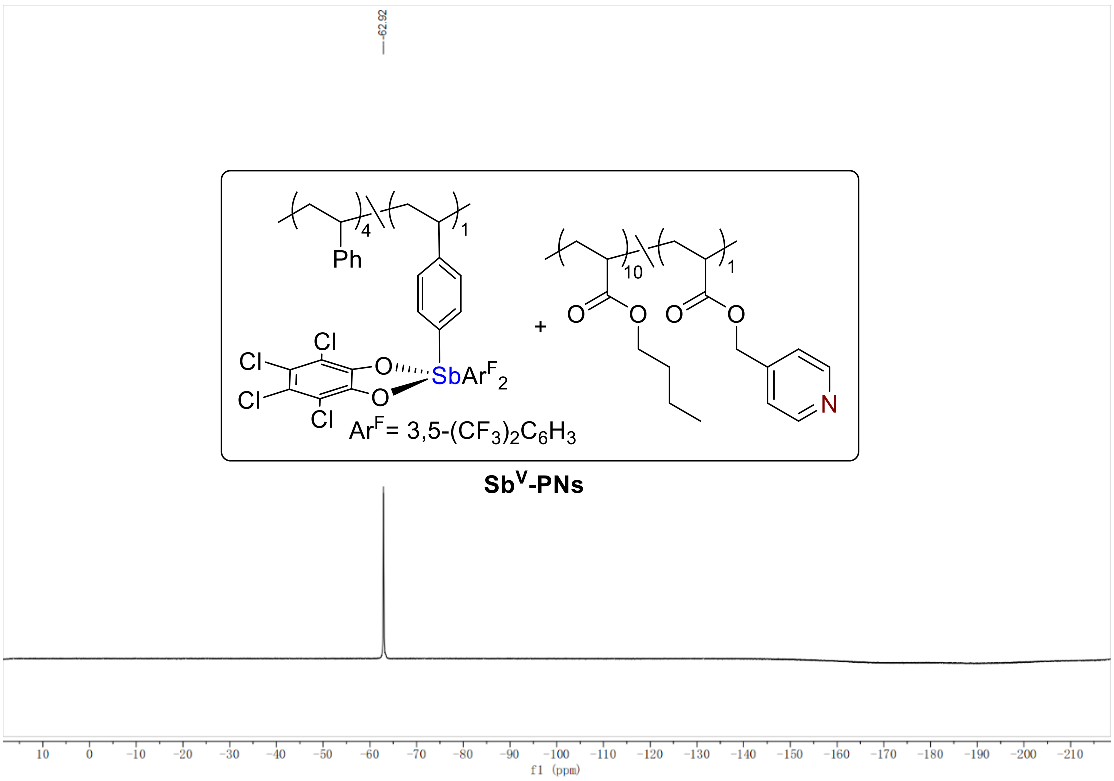

^{1}H NMR spectrum of **XB-PNs** ($CDCl_3$, 400 MHz)

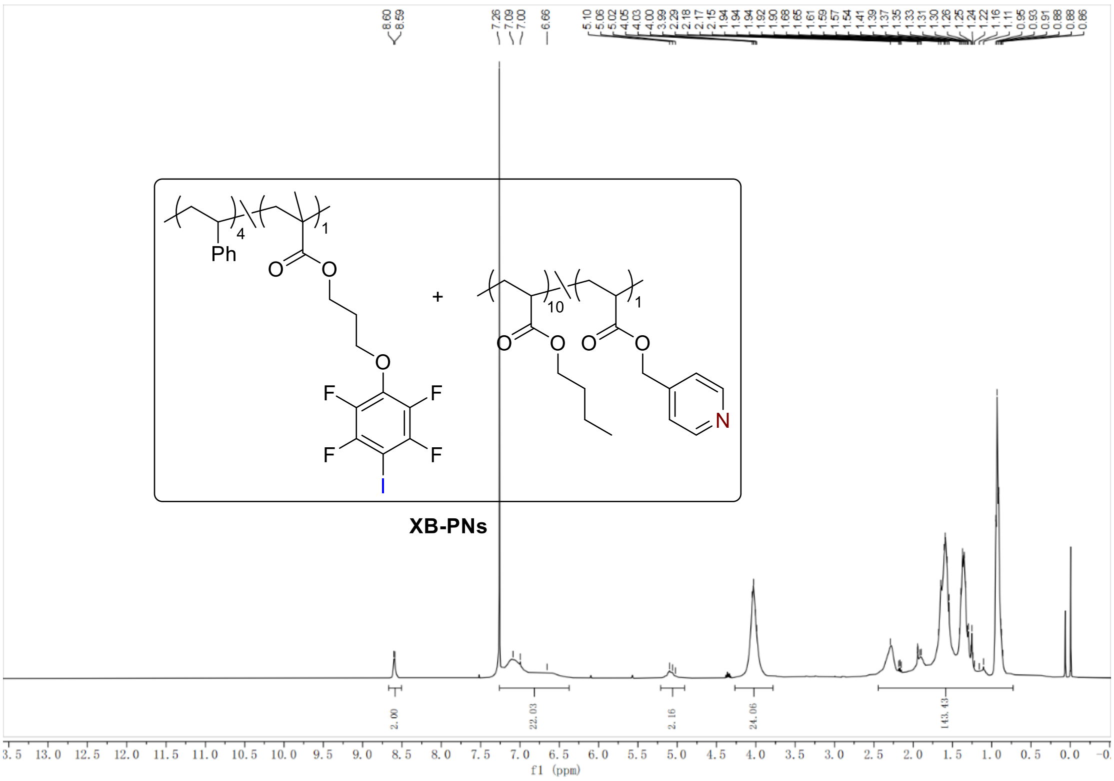

^{19}F NMR spectrum of **XB-PNs** ($CDCl_3$, 376 MHz)

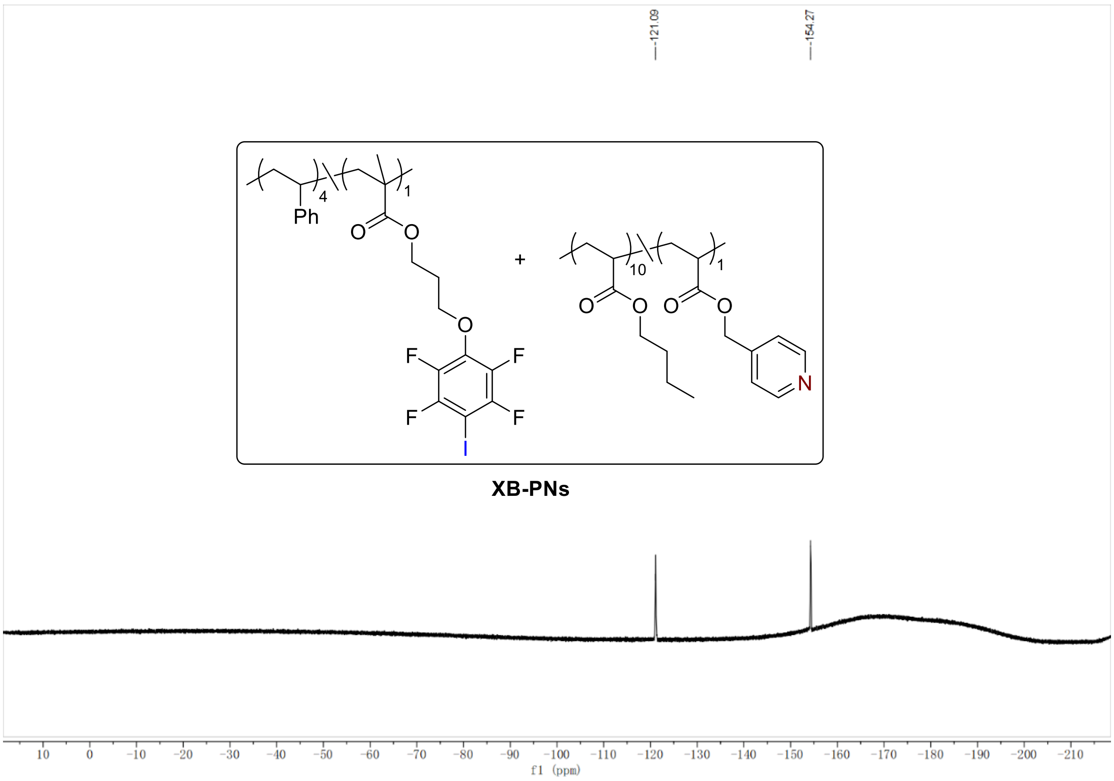

^{1}H NMR spectrum of **HB-PNs** ($CDCl_3$, 400 MHz)

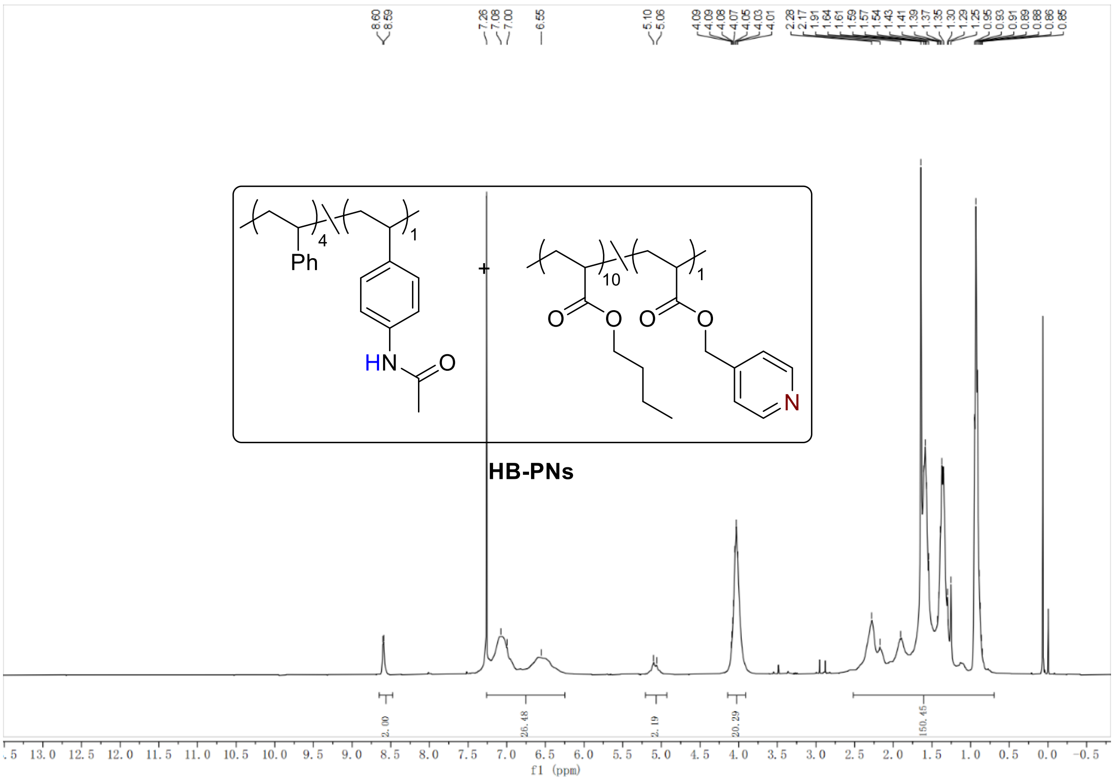

^{1}H NMR spectrum of **Sb$^{V/III}$-PNs** ($CDCl_3$, 400 MHz)

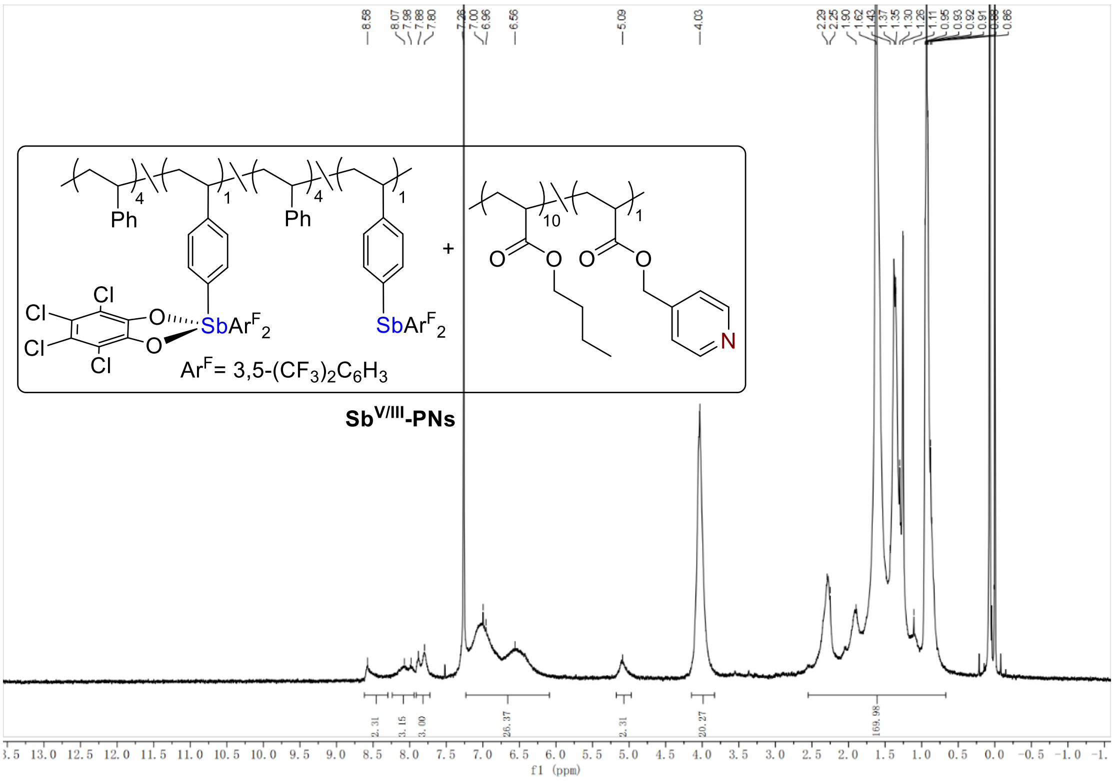

^{19}F NMR spectrum of **Sb$^{V/III}$-PNs** ($CDCl_3$, 376 MHz)

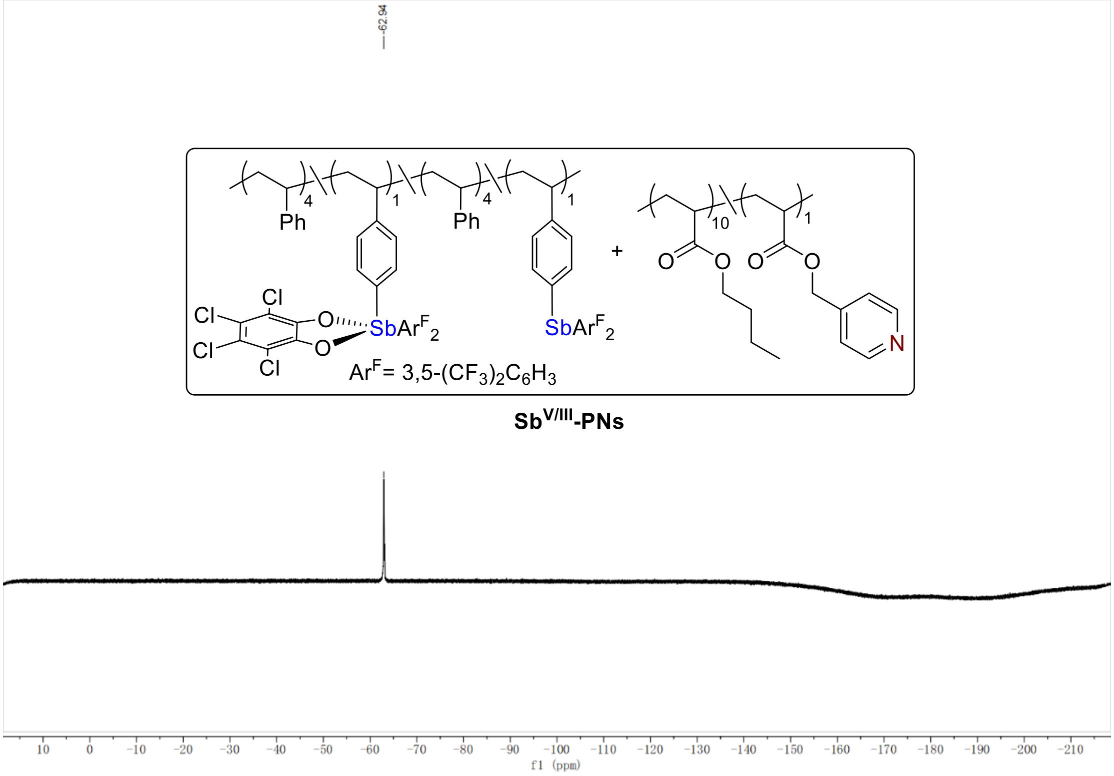

14. Reference

[1] H. Guo, R. Puttreddy, T. Salminen, A. Lends, K. Jaudzems, H. Zeng, A. Priimagi, *Nat. Commun.* **2022**, *13*, 7436.

[2] A. Vanderkooy, M. S. Taylor, *J. Am. Chem. Soc.* **2015**, *137*, 5080–5086.

[3] B. Yang, X.-H. Xu, F.-L. Qing, *Org. Lett.* **2015**, *17*, 1906–1909.

[4] P. Thordarson, *Chem. Soc. Rev.* **2011**, *40*, 1305–1323.

[5] D. B. Hibbert, P. Thordarson, *Chem. Commun.* **2016**, *52*, 12792–12805.

[6] H. Lee, J. Kim, M. Lee, J. Kang, *Chem. Rev.* **2025**, *125*, 11379–11425.

[7] S. Ge, Y.-H. Tsao, C. M. Evans, *Nat. Commun.* **2023**, *14*, 7244.

[8] P. J. Stephens, C. F. Chabalowski, F. J. Devlin, K. J. Jalkanen, *J. Phys. Chem.* **1994**, *98*, 11623–11627.

[9] F. Weigend, R. Ahlrichs, *Phys. Chem. Chem. Phys.* **2005**, *7*, 3297–3305.

[10] S. Grimme, J. Antony, S. Ehrlich, H. Krieg, *J. Chem. Phys.* **2010**, *132*, 154104.

[11] S. Grimme, S. Ehrlich, L. Goerigk, *J. Comp. Chem.* **2011**, *32*, 1456–1465.

[12] Gaussian 16, Revision A.03, M. J. Frisch, G. W. Trucks, H. B. Schlegel, G. E.

Scuseria, M. A. Robb, J. R. Cheeseman, G. Scalmani, V. Barone, G. A. Petersson, H. Nakatsuji, X. Li, M. Caricato, A. V. Marenich, J. Bloino, B. G. Janesko, R. Gomperts, B. Mennucci, H. P. Hratchian, J. V. Ortiz, A. F. Izmaylov, J. L. Sonnenberg, D. Williams-Young, F. Ding, F. Lipparini, F. Egidi, J. Goings, B. Peng, A. Petrone, T. Henderson, D. Ranasinghe, V. G. Zakrzewski, J. Gao, N. Rega, G. Zheng, W. Liang, M. Hada, M. Ehara, K. Toyota, R. Fukuda, J. Hasegawa, M. Ishida, T. Nakajima, Y. Honda, O. Kitao, H. Nakai, T. Vreven, K. Throssell, J. A. Montgomery, J. J. E. Peralta, F. Ogliaro, M. J. Bearpark, J. J. Heyd, E. N. Brothers, K. N. Kudin, V. N. Staroverov, T. A. Keith, R. Kobayashi, J. Normand, K. Raghavachari, A. P. Rendell, J. C. Burant, S. S. lyengar, J. Tomasi, M. Cossi, J. M. Millam, M. Klene, C. Adamo, R. Cammi, J. W. Ochterski, R. L. Martin, K. Morokuma, O. Farkas, J. B. Foresman, D. J. Fox, Gaussian, Inc., Wallingford CT, 2016.

[13] T. Lu, F. Chen, *J. Comput. Chem.* **2011**, *33*, 580–592.

[14] T. Lu, *J. Chem. Phys.* **2024,** *161*, 082503.

[15] J. Zhang, T. Lu, *Phys. Chem. Chem. Phys.* **2021**, *23*, 20323

[16] T. Lu, F. Chen, *J. Mol. Graph. Model.* **2012**, *38*, 314–323.

[17] W. Humphrey, A. Dalke, K. Schulten, *J. Mol. Graph. Model.* **1996**, *14*, 33–38.
